# Vortex fluidic induced mass transfer across immiscible phases†‡

**DOI:** 10.1039/d1sc05829k

**Published:** 2022-01-31

**Authors:** Matt Jellicoe, Aghil Igder, Clarence Chuah, Darryl B. Jones, Xuan Luo, Keith A. Stubbs, Emily M. Crawley, Scott J. Pye, Nikita Joseph, Kasturi Vimalananthan, Zoe Gardner, David P. Harvey, Xianjue Chen, Filomena Salvemini, Shan He, Wei Zhang, Justin M. Chalker, Jamie S. Quinton, Youhong Tang, Colin L. Raston

**Affiliations:** Flinders Institute for Nanoscale Science and Technology, College of Science and Engineering, Flinders University Bedford Park SA 5042 Australia colin.raston@flinders.edu.au; School of Molecular Sciences, The University of Western Australia 35 Stirling Highway Crawley WA 6009 Australia; School of Environmental and Life Sciences, The University of Newcastle Callaghan New South Wales 2308 Australia; Australian Nuclear Science and Technology Organization New Illawara Road, Lucas Heights NSW Australia; Department of Food Science and Engineering, School of Chemistry Chemical Engineering, Guangzhou University Guangzhou 510006 China; Centre for Marine Bioproducts Development, College of Medicine and Public Health, Flinders University Adelaide SA 5042 Australia; Flinders Microscopy and Microanalysis (FMMA), College of Science and Engineering, Flinders University GPO Box 2100 Adelaide South Australia 5001 Australia

## Abstract

Mixing immiscible liquids typically requires the use of auxiliary substances including phase transfer catalysts, microgels, surfactants, complex polymers and nano-particles and/or micromixers. Centrifugally separated immiscible liquids of different densities in a 45° tilted rotating tube offer scope for avoiding their use. Micron to submicron size topological flow regimes in the thin films induce high inter-phase mass transfer depending on the nature of the two liquids. A hemispherical base tube creates a Coriolis force as a ‘spinning top’ (ST) topological fluid flow in the less dense liquid which penetrates the denser layer of liquid, delivering liquid from the upper layer through the lower layer to the surface of the tube with the thickness of the layers determined using neutron imaging. Similarly, double helical (DH) topological flow in the less dense liquid, arising from Faraday wave eddy currents twisted by Coriolis forces, impact through the less dense liquid onto the surface of the tube. The lateral dimensions of these topological flows have been determined using ‘molecular drilling’ impacting on a thin layer of polysulfone on the surface of the tube and self-assembly of nanoparticles at the interface of the two liquids. At high rotation speeds, DH flow also occurs in the denser layer, with a critical rotational speed reached resulting in rapid phase demixing of preformed emulsions of two immiscible liquids. ST flow is perturbed relative to double helical flow by changing the shape of the base of the tube while maintaining high mass transfer between phases as demonstrated by circumventing the need for phase transfer catalysts. The findings presented here have implications for overcoming mass transfer limitations at interfaces of liquids, and provide new methods for extractions and separation science, and avoiding the formation of emulsions.

## Abstract 2

Micron to submicron size Coriolis and Faraday wave induced high shear topological flow regimes in 45° titled rapidly rotating tubes result in high inter-phase mass transfer of immiscible liquids and spontaneous demixing.

## Graphical abstract

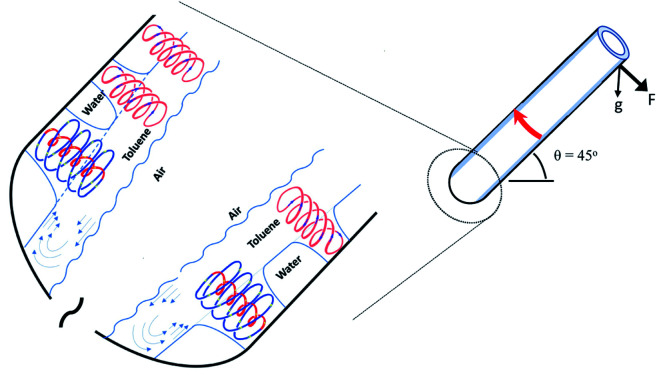

## Introduction

Mixing immiscible liquids is fundamentally important in process engineering for heat and mass transfer, in rendering mixtures of such liquids more homogeneous for promoting chemical and biochemical reactions, and in forming bicontinuous phases comprised of microstructures of immiscible liquids with high interfacial surface areas.^1^ This typically requires the use of auxiliary substances including phase transfer catalysts,^2^ microgels,^3–5^ surfactants, complex polymers and nano-particles^6^ and/or micromixers imparting hydrodynamic motion of two immiscible liquids, for example in the Archimedean screw effect,^7^ electric field induced interfacial nano-mixing,^8^ consecutive or cascade continuous stirred tank reactors (CSTRs)^9^ and rotating surfaces.^10–12^ The use of auxiliary substances can necessitate downstream purification processes though and can contribute to the generation of a waste stream. The use of immiscible liquids which are only sparingly soluble in each other can hinder reactions by maintaining physical separation of the reactants, and this needs to be addressed in designing synthetic methodology. However, there is potentially a drawback in avoiding the use of immiscible liquids, to overcome these limitations, in that it can lead to selecting liquids with reduced ability to dissolve the reactants. This is also applicable to contemporary developments in continuous flow synthesis where in addition to miscibility considerations, all reactants would usually be required to be dissolved in the liquid, leading to the use of non-traditional or non-optimal solvents. Device technologies capable of enhancing the miscibility of otherwise immiscible liquids offer exciting opportunities for chemical and biochemical processing, negating the need for the use of the aforementioned auxiliary substances and providing unique surface tension for materials processing.^9^ Phase transfer catalysts bind reagents through non-covalent interactions and deliver them across phase boundaries^2^ whereas microgels can compartmentalize immiscible liquids with enrichment of the corresponding phase or indeed homogenous phase formation^3–5^ but require heating post-processing for phase separation and reaction work up^5^.

**Fig. 1:**
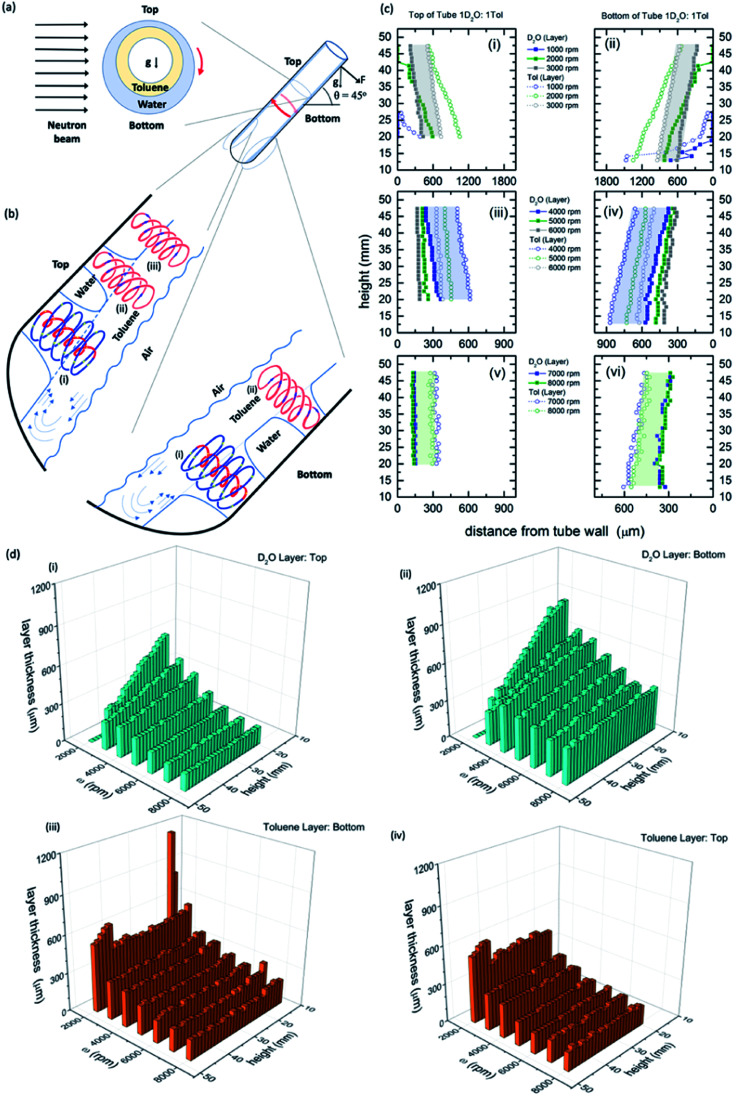
Neutron imaging derived film thicknesses and topological fluid flows. (a) Cartoon of the relative film thickness on the upper and lower side of the rapidly rotating hemispherical base quartz tube (20 mm OD, 17.5 mm ID). Here g denotes the direction of gravity or its projection within the cross sectional plane. (b) Fluid flows present in the films relative to the top and the bottom of the tube, as defined, with spinning top and double helical topological fluid flows, (i) and (ii) respectively, in the toluene layer impacting through the water layer onto the surface of the quartz tube titled at 45°, and where there is no distinction in the double helical flow for the two immiscible layers, (iii), where the thickness of the layers along the length of the top of the tube is constant (see Fig. 2). (c) Film thicknesses derived from neutron imaging with the direction of the neutron beam depicted in (a), for a 1 : 1 mixture of D_2_O and toluene at the top (upper side) and bottom (lower side) of the VFD hemispherical base tube for (i and ii) 1000 to 3000 rpm, (iii and iv) 4000 to 6000 rpm, and (v and vi) 7000 to 8000 rpm, respectively; accuracy of relative film thickness ±50 μm, and accuracy of height of the film along the tube ±1 mm. The filled in areas highlight the uniformity of the thickness of the film of toluene over water for a specified speed. (d) Layer thickness as a function of height up the tube and rotational speed; (i) and (ii) the D_2_O layer, top and bottom of tube respectively, and (iii) and (iv) the toluene layer, top and bottom of tube respectively. (Full experimental details along with data for different mixtures of D_2_O and toluene are in the ESI, S1‡).

Mechanoenergy that is generated centrifugally and diverted into dynamic thin films has been harnessed to mediate a diversity of chemical, biochemical and material processes. Such microfluidic platforms include spinning disc and rotating tube processes (reactors),^10^ and the vortex fluidic device (VFD) which has a rapidly rotating tube tilted at 45° (*θ*) as the optimal processing angle for a myriad of applications, including in the chemical, biological and materials sciences, and it is the angle with unique fluid flow for processing homogeneous liquids in the device.^11^ Moreover, the 45° tilt angle is optimal whether the VFD is used in the confined mode for a finite amount of liquid in the tube, or under continuous flow where jet feeds deliver reagents to the base of the tube and/or at positions along the tube Fig. 1.^11^ Unique high shear topological fluid flow regimes have been identified for monophasic liquids processed in the VFD along with the development of a general fluid flow model that accounts for all the processing in the device at *θ* = 45°.^11^ The impact on high heat and mass transfer in such liquids associated with these topological fluid flows has been established using moulding strategies, with the resulting structures taking on submicron to micron dimensions, depending on the nature of the liquid, and the dimensions and rotational speed, *ω*, of the tube, typically made of quartz with a hemispherical base, 20 mm in outside diameter (OD), 17.5 mm internal diameter (ID) and 18.5 cm in length.^11^ Coriolis forces from the hemispherical base of the tube generates a ST (tornado type) topological fluid flow, with eddy currents from Faraday waves twisted by Coriolis forces generating DH topological flows, Fig. 1. Faraday waves are pressure fluctuations that induce eddies on the inside of the curved VFD tube that are aligned vertically across the film.^11^ Both topological fluid flows are present for increasing *ω*, with spicular or spherical flow arising from a combination of ST flow and DH flow of similar diameter and associated forces.^11,13^ Importantly the shear stress in the VFD is not uniform, but rather it can be uniformly localised. The above moulding strategies included so called ‘molecular drilling’, and shear stress induced polymerisation, crystallisation and aggregation.^11^ Related theoretical studies account for flow-mediated transport of molecules involved in shear stress induced aggregation of colloids,^14^ colloidal breakup,^15^ and nucleation and crystallisation of colloids.^16^ The topological fluid flows are essentially vortex rings, which are prevalent in fluids and other states^17^ with implications for biology studies.^18^

VFD processing of immiscible liquids can result in well-defined separated phases rather than emulsions,^19^ as in (i) biodiesel synthesis (three separate phases),^20,21^ (ii) protein separation in a mixture of PEG and aqueous potassium phosphate^22^ with no evidence of damage to the proteins under periodic high shear in the topological fluid flows which is in accord with the ability to use the VFD to refold proteins into their native states and to accelerate enzymatic rections,^20^ (iii) where water seemingly acts as an ‘anti-solvent’ for a toluene solution of fullerene C_60_ in forming self-assembled C_60_ nano-tubules,^23^ (iv) surface tension induced slicing of single and multiwalled carbon nano-tubes^24^ and (v) scrolling of graphene directly from graphite.^25^ Spatially controlled energy delivery in the topological fluid flows create conditions for controlling chemistry, which relates to fundamental insights into enhancing catalytic activity in general.^26^

We hypothesised that eddies associated with Faraday waves on the surface of a liquid centrifugally forced over a more-dense immiscible liquid in the VFD tube tilted at 45° can lead to DH flow which intersect both thin films of the liquid when the rotational speed is above that required for DH flow for the individual liquids in separate tubes also tilted at 45°. At the interface of the two liquids, molecules in the liquid held against the surface of the glass tube would be streaming into the other liquid and *vice versa*, beyond diffusion control limits.^11^ The resulting *in situ* generated localised high surface area under such non-equilibrium conditions can then promote high mass transfer between the immiscible liquids. In testing this hypothesis, the nature of the topological fluid flow including its dimensionality for two immiscible liquids with different densities in the VFD has been investigated using (i) direct *in situ* neutron imaging of immiscible liquids with derivable relative film thickness, (ii) time dependent mixing experiments and temperature profiling for the hemispherical base tube, and for comparison a flat base tube, (iii) ‘molecular drilling’ across a uniform layer of polysulfone inside the tube (tube-liquid interface), (iv) the structure and dimensionality of self-assembled material at the interface of the two liquids (liquid–liquid interface), and (v) the ability to promote two phase organic reactions in the absence of phase transfer catalysts. A challenge with the rotating reference frame for the spinning tube in the VFD is that the dimensions of the topological fluid flows, as established for a monophasic systems,^11^ cannot be measured directly, with distortions in the tube ∼100 times greater than the dimensions of the topological fluid flows.^11^ Related to this is the use of immiscible liquids of different densities in a vertical aligned spinning disc with the ability for mass transfer across liquids to promote chemical reactions by changes in the rotational speeds.^12^ In the VFD, the thickness of the film can be ≥150 mm with centrifugal force dominating over surface tension, in a way similar to vertically concentric rings of liquids of different density on the aforementioned spinning disc,^12^ with a topological fluid flow understanding of the behaviour of the two liquids.

## Results and discussion

### Neutron imaging determined film thickness

The relative thicknesses of two immiscible liquids as a function of rotational speed for the unique tilt angle *θ* at 45° were determined using neutron imaging for a 1 : 1 mixture of deuterated water (D_2_O) and toluene, as presented in Fig. 1. Water and toluene represent an archetypal immiscible solvent system (solubility of toluene in water is 0.52 g L^−1^ at 20 °C).^27^ Also presented in Fig. 1 is the model of the interpenetrating topological fluid flows in the immiscible liquid layers. Processing the data from the neutron imaging required taking into account the different attenuating lengths of the layers of liquid across the width of the tube (ESI S1‡). The resultant cross-sectional thickness determined in the tube is strikingly different for both liquids with the less dense toluene forming a thin film of uniform thickness along the tube, of the same thickness for both the upper (top) and lower surfaces (bottom) of the tube, Fig. 1(c), (d) and (i), (ii). This uniformity of thickness is expected in the presence of Faraday waves, with the earlier modelling of the liquid in the VFD based on a wedge shape film along the tube.^28^ The thickness of the film decreases for increasing *ω*, ∼350 mm at 3000 rpm (the lower limit for maintaining such a uniform film) to ∼160 mm at 9000 rpm (the upper limit for the VFD). In contrast, the water layer is significantly thinner at the upper surface (top) relative to the lower surface (bottom), with the film wedge shaped, becoming thinner towards the open end of the tube, except at high rotational speeds *ω*, 8000 and 9000 rpm, where the upper surface is of uniform thickness along the tube (∼150 mm thick), but not so for the lower surface (ranging from ∼280 to 400 mm), Fig. 1(c), (d) and (iii), (iv). At these high rotational speeds, the uniformity on the upper surface is indicative of a Faraday wave in this section of the water film.

Neutron imaging established that the toluene layer is behaving as if there was no water present in forming uniform films along the tube, which is further born out from mixing time experiments (see below). Moreover, the thickness of the toluene layer is close to the average film thickness previously determined for toluene by itself in the VFD (∼220 mm at 5000 rpm),^11^ which was determined from the weight of liquid in the tube for a film of liquid extending to the open end of the tube. This approach is not applicable in the present study for complex immiscible liquids. The average film thickness of water by itself in the VFD is ∼320 mm at 5000 rpm where the film is uniform in thickness along the tube, with the thickness decreasing periodically for increasing rotational speed while preserving *ω*·*P*, where *P* is the pitch of the DH, Fig. 1(a).^11^ For the mixture of water and toluene, the wedge-shaped thinning of the film for rotational speeds between ∼3000 and 7000 rpm is consistent with the absence of Faraday wave driven DH flows in the water phase for the upper (top) and lower (bottom) sections of the liquid. Interestingly, the layer of water centrifugally pinned on the surface of the tube by the toluene layer, is effectively removing the effects of any distortions in the glass tube itself.^11^ For *ω* <∼2000 rpm, the vortex in the tube is not maintained to the base of the tube, and indeed when this occurs the fluid flow behaves distinctly different, with unique optimal processing for aggregation induced emission/graphene oxide system.^29^

Similar findings were also found for a 1 : 3, 3 : 1 and 9 : 1 ratio of water to toluene with respect to the uniform toluene layer, and wedged shaped water layer, Fig. S8–S13.‡ However, only in the 1 : 3 ratio does the uniform thin film of water form in the upper (top) part of the tube, as for the 1 : 1 mixture; toluene rich mixtures have insufficient water to form such a uniform layer of water with the findings establishing the importance of the choice of ratio of the two immiscible liquids. The very similar thicknesses of the toluene and water layers in the upper (top) part of the tube is consistent with the presence of Faraday waves with their eddy currents in unison, and having a common DH flow, *i.e.*, the DH flows intersect both layers and deliver mass transfer across the interface of the two liquids. With an understanding of the effect of rotational speed on film thickness for immiscible liquids along and across the tube, we then turned to physical methods involving mixing and demixing in the liquids, and phenomena at the liquid–liquid and liquid-VFD tube surface interfaces.

### Mixing and demixing of immiscible liquids

**Fig. 2:**
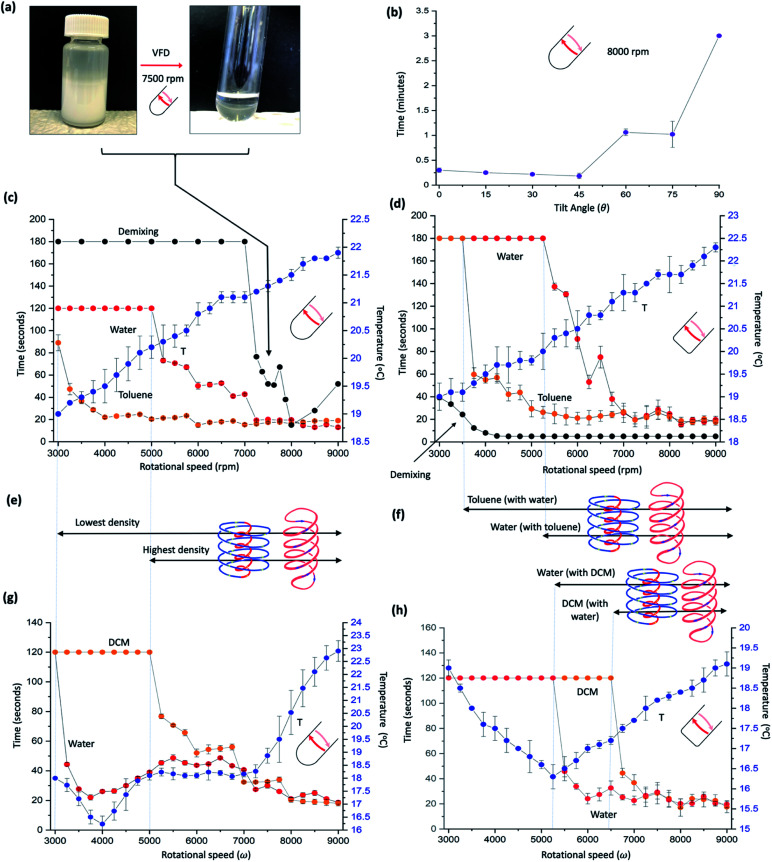
Mixing different immiscible systems. (a) Photographs of emulsified toluene and water, and the demixed liquids post VFD processing at *ω* 7500 rpm, tilt angle 45°, in a quartz tube 20 mm OD, 17.5 mm ID, 18.5 cm in length. (b) Effect of tilt angle on demixing times of toluene and water (1 : 1) in a hemispherical base at *ω* 8000 rpm. (c) Thermal response and mixing times, *versus ω* for a 1 : 1 mixture of water and toluene in a hemispherical base tube. Mixing times (red and orange for water and toluene respectively) correspond to the time taken for a drop of water containing a small amount of dye added at the bottom of the tube rotating at a specific speed to uniformly mix in half way up the preformed film, and similarly for toluene for a drop of toluene containing a small amount of dye. The temperature (blue) was measured midway along the tube using an IR camera, for residual water and toluene present in the tube, being equivalent to the continuous flow mode of operation of the VFD (water and toluene along the complete length of the tube). The demixing time (black) is the time taken for 2 mL of a preformed emulsion of toluene and water placed in the VFD to phase separate. (d) As for (c) for a 1 : 1 mixture of toluene and water using a ‘flat’ base VFD tube (20 mm OD, 17.5 mm ID, 18.5 cm in length) with a 2 mm radius of curvature where the base is fused to the side wall (*cf.* 8.75 mm radius of curvature for the hemispherical bas tube). (e) and (f) Designated dominant topological fluid flows in the two thin films in (c) and (d) respectively, for increasing *ω*, and linked to the thermal response and mixing times, *versus ω* for a 1 : 1 mixture of water and dichloromethane (DCM) in a hemispherical base tube (g) and a flat base tube (h), respectively. All data points in (b) and (c) and (g) and (h) measured in triplicates. (Experimental details and data for other immiscible solvent systems in ESI File, S2‡).

**Fig. 3:**
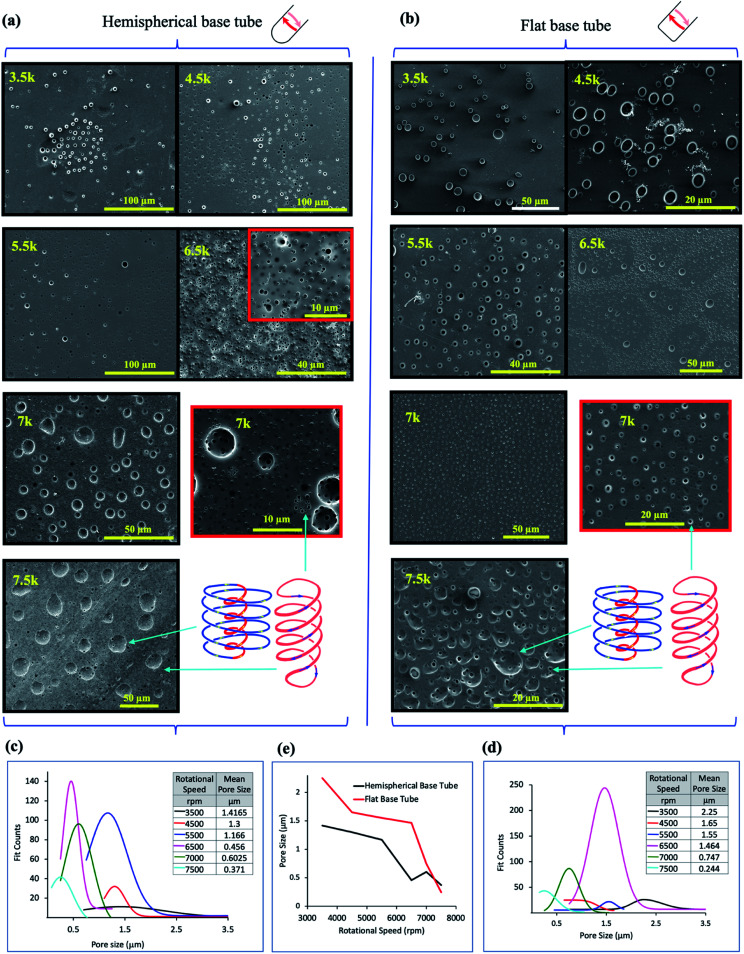
*In situ* moulding of topological fluid flow impacting on the surface of the tube. (a) and (b) SEM images of the inner surface of polysulfone films (glass–polymer interface) peeled away from the surface of the tube after processing in the VFD in a 1 : 1 mixture of toluene and water, at 20 °C, *θ* = 45°, at the rotational speeds specified, using the confined mode processing for 30 minutes, for the hemispherical and flat base tubes, respectively. For each experiment, a film of polysulfone was generated in the VFD *ca* 20 μm thick (as established using SEM of the liquid nitrogen fractured polysulfone thin films) *via* evaporation of a methylene chloride solution of the polymer (100 mg mL^−1^) in the VFD tube rotating at 6000 rpm and tilted at *θ* 5°.^11^ For the subsequent biphasic processing at *θ* 45°, the volume of the liquid in the tube was adjusted to ensure the thin film of liquid extended to the top of the tube, and after processing the liquid was removed and the film washed with hexane prior to peeling way form the surface of the tube. (c) and (d) Pore size distribution of the holes formed on the underneath surface of the film at the different rotational speeds in the hemispherical and flat base tubes respectively. (e) Plot of the mean pore size (excluding the large craters) *versus* rotational speed for both types of tube (20 mm OD, 17.5 mm ID, 18.5 cm in length). (Full synthetic details in ESI, S4‡).

Sonication of a mixture of water and toluene affords an emulsion, and in treating this emulsion in the VFD, rapid demixing occurs for rotational speeds ≥ 7000 rpm at *θ* 45°, Fig. 2(a)–(c). Determining mixing times for a 1 : 1 mixture of phase separated toluene and water for the same mixture, using the same procedure as for individual phases in the VFD^11^ in the standard hemispherical base tube, provided insight into the nature of the topological fluid flow, as did comparing the results for the two phases in a flat base tube, and where water is mixed with an immiscible liquid now of greater density, namely dichloromethane (DCM), Fig. 2(g) and (h). For toluene and water the temperature change for increasing rotational speeds *ω* is uninformative, unlike for individual liquids themselves where sudden changes in temperature occur for changes in dominant topological fluid flows.^11^ However, mixing behaviour provide topological fluid insights. This is highlighted by a drop of toluene added to the base of the tube containing some dye having similar mixing times in the toluene layer to that in the absence of a water layer below it. But on close inspection, there is now a periodic step change in the mixing time for increasing rotational speeds, in intervals of 1000 rpm, between 4000 and 8000 rpm. These periodic changes are more pronounced than for toluene by itself (Fig. S18‡) and corelate with the thinning of the uniform film of toluene suddenly changing as the pitch (*P*) of the DH flow diminishes to a threshold whereby the film thickness cannot be maintained by the Faraday wave. The diameter of the DH flow then diminishes in forming a thinner film, initially with a larger *P*, with *ω*.*P* then the same across a 1000 rpm range, before the film thins again and so on. In addition, for toluene, the onset of ST flow is ∼3000 rpm, with its presence persisting across the rotational landscape but with its diameter increasing for increasing *ω*, Fig. 2(c). In contrast, mixing a drop of water with a dye into the water layer is distinctly different to that of water by itself, with mixing times dramatically decreasing at 5000 rpm (*cf.* 3000 rpm for water by itself)^11^ with some changes closely matching Faraday wave driven reduction in film thickness, Fig. 2(e). When considered collectively, the data is consistent with the onset of DH and ST topological fluid flows in water >5000 rpm, which is further supported by determining of the diameters of DH and ST, Fig. 3. We note that the demixing time for an emulsion of toluene and water, discussed above, at 7000 rpm, coincides with where water and toluene have the same mixing times. This suggests that the DH flow is driving the demixing, where the distinction between DH flow in water and toluene is lost and where there is consequential high mass transfer, and this also coincides when the thickness of the film of water and toluene on the upper surface, Fig. 1, becomes uniform along the tube. The change in demixing time with tilt angle, Fig. 2(b), is also informative. Tilt angles ≤45° have low mixing times where DH flows are expected, whereas for *θ* >45° the mixing times dramatically increase as expected for diminishing Faraday wave effects in approaching *θ* = 90°.

For a mixture of DCM and water, mixing of water with a drop of water containing a dye into the water layer behaves as if the denser DCM liquid was absent,^11^ and the DCM behaves like water when pinned centrifugally against the surface of the tube by the toluene, Fig. 2(g). These findings are consistent with the onset of the different topological flows in the same way, Fig. 2(e), as for a mixture of toluene and water. Water and DCM do not form a stable emulsion, unlike water and toluene, and thus demixing experiments for this immiscible mixture was not possible. However, a mixture of chlorobenzene and water form an emulsion where the density of the organic phase is also greater than that of water, with demixing behaviour and mixing times similar to that of toluene and water, Fig. S15.‡ A mixture of cyclohexene and water also underwent rapid demixing, where water is now denser than that of the organic phase, Fig. S16.‡ Temperature changes for a DCM and water mixture show a drop in temperature at 4000 rpm which is close to where the ST flow in water dominates the shear stress fluid flow (∼3750 rpm), with onset of DH, as evidenced by a slight increase in mixing time in water beyond this speed, *i.e.*, where the ST flow clashes with DH flow and *vice versa*, in slowing the mixing along the tube.

We have also changed the base of the quartz tube to a flat base, albeit with a 2 mm radius of curvature necessary to fuse the side walls of the tube to the base, with the results presented in Fig. 2(d), (f), (h), and S17–S20‡ for the individual solvents in the standard 20 mm diameter tube. This base perturbs the mixing time for both toluene and water, with a dramatic reduction in the demixing rotational speed, from ∼7000 rpm for the hemispherical base tube to ∼3500 rpm. This is consistent with DH flow being common for both phases, where the distinction between the two phases is lost, and where there is high mass transfer between the two phases, in accelerating the demixing. As for the two immiscible systems in the hemispherical base tube, temperature changes are uninformative for water and toluene. However, for a mixture of DCM and water there is a dramatic reduction in temperature at ∼5250 rpm which coincides with the onset of ST and DH topological flows from the Coriolis force for the now 2 mm radius curvature of the base of the tube, *cf.* 8.75 mm radius of curvature for the hemispherical base tube. The significant reduction in temperature arises from latent heat of evaporation of highly volatile DCM (b.p. 39.6 °C), where the topological fluid flows in the water phase are breaking through the DCM phase, with the DCM evaporating at the air–liquid interface. The onset of the different topological fluid flows can be deduced from the changes in mixing times for the water/toluene and DCM/water mixtures in the VFD, Fig. 2(f). For water with DCM, the water behaves as if it was the sole liquid in the tube,^11^ whereas the behaviour of toluene by itself is distinctly different >6000 rpm relative to a mixture of toluene with water, with the mixing time fluctuating, and increasing dramatically above 8000 rpm, Fig. S18.‡ An increase in mixing time can arise from a reduction in the effect of the ST flow as the liquid is pushed up tube. Essentially removing this in the presence of water, Fig. 2(d), can arise by the water cushioning the effect of distortions in the glass tube.

The rapid demixing of an emulsion of toluene and water at much lower speed in the flat base tube, 3000 rpm (*versus* 7000 rpm in the hemispherical base tube), is noteworthy and for this a different process occurs whereby the small radius of curvature, ∼2 mm, creates a different type of high shear topological fluid flow. We also studied the effect of having two immiscible liquids that are close in density, utilising a mixture of chlorobenzene with water containing calcium chloride to increase the density near that of the organic solvent. As the density of water approached that of chlorobenzene there was no significant change to the final volume in the tube, *i.e.*, the two liquids are volume additive in the tube. However, this system is difficult to control due to the high vapour pressure and rapid evaporation changing the density of the phases in localised regions (ESI S3‡).

### *In situ* moulding of topological fluid flow impacting on the surface of the tube

The dimensionality and spatial arrangement of the topological fluid flows in a biphasic mixture of water and toluene as a 1 : 1 mixture has been established by ‘molecular drilling’ on a thin layer of polysulfone ∼20 mm thick fabricated on the inner surface of the quartz tube by evaporation of a DCM solution of the polymer for the tube rotating at 6000 rpm and tilted at 5° to ensure uniformity of the layer along the length of the tube, where the VFD is effectively operating as a rotary evaporator. Holes and craters are evident only on the inner surface of the polymer layer (sitting over the quartz tube), after carefully peeling it away for scanning electron microscopy (SEM) studies, Fig. 3. We note that for toluene by itself in the VFD, the holes drilled into the film are also on the inner surface of the film, with some solubility of the polymer in toluene effectively smoothing out the polymer film at the interface with toluene.^11^ In addition, for water only in the VFD, no molecular drilling is evident with a smooth upper and lower surface of the polymer layer unperturbed, establishing the importance of some solubility of the polymer being required to mould (*via* drilling) the shape of the topological fluid flow. For a mixture of water and toluene, where the water is centrifugally held on the surface of the tube, with water being denser than toluene, holes and craters are also formed on the inner surface of the film, Fig. 3, for both the hemispherical and flat base tubes. Their presence indicates that mass transport of toluene occurs and is thought to occur *via* Faraday wave vertical motion toward the surface. Toluene molecules are forced onto the surface of the polymer, such that both the DH and ST topological flows in toluene strike the quartz surface, as depicted in Fig. 1(a). Unlike for toluene alone in the VFD, holes (but not the craters) are often evident on the surface of the polymer in contact with the liquids, (ESI S4‡), which establishes that the water primarily covering this surface is protecting the polymer from being dissolved and recycled under the influence of the high shear topological fluid flows. The much smaller diameter of the holes *versus* the craters, effectively protects them from being smoothed over in the presence of toluene contacting the surface. Also noteworthy is that the dissolution of the polymer associated with ‘drilling’ is reflected in crystallisation of small particle of the polymer, indeed with some of them having holes drilled through them (Fig. S32‡).

SEM images for the polysulfone films following processing in a 1 : 1 mixture of toluene and water show a clear trend of increasing diameter of the ST flow for increasing *ω*, whereas the diameters become smaller for the DH flows, Fig. 3(a) and (b), in the hemispherical and flat base tubes, respectively. Unlike for toluene by itself in the VFD,^11^ there are no ordered arrays of the holes running parallel to the rotational axis of the tube; the ordered arrays correspond to the time domain arrangement of DH flows. The absence of such arrays in the biphasic mixture is consistent with the layer of toluene moving relative to the layer of water, as for vertically aligned layers of immiscible liquids of different densities in a spinning disc.^12^ This also supports the water layer effectively eliminating effects of defects in the quartz tube (see above). Analysis of the size of the holes drilled (pores) into the polymer films is shown in Fig. 3(c) and (d) for hemispherical and flat base tubes, respectively, with a comparison of the average pore size in Fig. 3(e). For the hemispherical base tube, the distinction between the diameter of the holes arising from ST and DH flows is clearly defined at high rotational speeds, 7000 and 7500 rpm, at ∼0.6 mm, and ∼0.37 mm, respectively, with the diameters of ST and DH similar at lowers speeds, where there is the formation of spherical or spicular flow.^11^ For the flat base tube, the distinction between the ST and DH occurs at a lower speed, 6500 rpm, where they are ∼1.46 mm in mean diameter, but this is lost at 7000 rpm, then re-emerging at 7500 rpm where they are ∼0.75 to 0.35 mm in mean diameter. At this rotational speed the diameter of the holes is on average smaller than that for the hemispherical base tube. As to the DH generated holes, the mean diameters for both the hemispherical and flat base tubes become progressively smaller for increasing *ω*, but with those for the latter being significantly higher until 7000 rpm where they become similar, ∼0.6 and 0.75 mm for the hemispherical and flat based tubes respectively. The similarity is consistent with the DH flow being the dominant focused high shear in the VFD, *i.e.*, where the Faraday waves dominate the shear stress. The reason for the lack of clearly defined holes for ST flow in the flat base tube at 7000 rpm, is likely due to the diameter of the ST and DH being the same, *i.e.*, where spicular flow prevails.^11^ This is at much higher *ω* than that for the hemispherical base tube, which appears to be at ∼4500 rpm, and the difference between them of ∼2500 rpm reflects the difference in curvature at the base of the tube generating the ST. This aside, the diameters of the DH flows which incorporate the same Coriolis forces associated with curvature along the tube are not overly different. Overall, the drilling experiments provide insight into the *ω* dependent dominance of ST *versus* DH flows with the drilling into the polymer film arising from the mechanical energy which is reflected in high temperature heating zones presented on the surface of the tube.^11^

### Liquid–liquid interfacial high mass transport

**Fig. 4:**
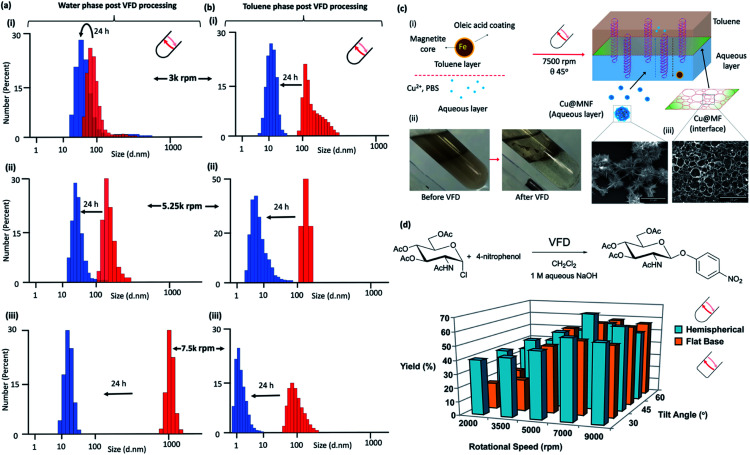
Liquid–liquid interfacial high mass transport. Dynamic light scattering (DLS) of the water phase immediately (red) after processing in confined mode in the vortex fluidic device with a 1 : 1 mixture of toluene for 15 minutes at rotational speeds, *ω*, (i) 3000 rpm, (ii) 5250 rpm and (iii) 7500 rpm, tilt inclination of *θ* +45° and 24 hours later (blue). (b) DLS of the toluene phase immediately (red) after processing in confined mode in the vortex fluidic device with a 1 : 1 mixture of water for 15 minutes at rotational speeds, *ω*, (i) 3000 rpm, (ii) 5250 rpm and (iii) 7500 rpm, tilt inclination of *θ*, +45° and 24 hours later (blue). (c) Fabrication of Cu_3_(PO_4_)_2_@Magnetite thin film (Cu@MF) and Cu_3_(PO_4_)_2_@Magnetite nanoflower (Cu@MNF) in a biphasic system of toluene and phosphate buffer saline (PBS) in a 20 mm OD, 17.5 mm ID quartz tube, 39 cm long with a hemispherical base. (i) Schematic of the formation of Cu@M composites in a VFD; oleic acid coated magnetite nanoparticles were dispersed in the toluene phase at 0.2 mg mL^−1^ with all experiments conducted using 5 mL toluene and 5 mL PBS (10 mM). The biphasic system was briefly sonicated followed by quick addition of 536 μL CuSO_4_ with then immediate VFD processing at 7500 rpm for 30 min. Cu@MF was observed at the toluene/interface whereas Cu@MNF was observed in the PBS phase, (ii), as shown in the SEM, (c). (Full characterisation details in Fig. S81‡) (d) Reaction of 2-acetamido-3,4,6-tri-*O*-acetyl-α-d-glucopyranosyl chloride with 4-nitrophenol in the presence of aqueous sodium hydroxide solution and dichloromethane, in the absence of a phase transfer catalyst, with the yield of the product plotted as a function of tilt angle *θ* and rotational speed of a quartz tube (20 mm OD, 17.5 mm ID quartz tube, 18.5 cm long) with either a hemispherical or flat base. (Full synthetic details in ESI File S6‡).

Dynamic light scattering (DLS) for processing at 3000, 5250 and 7500 rpm in a 1 : 1 mixture of toluene and water in the hemispherical base tube established the presence of nano-particulates in both phases immediately post VFD processing (∼5 min) and 24 hours later the particles present are smaller, Fig. 4(a) and (b), respectively. These nanoparticles are comprised exclusively of just water and/or toluene. For the water phase, the change in diameter of the particles observed is from ∼70 to 30 nm, ∼125 to 30 nm, and ∼1 μm to 20 nm, at 3000, 5250 and 75 000 rpm, respectively, with the corresponding changes in the toluene phase at ∼120 to 12 nm, ∼125 to 8 nm and ∼80 to 2 nm respectively. High mass transfer between the two phases is therefore prevalent in the VFD at 45° tilt angle, with the biggest particles formed in water at 7500 rpm, which corresponds to the rotational speed where emulsions of the two phases rapidly demix, and where the evidence supports that the DH flow is common to both phases, as detailed above. In contrast, DLS data for the water phase processed in the flat base tube reveal that they all have particles close to 80 nm in diameter immediately following VFD processing. They change to ∼300 nm after 24 hours processing at 3000 rpm, and for the other two speeds, the particles are essentially unchanged over the same period (ESI S5‡). This is consistent with the uniqueness of the fluid flow at *ω* 7500 rpm in the hemispherical base tube. For the toluene phase in the flat base tube, the initially formed particles formed at *ω* 3000 and 5250 rpm are ∼8 and 1 μm respectively, changing now to larger particles after 24 hours, to ∼50 and 9 μm, whereas for *ω* 7500 rpm the particles are unchanged at ∼150 nm. The size of the nanoparticles comprised of one or two phases in one phase and *vice versa* depends on the shape of the base of the tube, and thus where different topological fluid flows with different shear stress are generated.

The impact of DH flow generated at 7500 rpm at the interface of a 1 : 1 mixture of an aqueous phase and toluene was explored using the self-assembly of inorganic nanoparticles. Here the aqueous phase was phosphate buffer saline or PBS with Cu^2+^ and toluene phase was a colloidal suspension of oleic acid coated magnetite nanoparticles, processed at *ω* 7500 rpm (*θ* 45°), Fig. 4(c) and (i). VFD processing resulted in phase demixing, Fig. 4(c) and (ii), with a film of Cu_3_(PO_4_)_2_@Magnetite (Cu@MF) formed at the interface of the two liquids showing a pattern of holes ∼2 to 4 μm in diameter which is the dimensionality for DH flow, *i.e.*, the holes represent a region where the DH was common to both phases at the liquid–liquid interface, with the assembled material representing a mould of the outer peripheral as such in the plane parallel to the surface of the tube, with nanoflowers of Cu_3_(PO_4_)_2_@Magnetite (Cu@MNF) formed in the PBS phase, Fig. 4(c) and (iii). In comparison, benchtop vortexing of the same mixture did not generate a layer of Cu_3_(PO_4_)_2_@Magnetite, and the flat base tube did not form a structured interface, Fig. S81.‡ The latter is understood by the smaller dimensionality of the ST being similar to that of the DH flow, Fig. 3(b), disrupting the self-assembly for the DH flow at the interface. Both Cu@MF and Cu@MNF formed as a result of induced mass transfer of oleic acid coated magnetite striking the phase boundary driven by the DH flow in the VFD. Magnetite particles dispersed in the toluene phase were trapped through the van der Waals interaction-induced physisorption into the Cu_3_(PO_4_)_2_ petal sites^30^ and finally became stabilised in the PBS phase. To prove this, elemental EDX maps on O, Fe and Cu overlap over both structures which was further confirmed by FTIR and XRD (Fig. S82‡). FTIR analysis on Cu@MF revealed peaks at 1044 and 990 cm^−1^ corresponding to phosphate P–O vibrations^31^ and 2977 and 2894 cm^−1^ corresponding to C–H vibrations and the peak at 1254 cm^−1^ to the C–O vibration.^32^ XRD patterns show that the Cu@MNF fits well with well-known magnetite and Cu_3_(PO_4_)_2_ crystallinities from the crystallography database (00-001-0054) and magnetite (00-001-1111) (Fig. S82‡).

The effect of the choice of *w* and shape of the base of the tube on reactions that would otherwise require a phase transfer catalyst were also studied, along with the effect of changing the tilt angle of the tubes, 15° either side of that optimal 45° for applications in the hemispherical base tube was studied, Fig. 4(d). This was for the reaction of 2-acetamido-3,4,6-tri-*O*-acetyl-α-d-glucopyranosyl chloride in dichloromethane with sodium 4-nitrophenoxide generated *in situ* in water from 4-nitrophenol and sodium hydroxide solution, as an immiscible two-phase system in a 1 : 1 ratio with the denser DCM centrifugally held on the surface of the quartz tube with water above extending to the air–liquid interface. This reaction was studied in the absence of a phase transfer catalyst in the hemispherical base tube, and for comparison the flat base tube, Fig. 4(d), with full synthetic details in the ESI S6.‡ The hemispherical base tube has the highest yield at *ω* 7000 rpm for a 45° tilt angle, with yields for the flat base tube being significantly less although it is nevertheless optimal at *θ* 45°. We note that *ω* 7000 rpm for the hemispherical base tube corresponds to the speed where the mixing times for water and DCM become the same for increasing *ω*, Fig. 2(g), such that the signature of the mixture, Fig. 2(g), matches the expected optimal rotational speed. The finding is also consistent with *ω* 7000 rpm corresponding to the commonality of the expected DH flow at the interface of the two liquids, *i.e.*, where there is the highest mass transfer, thereby circumventing the need for a phase transfer catalyst. The control experiment of mixing the same solutions together using batch processing yielded only traces of the product. Also noteworthy is that the yield using the flat base tube is significantly higher at lower rotational speeds. In this context, phase demixing of an emulsion of toluene and water occurs at much lower speeds in the flat base tube (water and DCM do not form a stable emulsion for comparative studies). Translating this finding to the water/DCM mixture in the flat base tube would predict higher mass transfer between the two phases at lower speeds relative to the hemispherical base tube, and this is indeed the case. Thus, we have a model for interpreting the signature of a mixture of immiscible liquids (mixing times, demixing times if available, and change in temperature for increasing *ω*), for predicting the ability to accelerate reactions without the need for a phase transfer catalyst. This is understood by a dominance of DH high mass transfer topological fluid flow, with the process adhering to the tenets of green chemistry in avoiding the use of an auxiliary substance. We also note that reactions occurring without the need for a phase transfer catalyst relate to a possible explanation of ‘on water’ effects,^33–35^ for now where there is mixing occurring between immiscible liquids at the submicron dimension scales.

## Conclusions

Unique insights into understanding the nature of the high shear topological flow in thin films of two immiscible liquids in the VFD at the optimal beneficial tilt angle of 45° have been achieved in this work. These have occurred through integrating a number of different investigative strategies for overcoming the challenges associated with having a rotating reference frame and the small dimensionality of the high shear topological flows in liquid films ≥∼150 mm thick. The rotational speed dependent strategies have included the combination of (i) neutron imaging film thickness studies, (ii) mixing experiments within each of the immiscible liquids along with temperature changes, and (iii) structural perturbation of polymer films on the surface of the tube and nanomaterials assembled at the interface of immiscible liquids. This understanding provides a level of predictability for the optimal rotational speed *ω* within the VFD for a tilt angle *θ* of 45°, as highlighted for carbohydrate transformations in an immiscible mixture of water and dichloromethane, Fig. 4(d). The interfacial tension between the two liquids, and immiscible liquids in general, is transitioned in the VFD which provides the energy cost for high mass transfer of two liquids into each other, with DLS revealing the particulates of each phase within the other. Remarkably the use of the VFD in this way does not result in the formation of emulsions, despite high mass transfer being maintained between the two liquids. The formation of nanoparticles within each phase for a biphasic mixture during VFD processing is consistent with high surface area of the two liquids relative to each other. Interestingly, combining toluene and water is a well-studied archetypal immiscible biphasic system with toluene having almost no effect on the hydrate phase equilibrium boundary of the methane–water system.^36^ At the air–liquid interface in the VFD, there is the expected increase in mass transfer of gases into the water phase, and indeed for both phases of immiscible liquids in the VFD. In this context, for the water layer, dissolved gases can limit the solubility of oil^37^ (*cf.* toluene in the present study) and how this is manifested under shear is yet to be realised. Also noteworthy is the Faraday wave driven uniformity of the toluene and other less dense liquids in biphasic mixtures in the VFD. This relates to levitating a liquid, where excitation resonance of the supporting air layer prevails.^38^

Pre-formed emulsions of water and toluene in the VFD can be rapidly demixed at an increasing rotational speed threshold, being consistent with the general model of topological fluid flow presented in Fig. 1(b), notably (i) high shear ST topological fluid arising from Coriolis forces from the base of the tube, in a way depending on its shape, either for a hemispherical base or a flat base with a small radius of curvature of the tube where the base is fused to its side walls, and (ii) DH flow arising from a combination of Faraday wave eddy currents and Coriolis forces. The different dimensionalities and *w* dependent onset of different topological fluid flows bodes well for changing the shape of the base beyond those studied herein for added control in certain applications, and how the topological fluid flow changes with change in diameter of the tube. The formation of nanoparticles in the phase separated layers, as unstable nano-emulsions (decomposing over 24 hours), is from the high mass transfer associated with the micron/sub-micron sized topological fluid flows at the interface between the immiscible layers, Fig. 1. The use of the VFD in this regard simplifies processing with immiscible liquids, in avoiding the need for phase transfer catalysts to drive chemical reactions where there is preferential take up in the different layers for different reacting substrates. The combined understanding of fluid flow with changing the shape of the base of the tube opens new opportunities for VFD processing, for harnessing mechanical energy generated with topological fluid flow of differently focused energy, and in dealing with challenges associated with using highly viscous liquids. Interestingly the non-equilibrium spatially arranged DH fluid flow regimes in the toluene layer in the VFD impacting on the water layer though to the surface of the tube are laterally moving relative to the surface of the tube such that the molecular drilling results in a random array of holes, Fig. 3, unlike for a mono-phasic system of toluene alone on the VFD.^11^ This effectively creates a more uniform non-equilibrium conditions at the interface of the tube for a binary mixture of liquids. In addition, perforating the layer of polysulfone with holes has potential for fabricating material for used as a size-exclusion filter or dialysis-like membrane to separate varying diameter particulates.

## Data availability

All the experimental conditions and data supporting this article have been included in the main text and figure captions and in the ESI.‡

## Author contributions

M. J., C. C. and Z. G. carried out thermal imaging and mixing experiments, E. M. C., S. J. P, N. J. and F. S. carried out the carried neutron imaging experiments, D. B. J. analysed the neutron imaging experiments and interpreted the data, M. J. and S. H. carried out the DLS experiments, X. L. carried out the nanomaterial synthesis at the interface between the liquids, K. A. S. developed the organic synthesis, J. M. C., W. Z., Y. T., J. S. Q., D. M., X. C. and K. V., contributed to the development of the experiments, D. H. carried out experiments of immiscible liquids of the same density, and C. L. R. designed the VFD microfluidic platform, coordinated the research and developed the model for the fluid behaviour. All authors contributed to writing and editing the manuscript.

## Conflicts of interest

There are no conflicts to declare.

## Supplementary Material

SC-013-D1SC05829K-s001

## References

[cit1] Demurtas D. (2015). et al.. Nat. Commun..

[cit2] Paria S., Hatanaka Q., Maruoka K. (2019). ACS Catal..

[cit3] Gumerov R. A. (2016). et al.. ACS Macro Lett..

[cit4] Rumyantsev A. M., Gumerov R. A., Potemkin P. I. (2016). Soft Matter.

[cit5] Wiese S., Spiess A. C., Richtering W. (2013). Angew. Chem. Int. Ed..

[cit6] Sheth T., Seshadri S., Prileszky T., Helgeson M. E. (2020). Nat. Rev. Mater..

[cit7] Ito Y. J. (2005). J. Chromatogr. A.

[cit8] Wuethrich A. (2018). et al.. Nanoscale.

[cit9] Chapman M. R. (2017). et al.. Org. Process Res. Dev..

[cit10] Chen X., Smith N. M., Iyer K. S., Raston C. L. (2014). Chem. Soc. Rev.

[cit11] Alharbi T. M. D. (2021). et al.. Nanoscale Adv..

[cit12] Cybulski O. (2020). et al.. Nature.

[cit13] Jellicoe M. (2021). et al.. Chem. Commun..

[cit14] Zaccone A. (2009). et al.. Phys. Rev. E.

[cit15] Conchuir B. O., Zaccone A. (2013). Phys. Rev. E.

[cit16] Mura F., Zaccone A. (2016). Phys. Rev. E.

[cit17] Donnelly C. (2021). et al.. Nat. Phys..

[cit18] Kilner P. J. (2000). et al.. Nature.

[cit19] Pye S. J., Dalgarno S. J., Chalker J. M., Raston C. L. (2018). Green Chem..

[cit20] Britton J., Stubbs K. A., Weiss G. A., Raston C. L. (2017). Chem. Eur. J.

[cit21] Britton J., Raston C. L. (2014). RSC Adv..

[cit22] Luo X., Smith P., Raston C. L., Zhang W. (2016). ACS Sustainable Chem. Eng..

[cit23] Vimalanathan K. (2017). et al.. Angew. Chem., Int. Ed..

[cit24] Alharbi T. M. D., Li Q., Raston C. L. (2021). ACS Sustainable Chem. Eng.

[cit25] Vimalanathan K. (2019). et al.. Nanoscale Adv..

[cit26] Chen R., Neri S., Prins L. J. (2020). Nat. Nanotechnol..

[cit27] Neely B. J., Wagner J., Robinson R. L., Gasen K. A. M. (2008). J. Chem. Eng. Data.

[cit28] Solheim T. E., Salvemini F., Dalziel S. B., Raston C. L. (2019). Sci. Rep..

[cit29] Tavakoli J., Joseph N., Chuah C., Raston C. L., Tang Y. (2020). Mater. Chem. Front..

[cit30] Luo X. (2018). et al.. ACS Appl. Mater. Interfaces.

[cit31] Yu Y., Fei X., Tian J., Xu L., Wang X., Wang Y Y. (2015). Colloids Surf., B.

[cit32] Liang J., Li H., Yan J., Hou W. (2014). Energy and Fuels.

[cit33] Mellouli S., Bousekkine L., Theberge A. B., Huck W. T. S. (2012). Angew. Chem., Int. Ed..

[cit34] Song C. E. (2019). et al.. Nat. Commun..

[cit35] Oksdath-Mansilla G., Kucera R. L., Chalker J. M., Raston C. L. (2021). Chem. Commun..

[cit36] Mohammadi A. H., Belandria V., Richon D. (2009). Ind. Eng. Chem. Res..

[cit37] Pashley R. M., Rzechowicz M., Pashley L. R., Francis M. J. (2005). J. Phys. Chem. B.

[cit38] Apffel B., Novkoski F., Eddi A., Fort E. (2020). Nature.

