# Supplementary material for: Vortex fluidic induced mass transfer across immiscible phases
Source: Chem Sci. 2022 Jan 31;13(12):3375–85. doi: 10.1039/d1sc05829k (PMC8943860; doi:10.1039/d1sc05829k)
Supplement: SC-013-D1SC05829K-s001 [file SC-013-D1SC05829K-s001.pdf]

Electronic Supplementary Material (ESI) for Chemical Science.
This journal is © The Royal Society of Chemistry 2022

SUPPLEMENTARY INFORMATION

Vortex fluidic induced mass transfer across immiscible phases

Matt Jellicoe,[a] Aghil Igder,[a] Clarence Chuah,[a] Darryl B. Jones,[a] Xuan Luo,[a] Keith A. Stubbs,[b] Emily M. Crawley,[a] Scott J. Pye,[a] Nikita Joseph,[a] Kasturi Vimalananthan,[a] Zoe Gardner,[a] David P. Harvey,[a] Xianjue Chen,[c] Filomena Salvemini,[4d] Shan He,[a,e] Wei Zhang,[f] Justin M. Chalker,[a] Jamie S. Quinton,[a,g] Youhong Tang,[a] and Colin L. Raston[*a]

a. Flinders Institute for Nanoscale Science and Technology, College of Science and Engineering, Flinders University, Bedford Park SA 5042, Australia

colin.raston@flinders.edu.au

b. School of Molecular Sciences, The University of Western Australia, 35 Stirling Highway, Crawley, WA 6009, Australia

c. School of Environmental and Life Sciences, The University of Newcastle, Callaghan, New South Wales 2308, Australia

d. Australian Nuclear Science and Technology Organization, New Illawara Road, Lucas Heights, NSW, Australia

e. Department of Food Science and Engineering, School of Chemistry Chemical Engineering, Guangzhou University, Guangzhou 510006, China

f. Centre for Marine Bioproducts Development, College of Medicine and Public Health, Flinders University, Adelaide, SA 5042, Australia

g. Flinders Microscopy and Microanalysis (FMMA), College of Science and Engineering, Flinders University, GPO Box 2100, Adelaide, South Australia 5001, Australia

Contents

S1. Neutron imaging determined film thickness 3

S2. Mixing and demixing of immiscible liquids 18

S3. Equal density immiscible liquids 25

S4. In situ moulding of topological fluid flow impacting on the surface of the tube 28

S5. Liquid-liquid interfacial high mass transport - Dynamic Light Scattering (DLS) 43

S6. Interfacial self assembly of nanoparticles 67

S6. Biphasic synthesis

S7. References 72

S1. Neutron imaging determined film thickness

General Method

An example ANSTO Neutron images is shown in Figure S1 (a). The image is calibrated by first obtaining the pixel to distance ratio. Here we take a cross sectional profile of the test tube from the image [Figure S1 (c)], as the test tube has a known outer diameter of 20 mm. This also provides the internal dimension of the VFD tube, measured to be 16770 μm. Structural features are then identified from an alternative cross-sectional profile [Figure S1 (b)]. The height up the tube, with reference to the internal tube base, is then approximated based on the VFD design, schematically illustrated in Figure S1 (d). Film profiles of the liquid are then obtained at various heights up from the tube base by averaging the neutrons from regions illustrated in Figure S1 (a), with the film profiles observing a cross sectional segment of the VFD tube, orthogonal to the rotational axis, at a specific height.

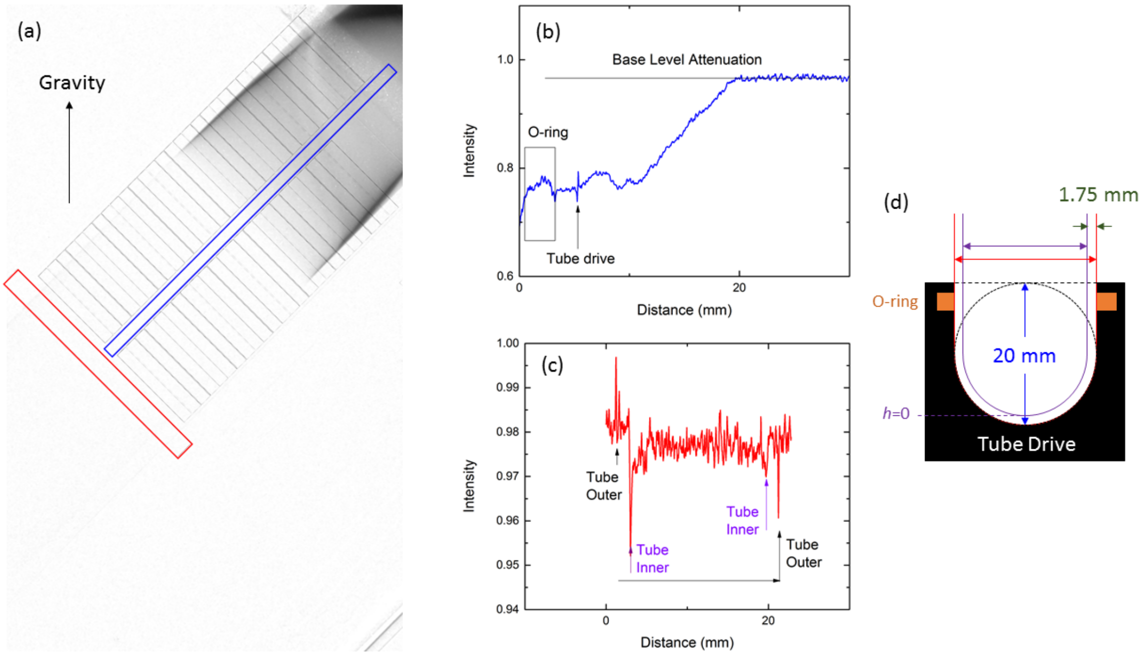

Figure S1. (a) Neutron image obtained at ANSTO. (b) Average Intensity profile obtained from blue highlighted section in part (a). (c) Average Intensity profile obtained from red highlighted section in part (a). (d) Schematic representation of the VFD tube highlighting key features used in calibrating neutron image.

In order to analyse the neutron imaging data, we have developed a neutron attenuation model to describe the neutron beam passing through the fluid layers of the biphasic system. Looking at a cross sectional segment of the VFD tube in the plane whose normal is parallel to the axis

of rotation, we assume that we have 2 circular interfaces at the fluid-fluid and fluid-air boundaries. Based on a Beer-Lambert attenuation description, that neglects refraction and reflections at the interfaces, the intensity of the neutron beam transmitted, $I(x)$ through the tube can be described through

$$I(x) = I_0 \exp(-\sigma_1 l_1) \exp(-\sigma_2 l_2) .$$

Here I_0 is the incident neutron beam intensity, σ_1 and σ_2 are the neutron probabilities per unit length from layers 1 and 2 respectively. Lastly, l_1 and l_2 are the distances that the neutron beam traverses in layers 1 and 2, respectively.

In the model, the attenuating lengths for layers 1 and 2, at a cross sectional position, x, referenced to the tube axis (positive values reflect the tube top with respect to gravity) can be obtained if the radius and centres of the circular interfaces are known, see Figure S2.

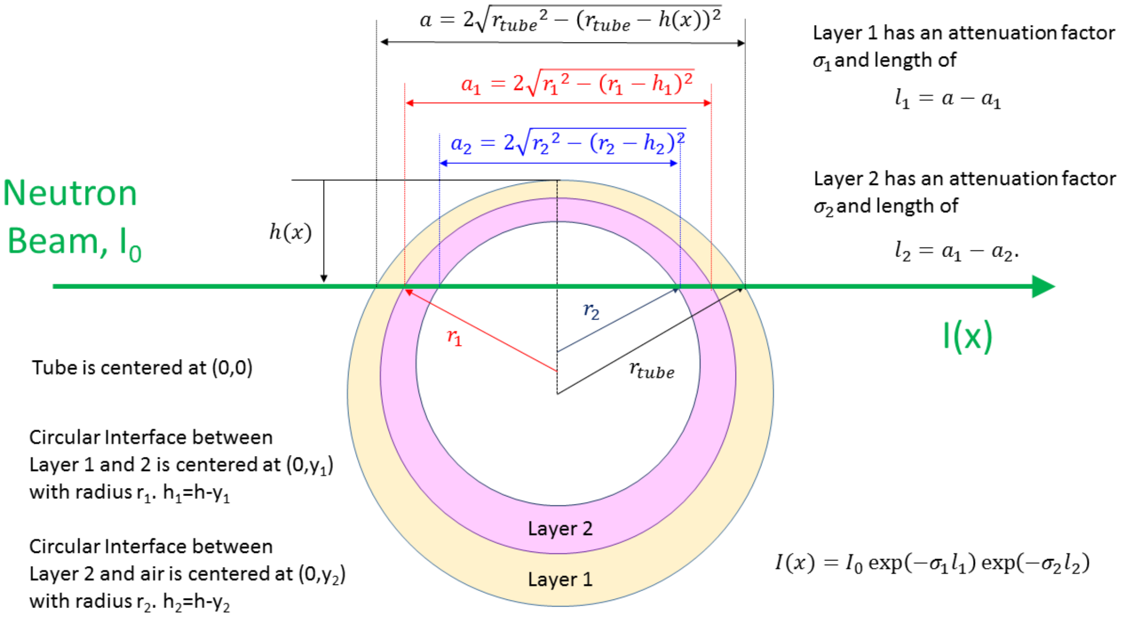

Figure S2: A Schematic representation of the neutron beam attenuation is the experiment. See text for further details.

To test that this model is appropriate, we have synthesised the attenuation of a biphasic system, see Figure S3. This approach appears to describe all of the key features observed in the cross-section profiles observed in the neutron experiment. However, fitting the attenuation profile with this functional form to extract properties about the film proved problematic. Specifically, it is necessary to convolve this attenuation properties with the instrumental response function, including additional processes such as neutron refraction and reflections at the interfaces. These

refraction/reflective characteristics of the film change with variations in film thickness, and is further complicated by the possibility of the neutron beam refracting through up to six different interfaces when transmitted through the biphasic system. This is complicated by the changing angle of incidence on the film across the diameter of the tube. We have found it impracticable to fit, the vast amount of film attenuations profiles obtained at varying heights and rotational speeds, as the continually changing instrumental response function makes the non-linear fitting procedures unstable across the cross-sectional profiles extracted from a single image at a single rotational speed.

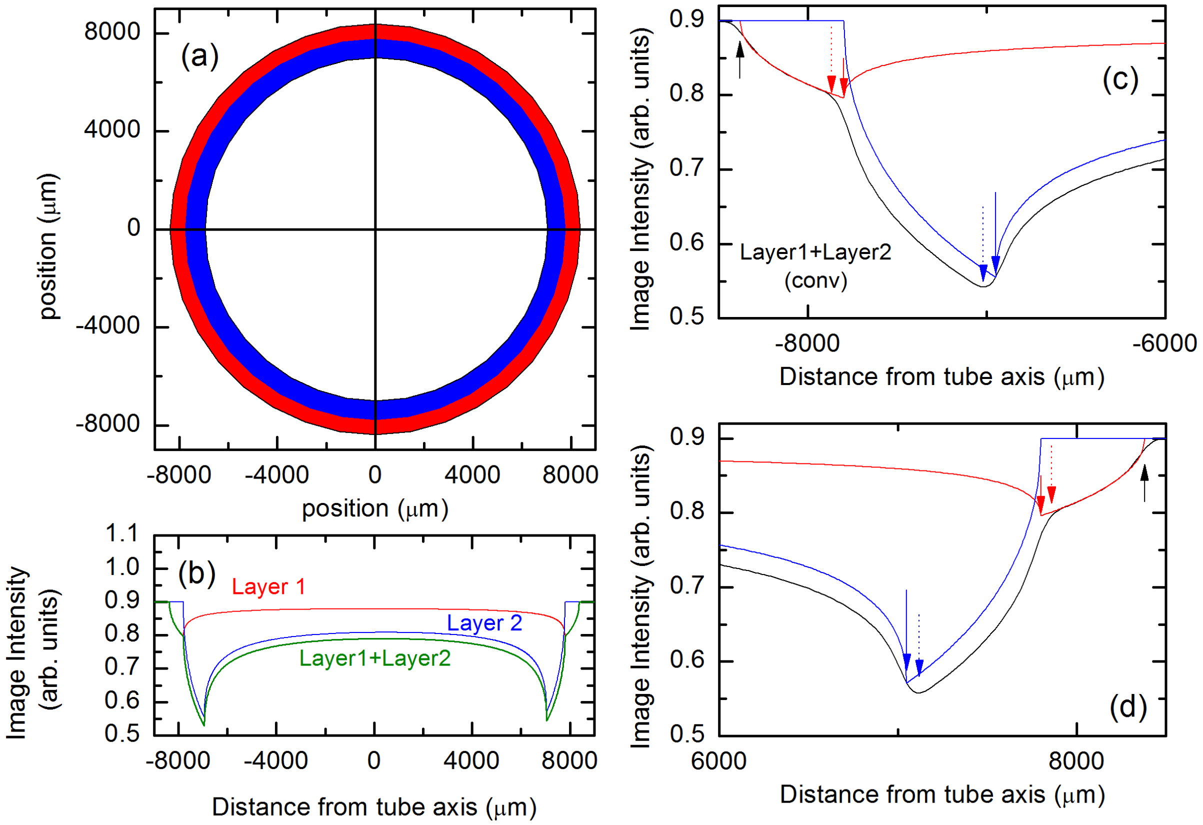

Figure S3: (a) A hypothetical biphasic system, with tube radius r_T=8380 μm, layer 1 (red) had interface radius r_l=7800 μm and is centred at (0,0), and Layer 2 (Blue) has an interface radius r_{L2} =7000 μm and is centred at (50,0). (b) The synthesized attenuation profile from layer 1 (σ_1 =1.8x10^{-5} μm^{-1}), layer 2 (σ_2 =6.0x10^{-5} μm^{-1}) and layer 1+2. Here the unattenuated intensity is set at 0.9 arb. units (c,d) Fitting of the Gaussian convoluted attenuation profile at the (c) bottom and (d) top of the tube. Also shown are the unconvoluted attenuation profiles from the two layers. Here solid (dashed) arrows show the true (derived) parameters for the layer 1 and 2 interface radii. Black arrow indicates the tube radius.

In order to extract data from the observed neutron images, we relate structural features in our cross-sectional profile to the physical parameters in the biphasic system. These features are indicated in Figure S4. Firstly, we locate the maximum attenuation (minima in profile), m and use these values to determine the radius of the inner (layer 2) interface:

$$r_{L2} = \frac{m_+ - m_-}{2}$$

This circle describing this interface is positioned with centre:

$$c_{L2} = -\frac{m_+ + m_-}{2}.$$

Here the subscript (+/-) references fitting of either the top or bottom of the tube, respectively. We then obtain the difference between the attenuated curve, and a straight line running to the baseline intensity at the tube boundary. This difference curve (slope) approximates the derivative of the attenuation curve, with its maximum value (or local maximum) or inflection point reflecting the change in attenuation properties occurring from the change in neutron attenuation between different layers.

The maxima of the difference (slope), s are used to determine the radius of the outer (layer 1) interface,

$$r_{L1} = \frac{s_+ - s_-}{2}$$

This circular interface has its centre,

$$c_{L1} = -\frac{s_+ + s_-}{2}.$$

When a second layer is not present, the s value is set to either the tube radius or outer interface. Here the subscript (+/-) again refer to either the top or bottom of the tube, respectively.

This distance of layer 1 interface from the top/bottom tube wall (Layer 1 thickness) is then

$$L_{1+} = r_T - (r_{L1} - c_{L1}),$$

or,

$$L_{1-} = r_T - (r_{L1} + c_{L1}),$$

respectively. This distance from the first to second interfaces (Layer 2 thickness) at the top/bottom of the tube is then

$$L_{2+} = r_{L1} - (r_{L2} - (c_{L2} - c_{L1})),$$

or,

$$L_{2-} = r_{L1} - (r_{L2} + (c_{L2} - c_{L1})),$$

respectively.

This distance from of the second interface at the top/bottom of the tube from the tube wall is then

$$L_{+} = L_{1+} + L_{2+}$$

or

$$L_{-} = L_{1-} + L_{2-}$$

Note that these quantities must satisfy the conservation relationship,

$$2r_{Tube} = L_{1+} + L_{2+} + 2r_{L2} + L_{2-} + L_{1-}.$$

We test that these parameters relate to the film properties by analysing our synthetic attenuation profile, convoluted with a Gaussian instrumental function (std dev = 100 μm), see Figure S3(c,d). Here we found that all of our derived parameters agreed to our synthetic parameters to within the standard deviation used to convolute the distribution. These parameters have therefore been deemed acceptable to derive film thickness information from the neutron beam attenuation profiles in the absence of being able to reasonably fit the attenuation curve. We also note that for very thin film (<200 μm), the refraction/reflections can place the inflection point on our outside of the tube boundary. In these cases, we correct the thin film thickness value to be half of the distance between the inflection point, and the distance required for the attenuation intensity to return to its base level. It is estimated that the relative film thickness accuracy is of +/- 50 μm. Here the systematic neutron instrumental response may lead to larger uncertainties on the absolute film thickness measurement. Lastly, we estimate that the height is accurate to +/- 1 mm.

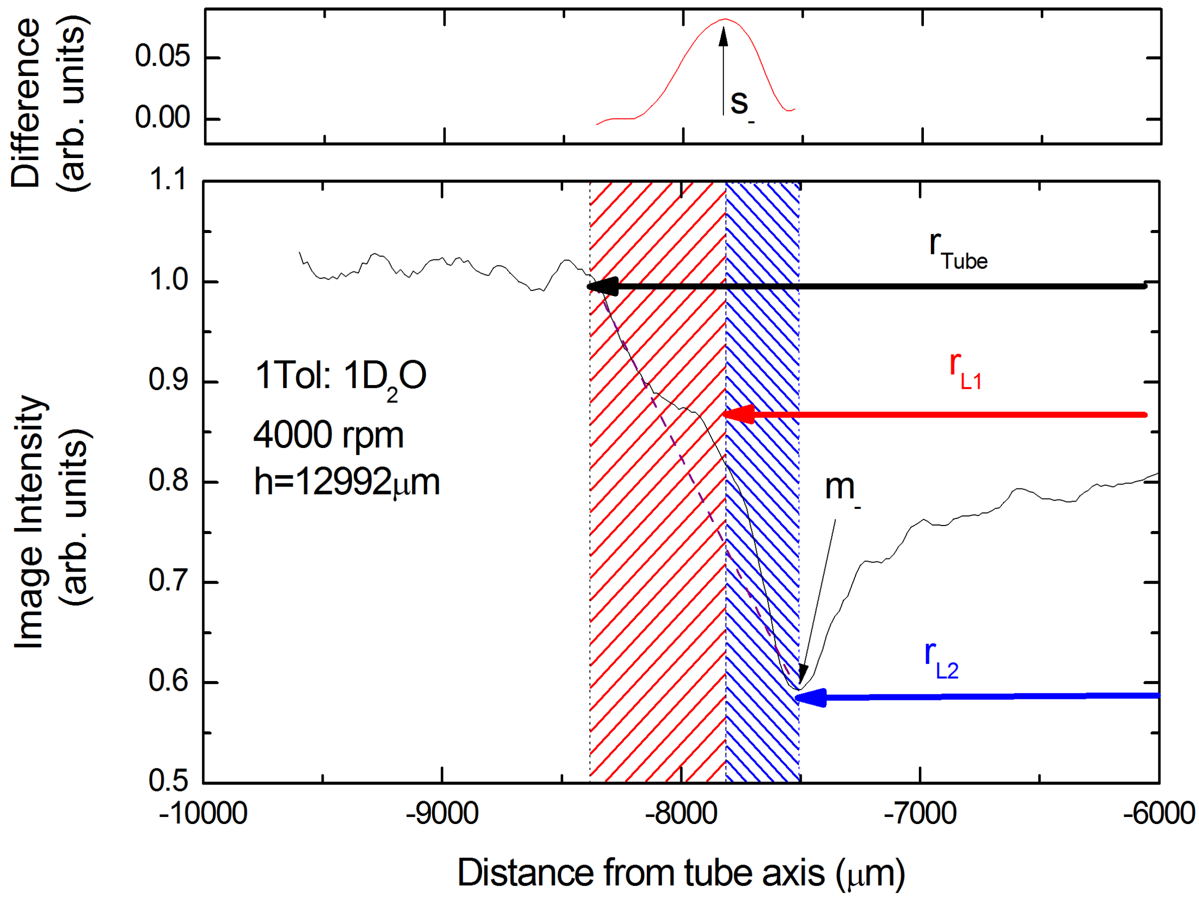

Figure S4: An illustration of how parameters of the film cross sectional profile at the bottom of the tube are interpreted in terms of the film characteristics. See text for details.

The analysis procedure works well, and has been used to extract data regarding the film thickness. We did experience some issues with the fitting procedure, in the transition regime, when Layer 1 is disappearing. Here the microfluidic fluid volume may become unstable to support a single phase with stable thickness. Here the space occupied by the fluid may be shared between the layers, or two phases may mix. In the time-averaged image, this manifests as the higher attenuating toluene layer having a blurred boundary, and a seeping of the attenuating intensity across the interface. This is shown in Figure S5.

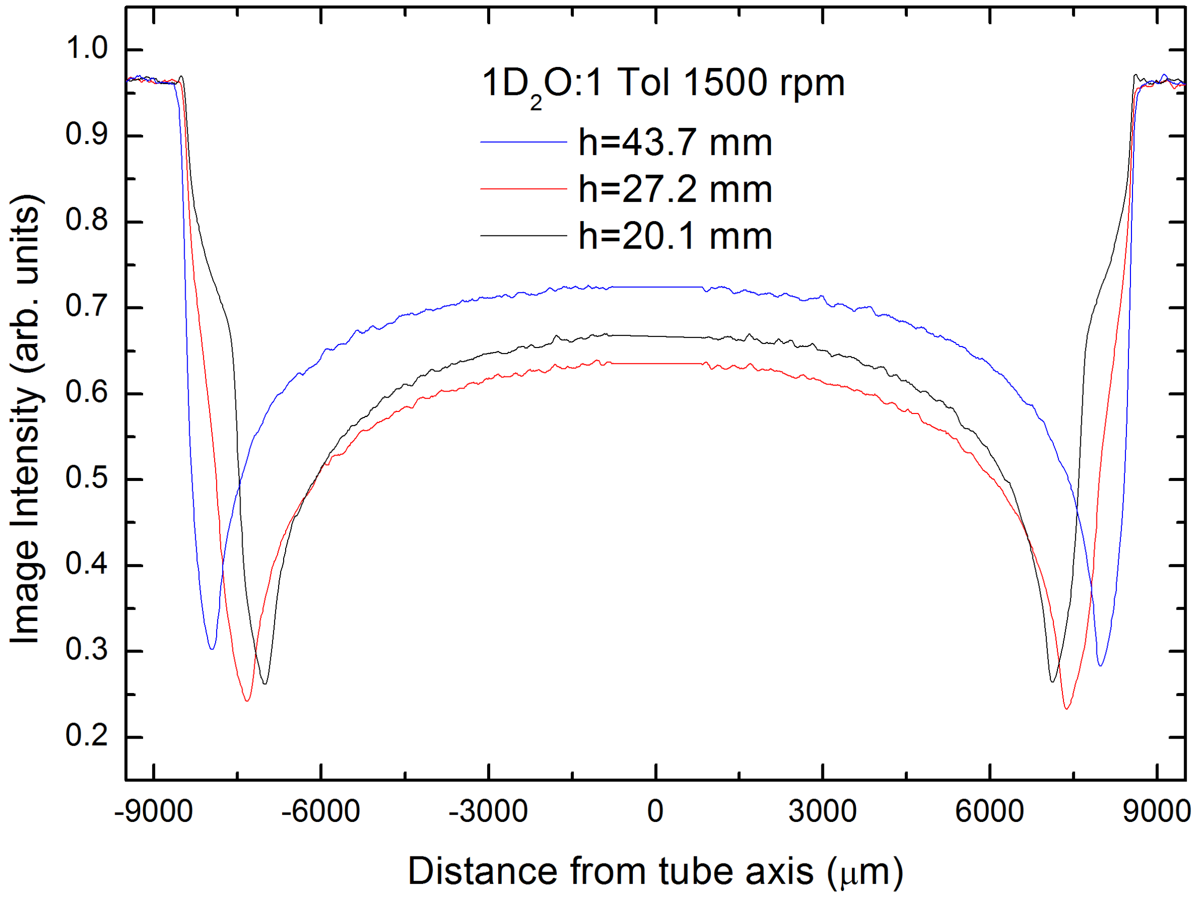

Figure S5: Film profiles at different heights where we observe a change in the phasic structure of the film. In the lower section of the tube (h=20.1 mm, black) we have two distinct phases. In the top section (h=43.7 mm, blue) we only have the toluene phase. In the mixing region (h=27.2mm, red), the distinction between the two phases becomes blurred. Note that the attenuation at the tube edge is not as strong in this mixing region as that observed for a single toluene phase, so the toluene phase cannot have the same density (concentration) on the tube edge.

Results of Film Characterisation

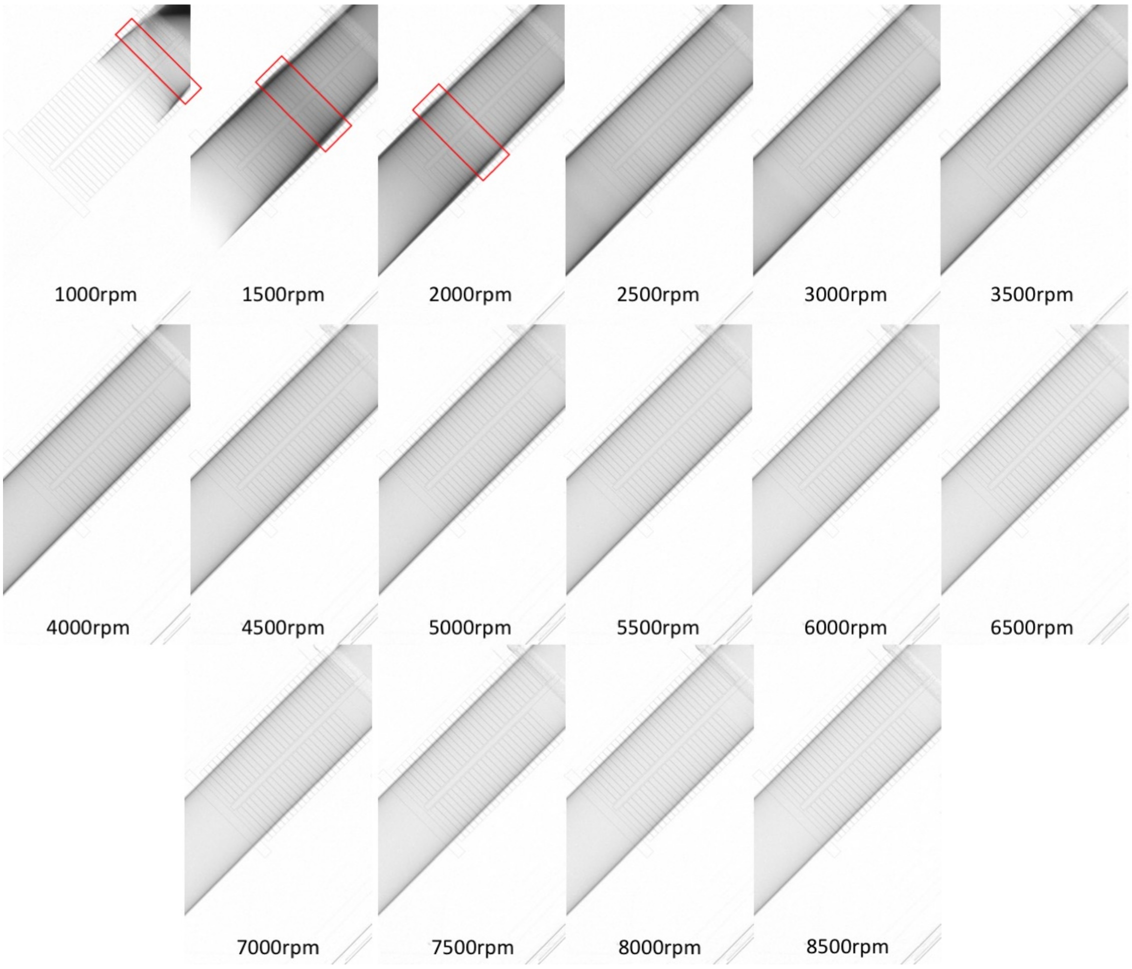

Figure 6: Neutron Images at different rotational speeds for a 1D_2O:1Toluene mixture. Red boxes indicate the regions where the D_2O/Toluene interface is blurred. Black boxes indicate the regions where attenuation cross sectional data is analysed.

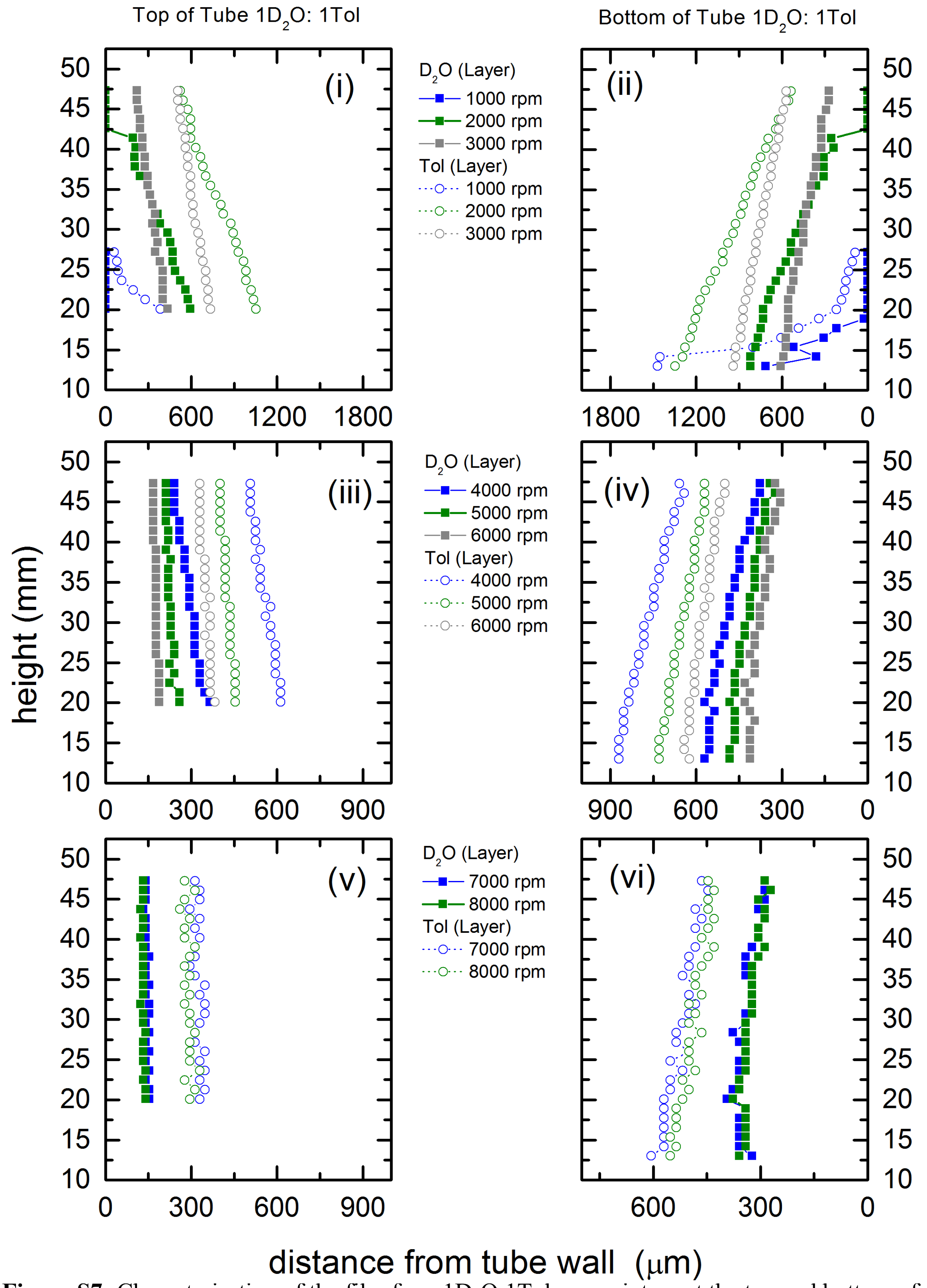

Figure S7: Characterisation of the film for a 1D$_2$O:1Toluene mixture at the top and bottom of the VFD tube for (a,b) 1000-4000 rpm, (c,d) 4000-7000 rpm, and (e,f) 7000-9000 rpm, respectively. See text for further details.

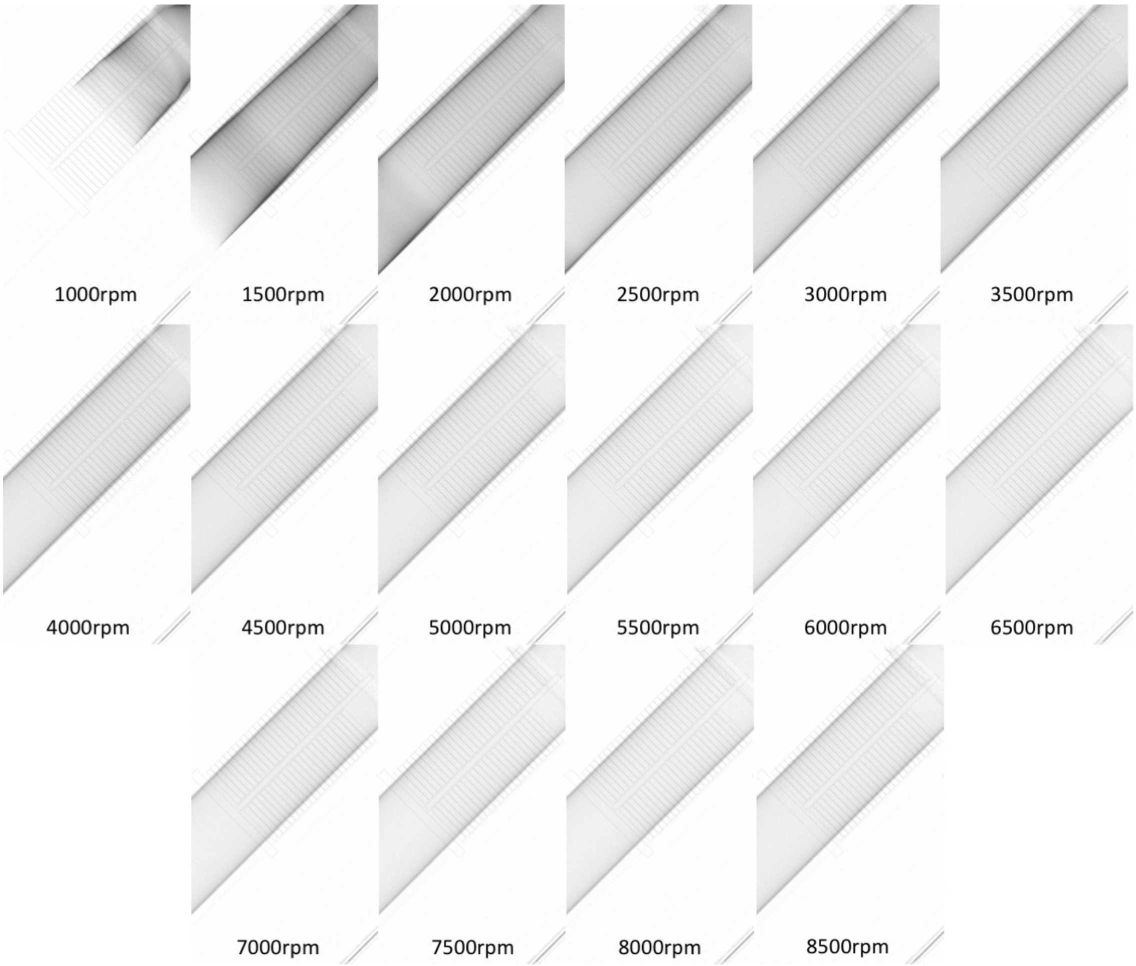

Figure S8: Neutron Images at different rotational speeds for a $3D_2O$:1Toluene mixture. Black boxes indicate the regions where attenuation cross sectional data is analysed.

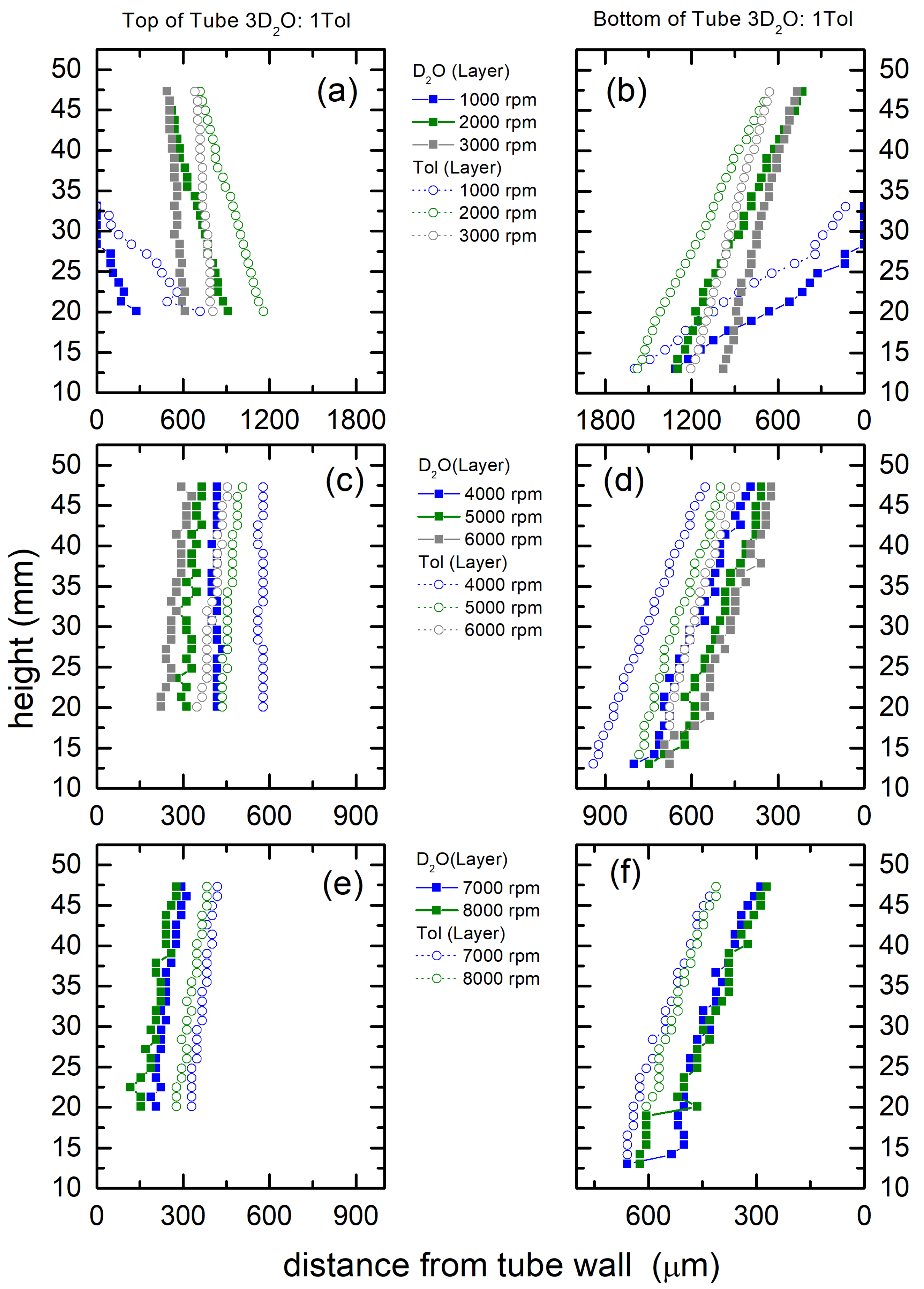

Figure S9: Characterisation of the film for a $3D_2O$:1Toluene mixture at the top and bottom of the VFD tube for (a,b) 1000-4000 rpm, (c,d) 4000-7000 rpm, and (e,f) 7000-9000 rpm, respectively. See text for further details.

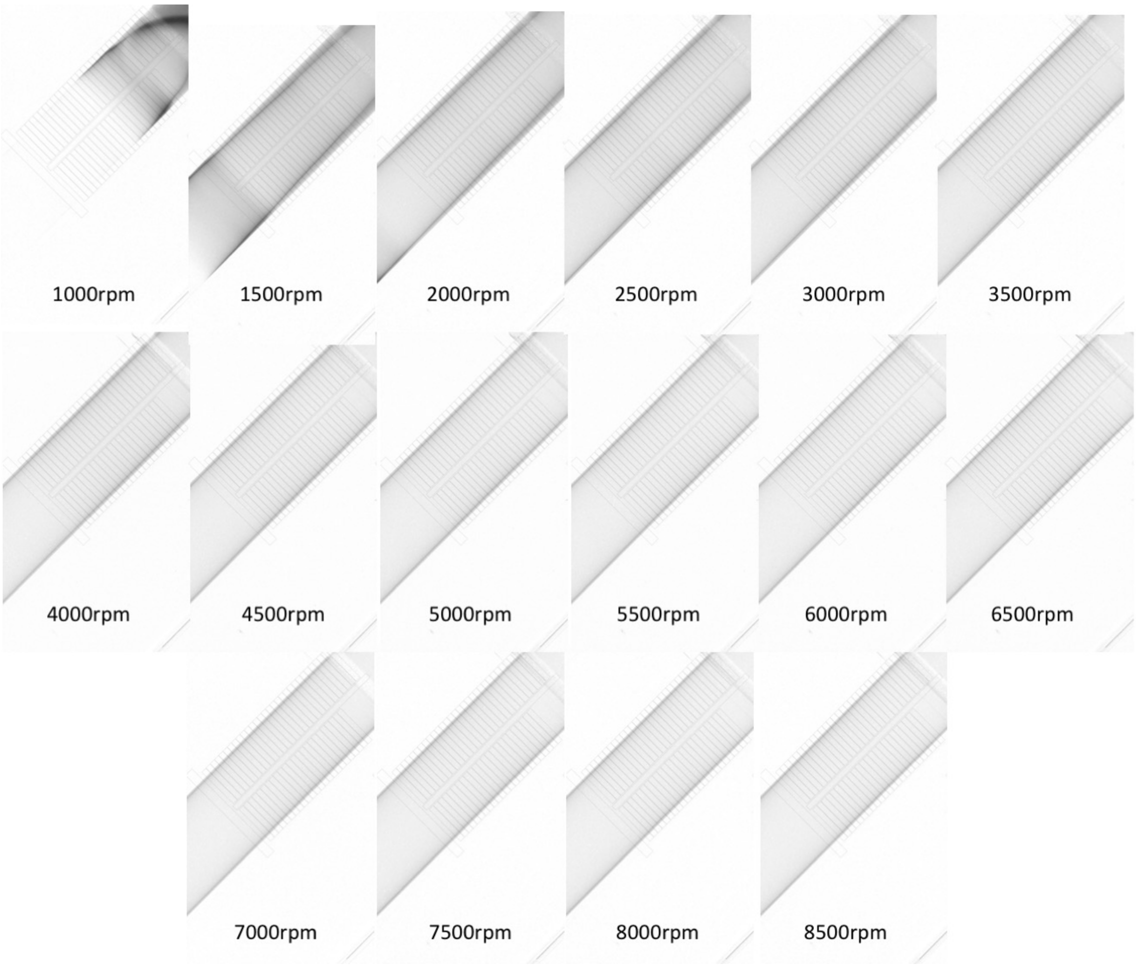

Figure S10: Neutron Images at different rotational speeds for a $9D_2O$:1Toluene mixture. Black boxes indicate the regions where attenuation cross sectional data is analysed.

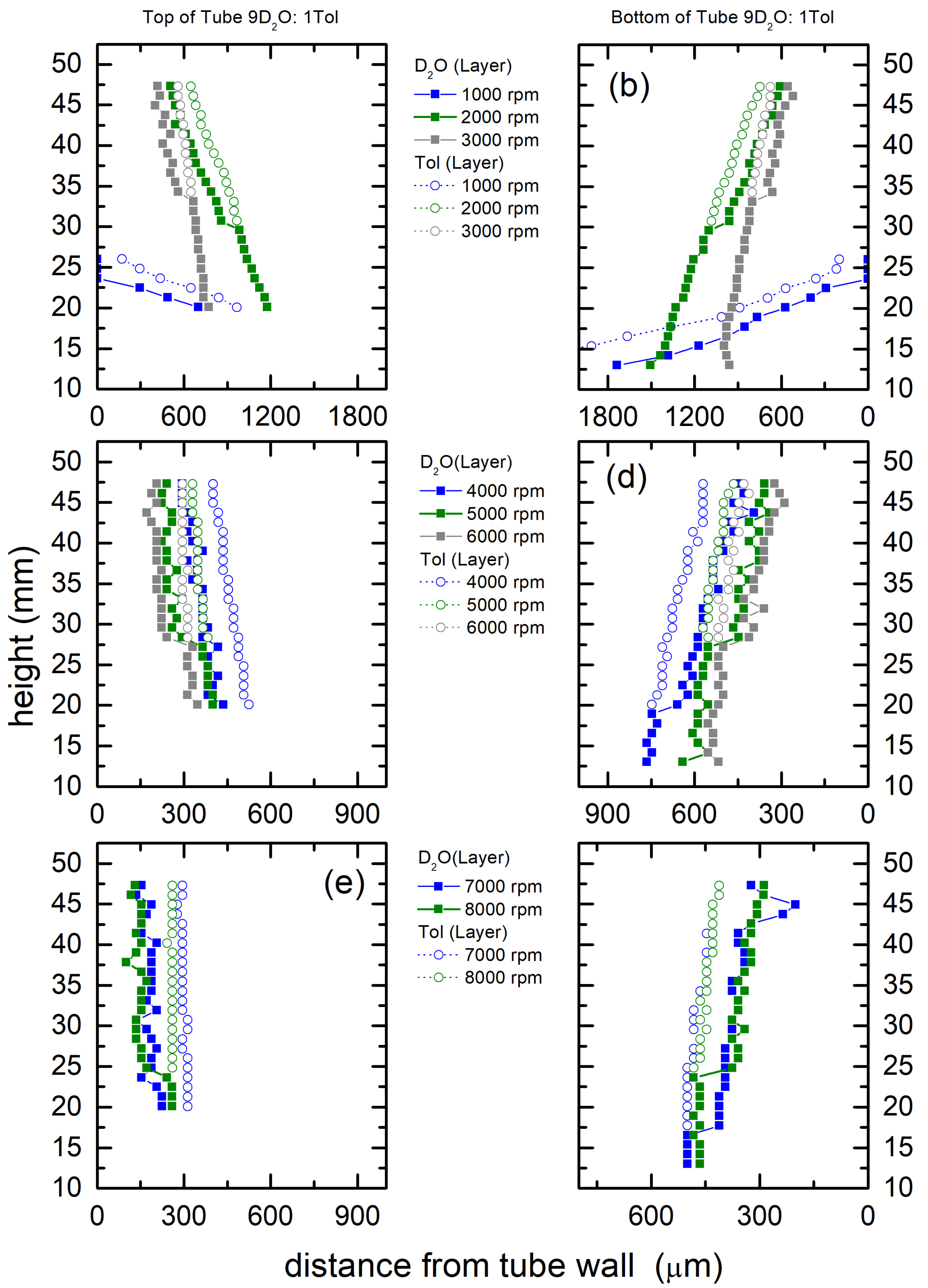

Figure S11: Characterisation of the film for a $9D_2O$:1Toluene mixture at the top and bottom of the VFD tube for (a,b) 1000-4000 rpm, (c,d) 4000-7000 rpm, and (e,f) 7000-9000 rpm, respectively. See text for further details.

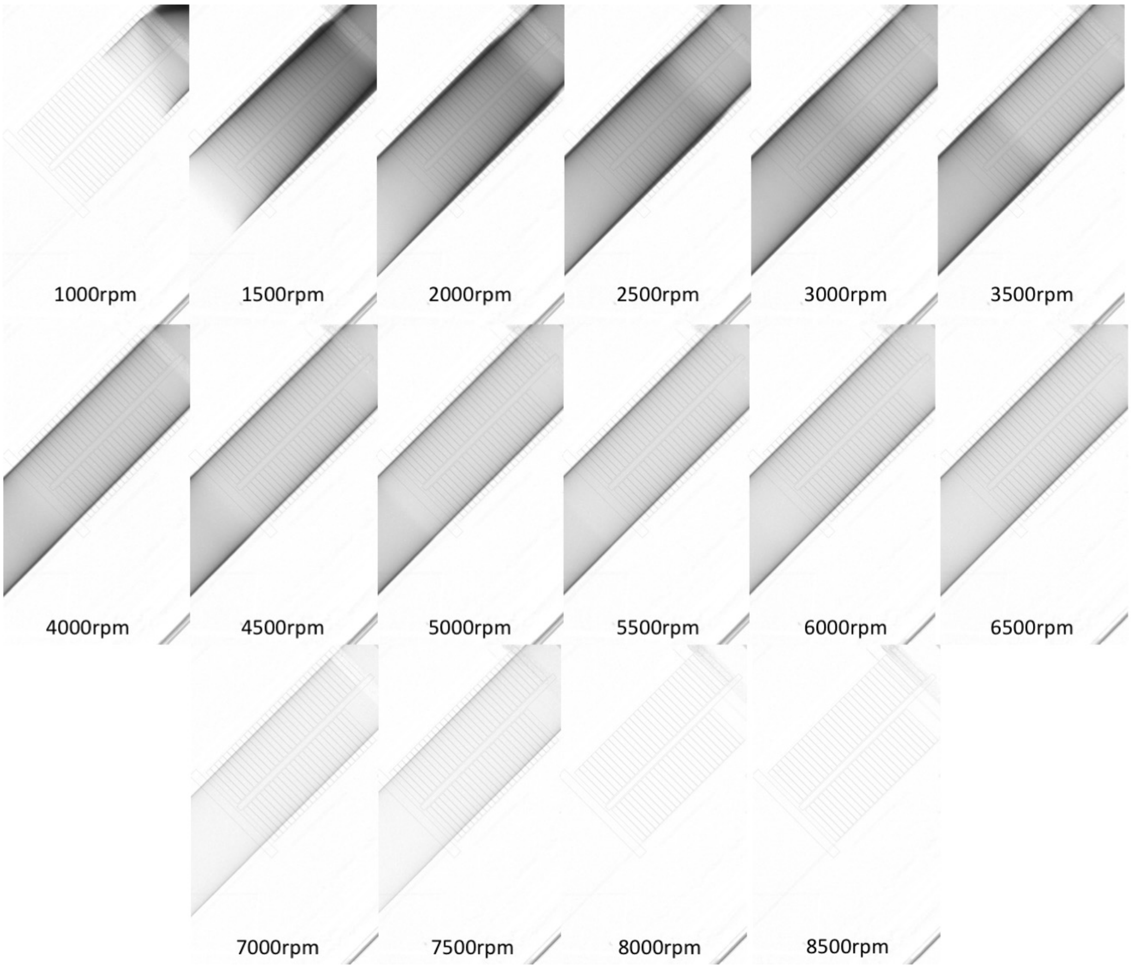

Figure S12: Neutron Images at different rotational speeds for a 1D_2O:3Toluene mixture. Black boxes indicate the regions where attenuation cross sectional data is analysed.

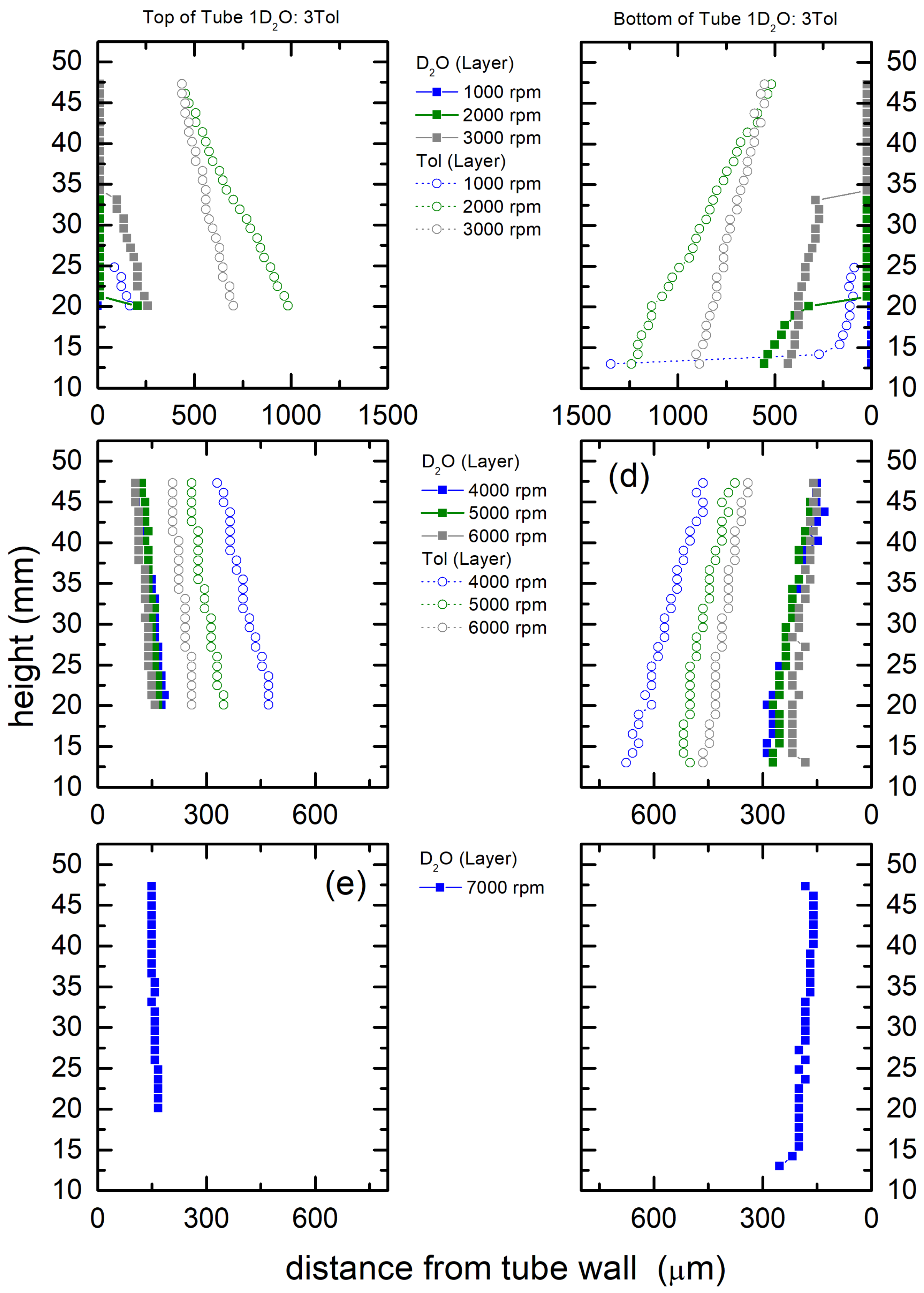

Figure S13: Characterisation of the film for a $1D_2O$:3Toluene mixture at the top and bottom of the VFD tube for (a,b) 1000-4000 rpm, (c,d) 4000-7000 rpm, and (e,f) 7000-9000 rpm, respectively. See text for further details.

S2. Mixing and demixing of immiscible liquids

Signatures of monophasic and biphasic systems

A FLIR (T62101) camera was used to record temperature variation of different liquids at rotational speeds between 3000 to 9000 rpm, 20 mm O.D for mimicking the continuous flow mode of operation at an inclination angle $\theta = 45^o$ in hemispherical and flat base tubes (where stated). The settings of the FTIR camera included emissivity 0.95, object distance 30.48 cm, relative humidity 50%, and the use of the rainbow colour pallet. All experiments were conducted in triplicate using the same quartz tube for each liquid for consistency of measurements within a temperature-controlled environment (average temperature 19 ^{o}C). In mimicking continuous flow processing sufficient liquid was added to the tube such that for any rotational speed, the generated thin film of liquid extends to the lip at the top of the tube. Mixing time in aqueous phase corresponds to the time taken for a drop of water containing a small amount of dye (blue) added at the bottom of the tube rotating at a specific speed to visibly uniformly mix with the fluid at the point halfway up the preformed film generated from 1 mL of water (measured in triplicates)[1]. Mixing time in organic phase corresponds to the time taken for a drop of organic solvent containing a small amount of beta-carotene added at the bottom of the tube rotating at a specific speed to visibly uniformly mix with the fluid at the point halfway up the preformed film generated from 1 mL of organic solvent (measured in triplicates). De-mixing times are observed immediately for the emulsions formed when an organic solvent and water are sonicated for at least 10 minutes. Placing 2 mL of the interface region between the two liquids in a 20 mm O.D hemispherical or flat base tube (where stated) the VFD is rotated between 3000 to 9000 rpm the time is recorded (120 seconds max) for the liquid to de-mix and become clear in the tube (Figure 2a). The solution is kept under sonication and all experiments were conducted in triplicate using the same quartz tube for each liquid mixture for consistency of measurements.

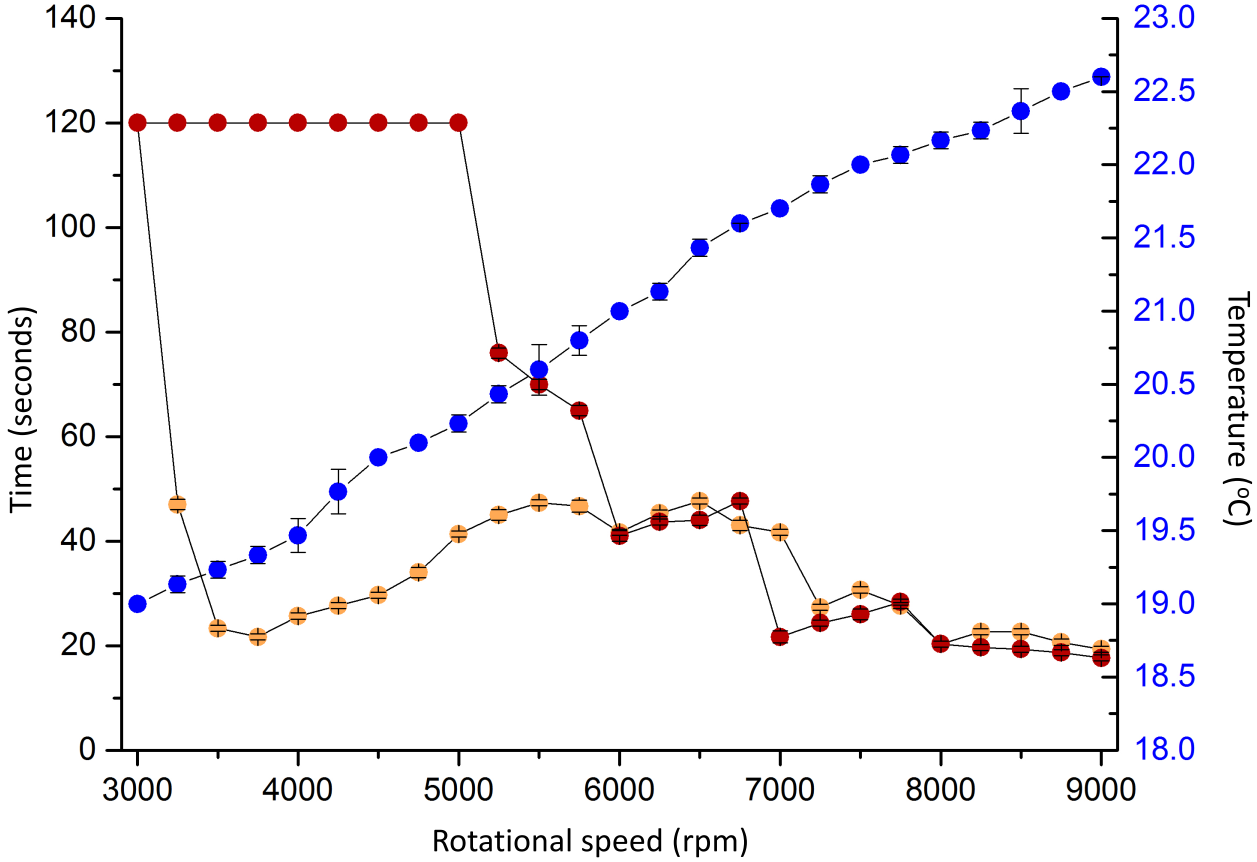

Figure S15: Signature of chlorobenzene and water, showing the mixing times of the aqueous phase (red), organic phase (orange) and the thermal response (blue) of a 1:1 mixture in a hemispherical base quartz tube (20 mm OD, 17.5 mm ID, 18.5 cm in length) as a function of ω at $\theta = 45^{o}$.

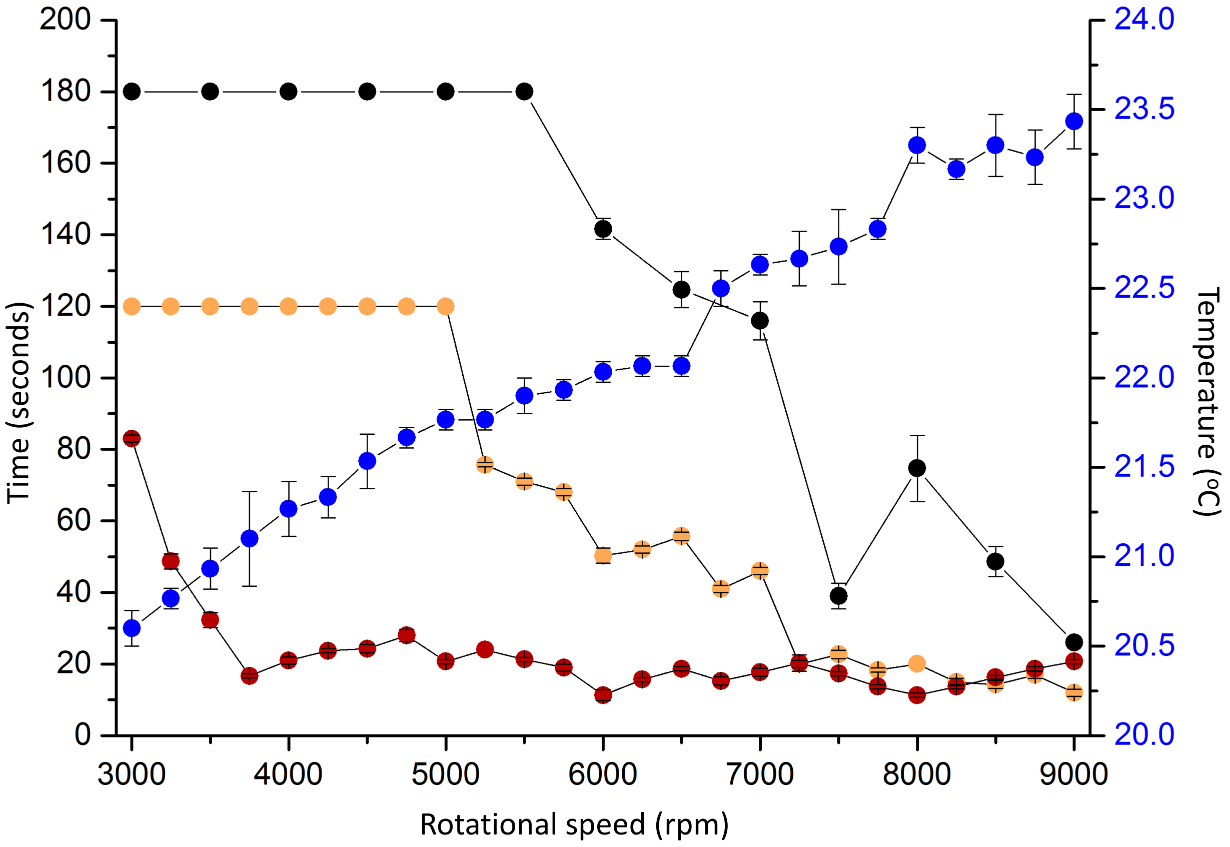

Figure S16: Signature of cyclohexene and water, showing the mixing times of the aqueous phase (red), organic phase (orange) and the thermal response (blue) of a 1:1 mixture in a hemispherical base quartz tube (20 mm OD, 17.5 mm ID, 18.5 cm in length) as a function of ω at $\theta = 45^{o}$.

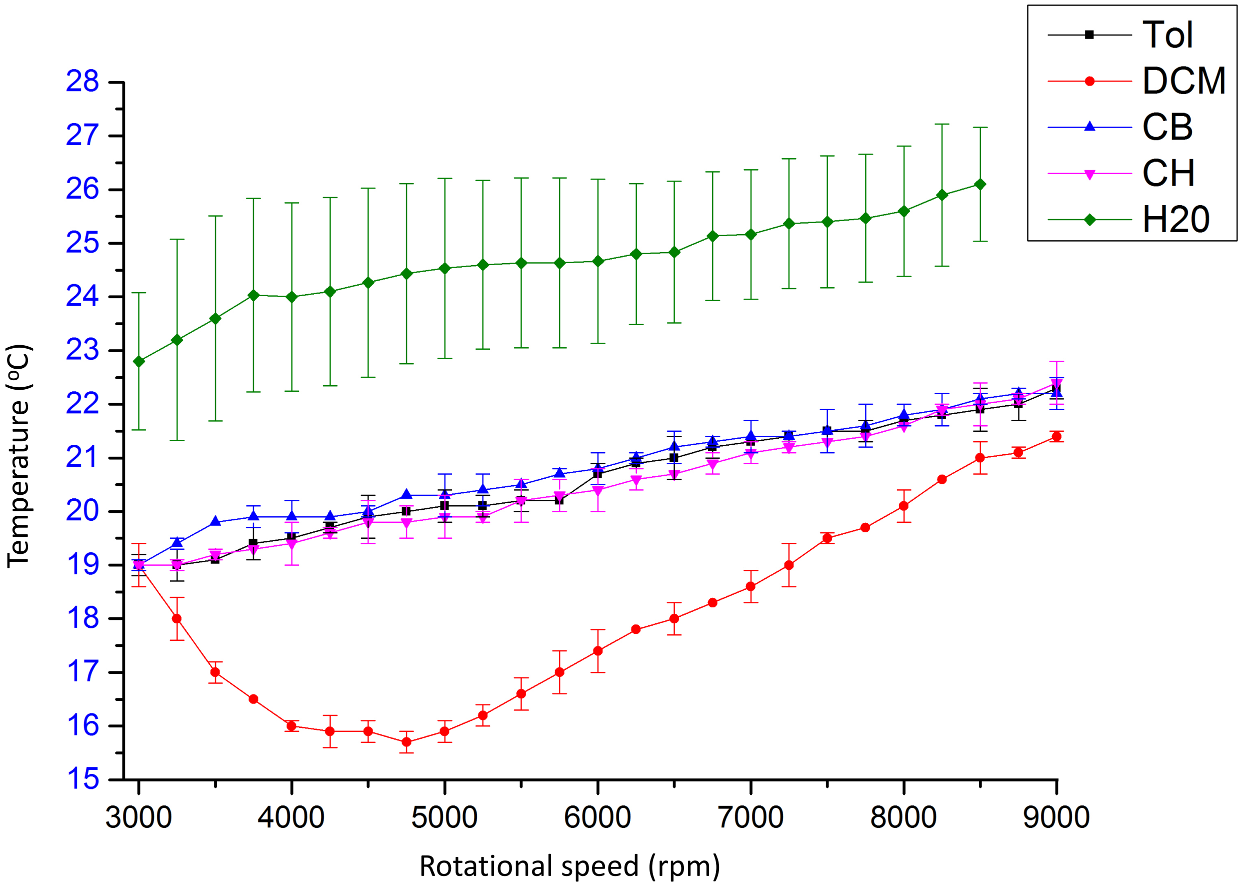

Figure S17: Thermal responses for various solvents (Tol: toluene, DCM; dichloromethane, CB: chlorobenzene, CH: cyclohexene) in a monophasic system in a flat base quartz tube (20 mm OD, 17.5 mm ID, 18.5 cm in length) as a function of ω at $\theta = 45^{o}$.

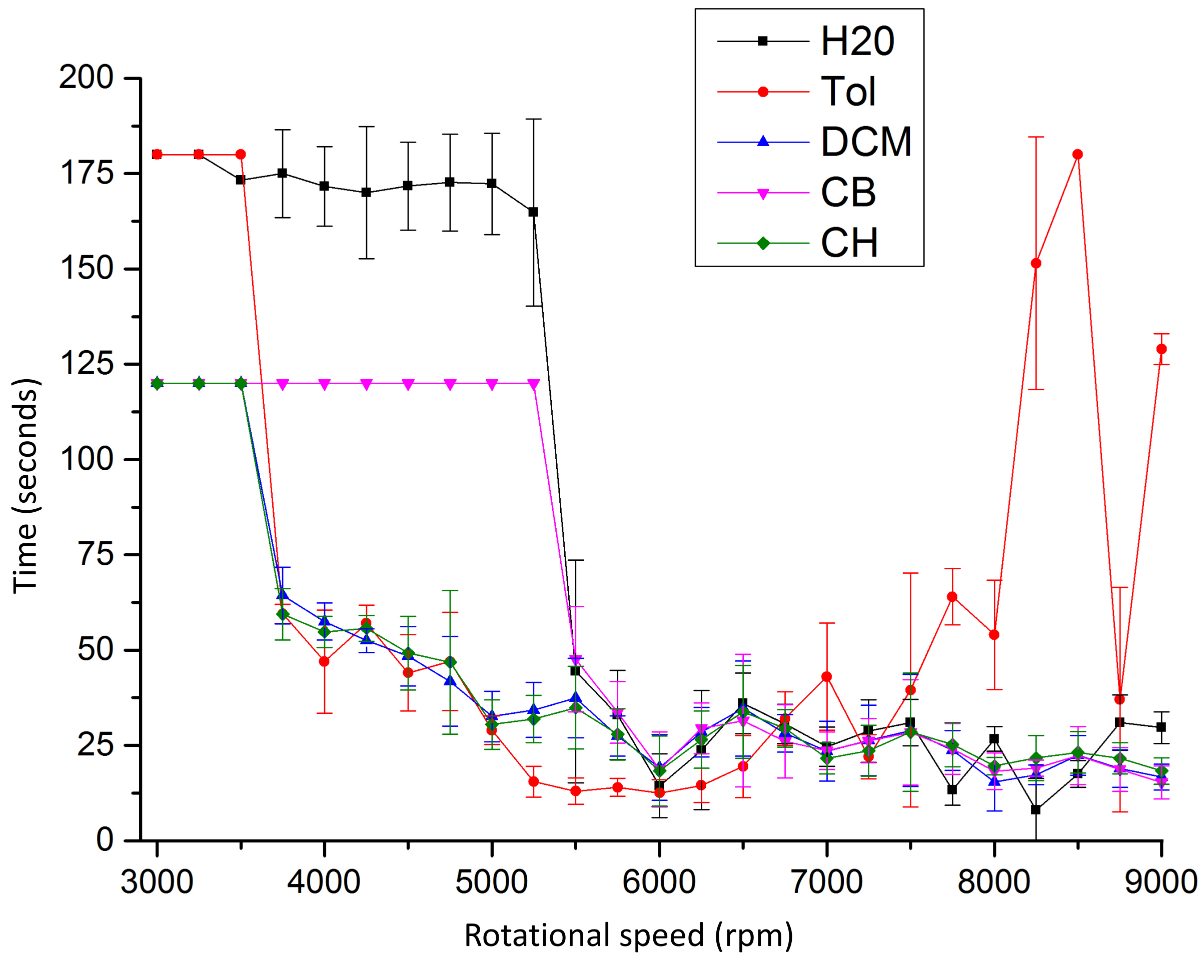

Figure S18: Mixing times for various solvents (Tol: toluene, DCM; dichloromethane, CB: chlorobenzene, CH: cyclohexene) in a monophasic system in a flat base quartz tube (20 mm OD, 17.5 mm ID, 18.5 cm in length) as a function of ω at $\theta = 45^{o}$.

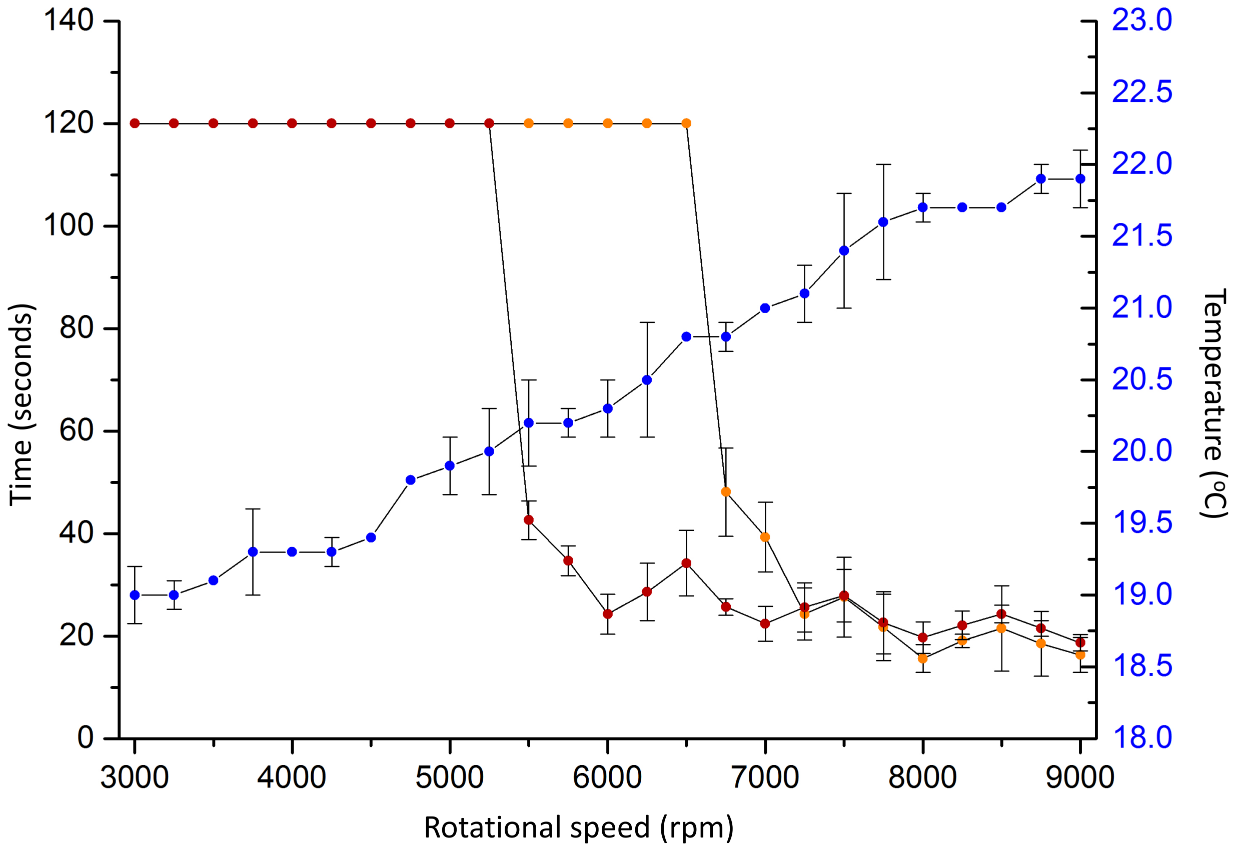

Figure S19: Signature of chlorobenzene and water, showing the mixing times of the aqueous phase (red), organic phase (orange) and the thermal response (blue) of a 1:1 mixture in a flat base quartz tube (20 mm OD, 17.5 mm ID, 18.5 cm in length) as a function of ω at $\theta = 45^{\circ}$.

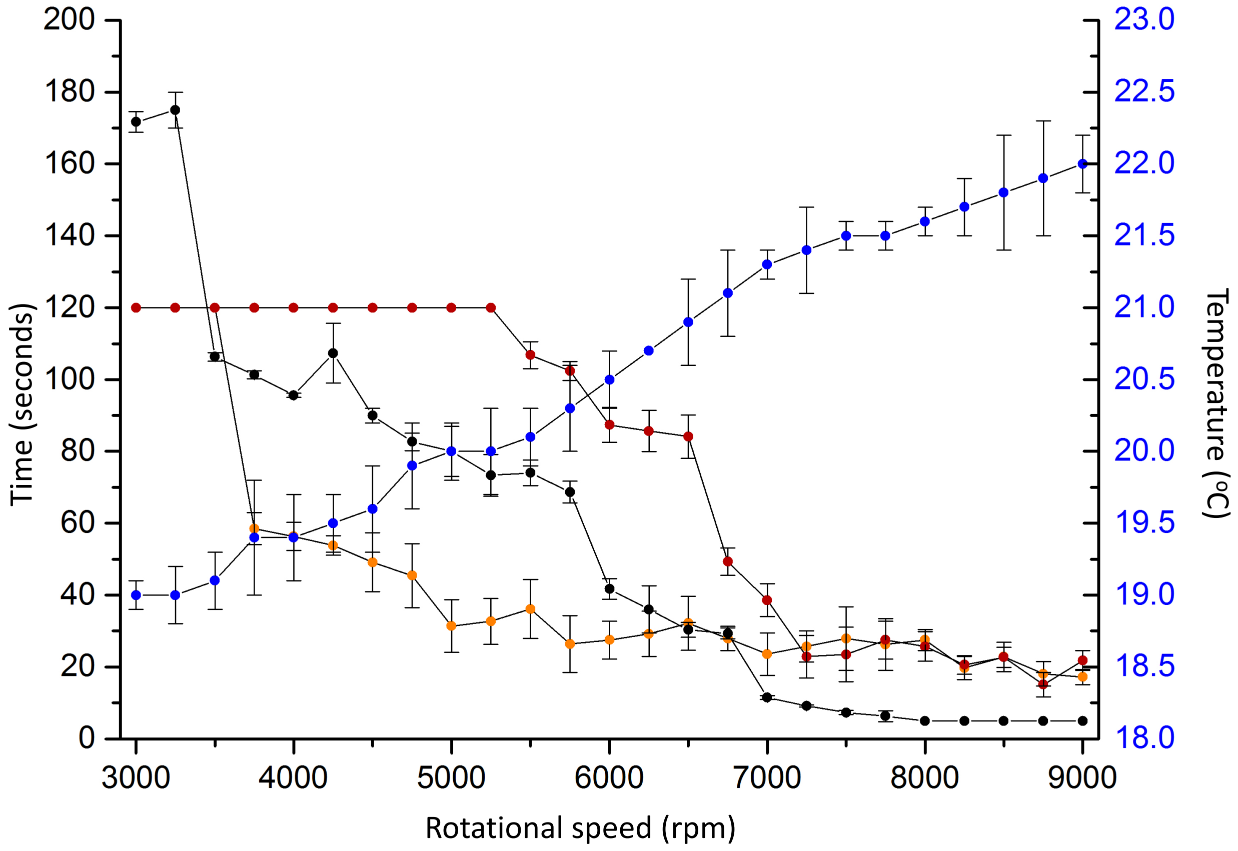

Figure S20: Signature of cyclohexene and water, showing the mixing times of the aqueous phase (red), organic phase (orange) and the thermal response (blue) of a 1:1 mixture in a flat base quartz tube (20 mm OD, 17.5 mm ID, 18.5 cm in length) as a function of ω at $\theta = 45^{o}$.

S3. Equal density immiscible liquids

To investigate the effect of density on the processing of immiscible liquids in the VFD, H_2O and chlorobenzene were spun at speeds between 3000-9000 rpm, the mass of the tube post processing was recorded and the volume of fluid remaining in the tube calculated from this. calcium chloride was added to H_2O, increasing its density to 1.11 g/mL, the density of chlorobenzene. To confirm the densities of $H_2O.CaCl_2$ and chlorobenzene were approximately equal the solutions were mixed. Visual confirmation of density was repeated prior to each experimental run to ensure the densities were equal within experimental limits. To ensure the native film volume, in the absence of a second liquid was known the experiments were repeated for H_2O alone, $H_2O.CaCl_2$ and chlorobenzene. All experiments were performed in triplicate and repeated with a second tube to determine the effect of the tube on the final volume. The experiments were repeated on a second VFD to account for intermachine variability.

No difference in final volume was found between H_2O and $H_2O.CaCl_2$ experiments (Table S1). Limited variation was found between VFDs, however, significant differences in final masses and subsequently volumes were found between tubes. This is likely caused by natural variation in surface of manufactured tubes. The percentage change in volume as speed increases was uniform regardless of the mixture examined, however chlorobenzene alone had consistently lower final tube volumes than mixtures containing water (Fig S21(a)). This is likely a result of evaporative losses due to comparatively low vapour pressure of chlorobenzene. By normalising volumes to that of chlorobenzene for each tube, comparison between tubes is facilitated (Fig S21 (b)). This shows that alteration of water density does not affect the final tube volumes. Final volume in this instance is a proxy for average film thickness indicating that the density of immiscible systems has limited impact on the overall film thickness during processing.

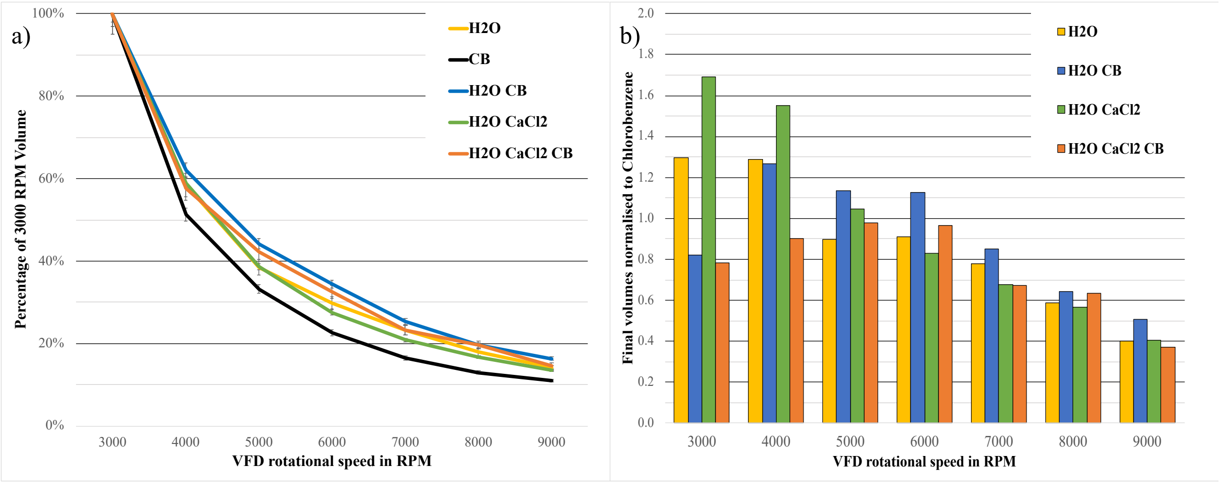

Figure S21: **(a)** The percentage change in volume as processing speed increases for H_2O, H_2O $CaCl_2$ solutions, chlorobenzene (CB) and mixtures thereof. **(b)** Final tube volumes normalised to chlorobenzene (CB).

Table S1: Mean residual volume following VDF processing of water, water with calcium chloride to a density of 1.1 g/mL, chlorobenzene (CB) and mixtures thereof.

		Average Final Volume (mL) (standard deviation)						
		3 krpm	4 krpm	5 krpm	6 krpm	7 krpm	8 krpm	9 krpm
Tube 1	H_2O	9.489 (0.143)	5.699 (0.244)	3.842 (0.244)	2.871 (0.091)	2.162 (0.069)	1.670 (0.075)	1.358 (0.075)
	$H_2O.CaCl_2$	9.436 (0.146)	5.617 (0.132)	3.778 (0.077)	2.776 (0.100)	2.104 (0.026)	1.671 (0.070)	1.359 (0.081)
	H_2O CB	8.953 (0.435)	5.382 (0.275)	3.751 (0.273)	2.998 (0.096)	2.284 (0.126)	1.750 (0.136)	1.376 (0.058)
	$H_2O.CaCl_2$ CB	8.150 (0.435)	4.484 (0.375)	3.424 (0.215)	2.613 (0.180)	2049 (0.220)	1.608 (0.136)	1.258 (0.129)
	CB	7.545 (0.353)	3.997 (0.211)	2.587 (0.155)	1.850 (0.126)	1.303 (0.118)	0.987 (0.149)	0.855 (0.117)
Tube 2	H_2O	7.612 (0.300)	4.322 (0.097)	2.775 (0.080)	2.243 (0.049)	1.790 (0.101)	1.387 (0.102)	1.053 (0.077)
	$H_2O.CaCl_2$	8.457 (0.055)	4.930 (0.091)	3.142 (0.058)	2.170 (0.084)	1.641 (0.092)	1.339 (0.074)	1.056 (0.041)
	H_2O CB	7.203 (0.260)	4.598 (0.264)	3.346 (0.107)	2.545 (0.095)	1.813 (0.1340	1.414 (0.078)	1.247 (0.056)
	$H_2O.CaCl_2$ CB	7.926 (0.723)	4.767 (0.520)	3.358 (0.188)	2.611 (0.226)	1.691 (0.088)	1.541 (0.139)	1.094 (0.124)
	CB	6.965 (0.314)	3.449 (0.113)	2.237 (0.175)	1.440 (0.050)	1.090 (0.116)	0.892 (0.252)	0.753 (0.178)

S4. In situ moulding of topological fluid flow impacting on the surface of the tube

Molecular drilling in polysulfone (PSF)

To generate a uniform thin-layer adhering to the inner surface of the VFD tube, 1.4 mL of 100 mg/mL of PSF dissolved in dichloromethane (DCM), which involved adding a solution of the polymer to a VFD tube (20 mm OD, 17.5 mm ID) with the operational condition mentioned in earlier research[1], tube tilted at 5° which was then spun at 6000 rpm for ~15 min. The formation of the thin-layer was due to the facilitated evaporation caused by high mass and heat transfer in the open end tube, followed by washing with hexane and drying under a flow of dry nitrogen. Three different samples were taken from the peeled off polymeric thin layer from the tube Sections of the film was then peeled from the surface of the tube at three different locations, Figure S22. The thin layer created in the VFD was relatively uniform in thickness, smooth and without any structure variation.

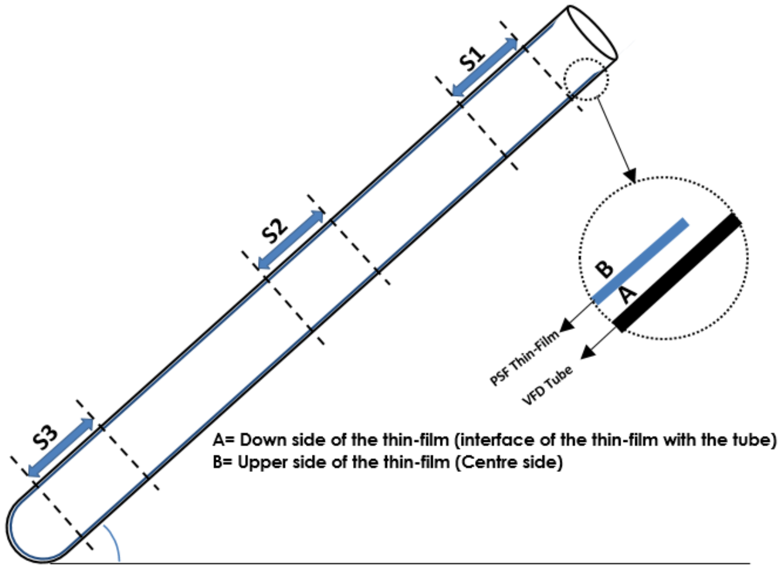

Figure S22: Locations of samples S1, S2 and S3 taken from the PSF thin-layer formed in the VFD; sample S1A and S1B refer to the interface of the polymer layer with the tube wall and the liquid film from the top of the tube respectively, and similarly S2A and S2B, and S3A and S3B, from the middle and bottom of the tube[1].

Previously we investigated the fluid flow in the monophasic system (mainly toluene) in a hemispherical base tube with the fluid flow investigated herein in a biphasic system (1:1 toluene and water) in both hemispherical and flat base tubes[1]. To do this, two sets of experiments were conducted in the VFD using toluene and water (1:1) which were added to

the polymeric coated VFD. VFD processing of the thin layer was conducted in the tube at operational conditions mentioned in Table S2. The polymer is only sparingly soluble in toluene, and in each experiment the tube was rotated at different rotational speeds rpm at $\theta = 45^{o}$, at room temperature, for 30 minutes. After processing, the liquid was drained, and the layer of polymer washed with hexane several times and purged with nitrogen gas for drying purposes, for 2h.

Three samples of every processed thin film were removed from each tube and studied using SEM, Figure S22-S23.

The results revealed arrays of indentations and some holes on the polymer – tube interfaces which have been introduced in our earlier research as molecular drilling occurred due to the double helical flow in the VFD[1]. SEM imaging the other side of the PSF layer (side B) post-VFD processing using the same conditions also showed some molecular drilled holes which was absent in earlier research in monophasic. This could be related to the effect of water which is a non-solvent for the drilled polymer and would prevent smoothing the holes which were drilled by toluene, (Figure S23 – S34). The experiments were conducted in different rotational speeds (ω 3500, 4500, 5500, 6500, 7000, 7500 rpm) in both hemispherical and flat base tubes. Given the structures revealed on the surface adjacent to the tube wall (side A) for biphasic (toluene and water), all subsequent processing was done in toluene and water followed by washing with hexane.

Molecular drilling experiments

Table S2: Experiments for determining the 'molecular drilling' on a PSF thin film in the VFD tube in biphasic system (1:1 toluene: water), in high shear fluid flow at room temperature. *

Sample	Rotational Speed (k rpm)	θ (°)	Volume of Toluene (mL)	Volume of Water (mL)	Time (min)	Tube base
Thin film	6	5	----	----	15	Hemispherical
1	3.5	45	3	3	30	Hemispherical
2	4.5	45	2.25	2.25	30	Hemispherical
3	5.5	45	1.5	1.5	30	Hemispherical
4	6	45	1	1	30	Hemispherical
5	7	45	0.75	0.75	30	Hemispherical
6	7.5	45	0.6	0.6	30	Hemispherical
Thin film	6	5	----	----	15	Flat
7	3.5	45	3	3	30	Flat
8	4.5	45	2.25	2.25	30	Flat
9	5.5	45	1.5	1.5	30	Flat
10	6	45	1	1	30	Flat
11	7	45	0.75	0.75	30	Flat
12	7.5	45	0.6	0.6	30	Flat

* Notes:

(i) The first entry for every tube (flat or hemispherical base) is for creating the PSF thin-layer in a VFD tube (20 mm OD, 17.5 mm ID) from a solution of the polymer in dichloromethane at 6000 rpm, with θ = 5° to ensure the layer is formed close to the full length of the tube during the evaporation of the solvent, ca 15 min.

(ii) The volume of polymer solution for each sample corresponds to that required to create a layer of polymer across the entire length of the tube at θ = 5°, with the then 'molecular drilling' experiments carried out at θ = 45°.

(iii) The volume of the biphasic system at each rotation speed was selected to ensure the film reached the top of the tube.

(iv) The layer of PSF in the VFD were washed with hexane and purged with nitrogen gas prior to peeling off the glass tube.

(v) Three sections of the layers (S1, S2 and S3, Fig. S22) were taken from every sample, with both sides (A and B) studied using SEM.

A comprehensive statistical study has been done by measuring the holes' size distribution in every sample using ImageJ software and then were plotted using OriginPro 9 while using Gaussian fitting curves.

Molecular drilling in “Hemispherical” base tube in biphasic system:

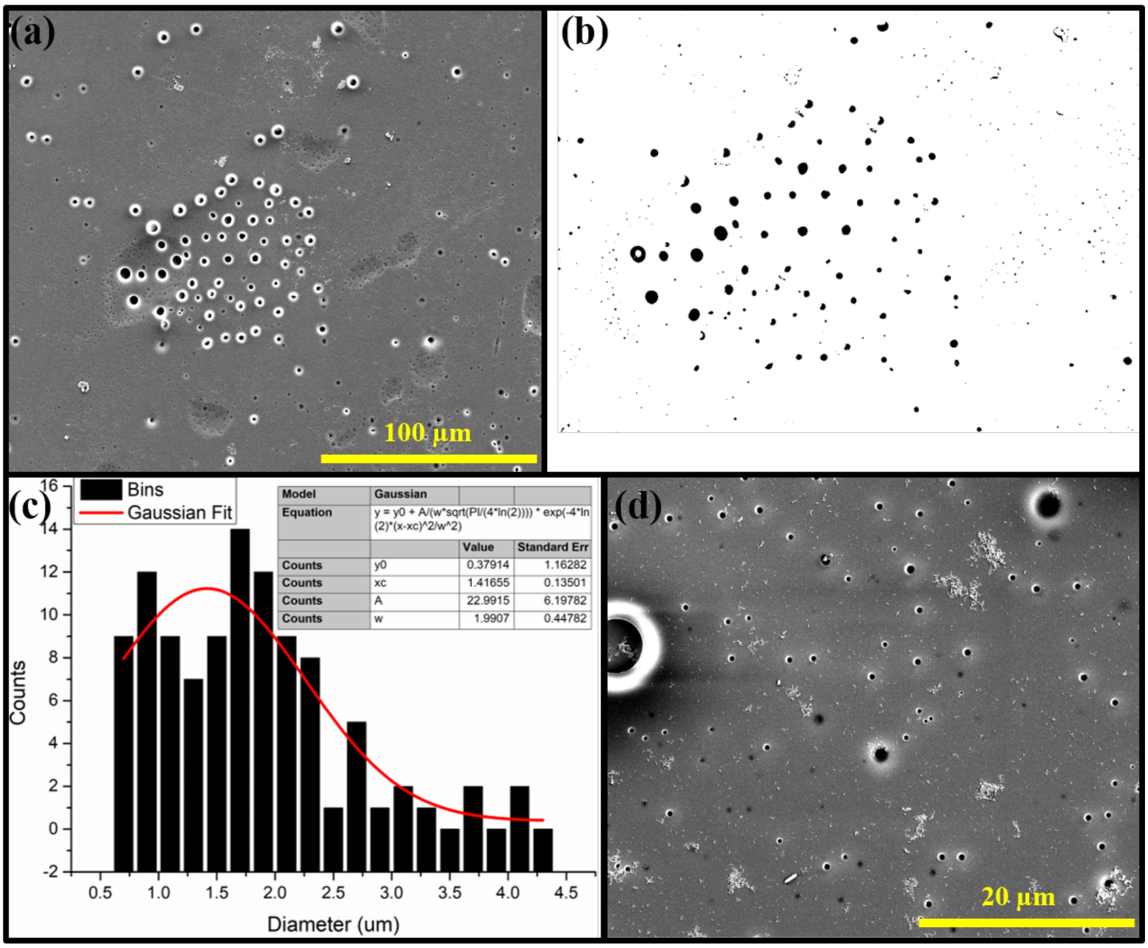

Figure S23: (a) SEM image of molecular drilled holes on polymer – tube interface of the thin-layer (Side A) processed in biphasic system of toluene and water (1:1), which was processed at 3500 rpm, θ = 45°, room temperature, and 30 min processing in a hemispherical base tube, then washed with hexane, and dried under nitrogen gas, (b) ImageJ analysis of the sample identifying the drilled holes, (c) Gaussian fitted curve to the distribution of drilled holes observed in the sample and (d) SEM image of polymer – liquid interface of the thin-layer (Side B).

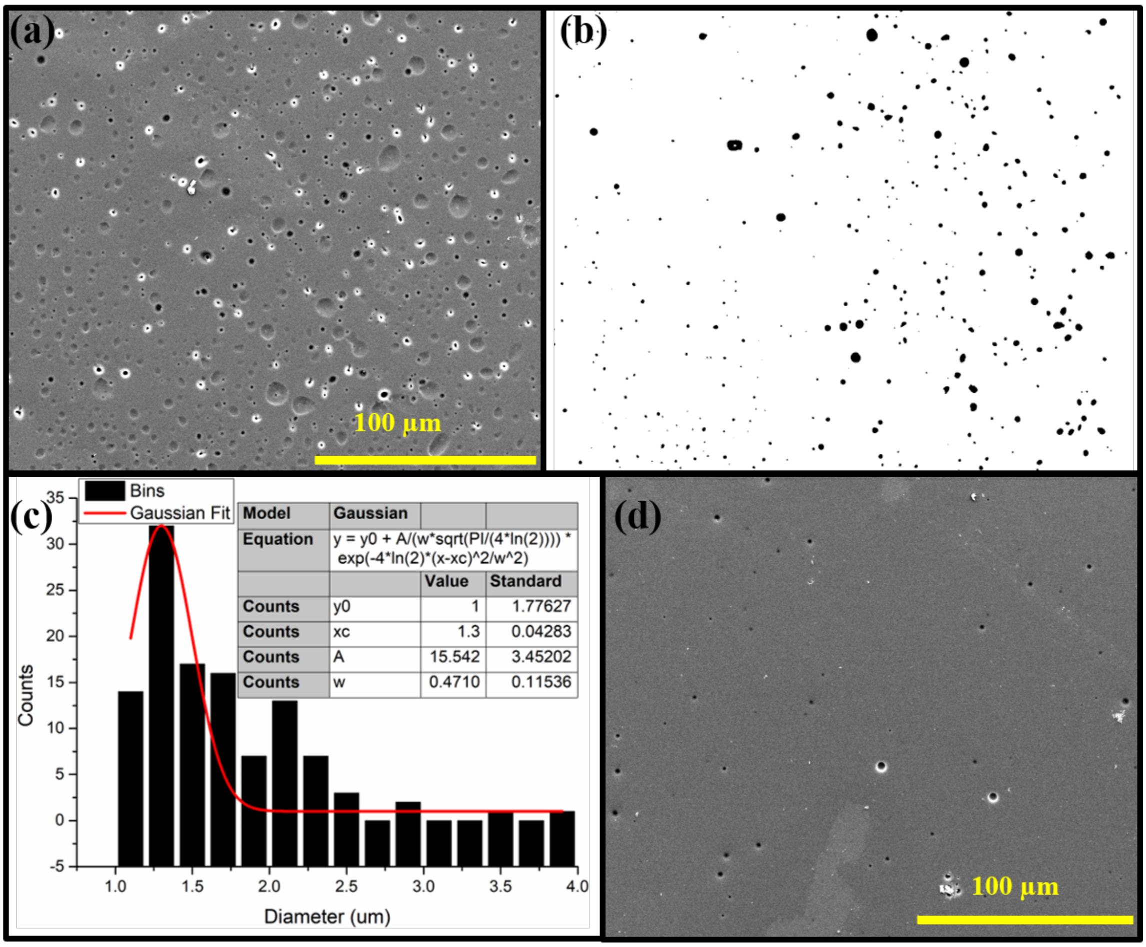

Figure S24: (a) SEM image of molecular drilled holes in the polymer at the polymer – tube interface of the thin-layer (Side A) processed in biphasic system of toluene and water (1:1), which was processed at 4500 rpm, θ = 45°, room temperature, and 30 min processing in hemispherical base tube, then washed with hexane, and dried under nitrogen gas, (b) ImageJ analysis of the sample identifying the drilled holes, (c) Gaussian fitted curve to the distribution of drilled holes observed in the sample and (d) SEM image of polymer – liquid interface of the thin-layer (Side B).

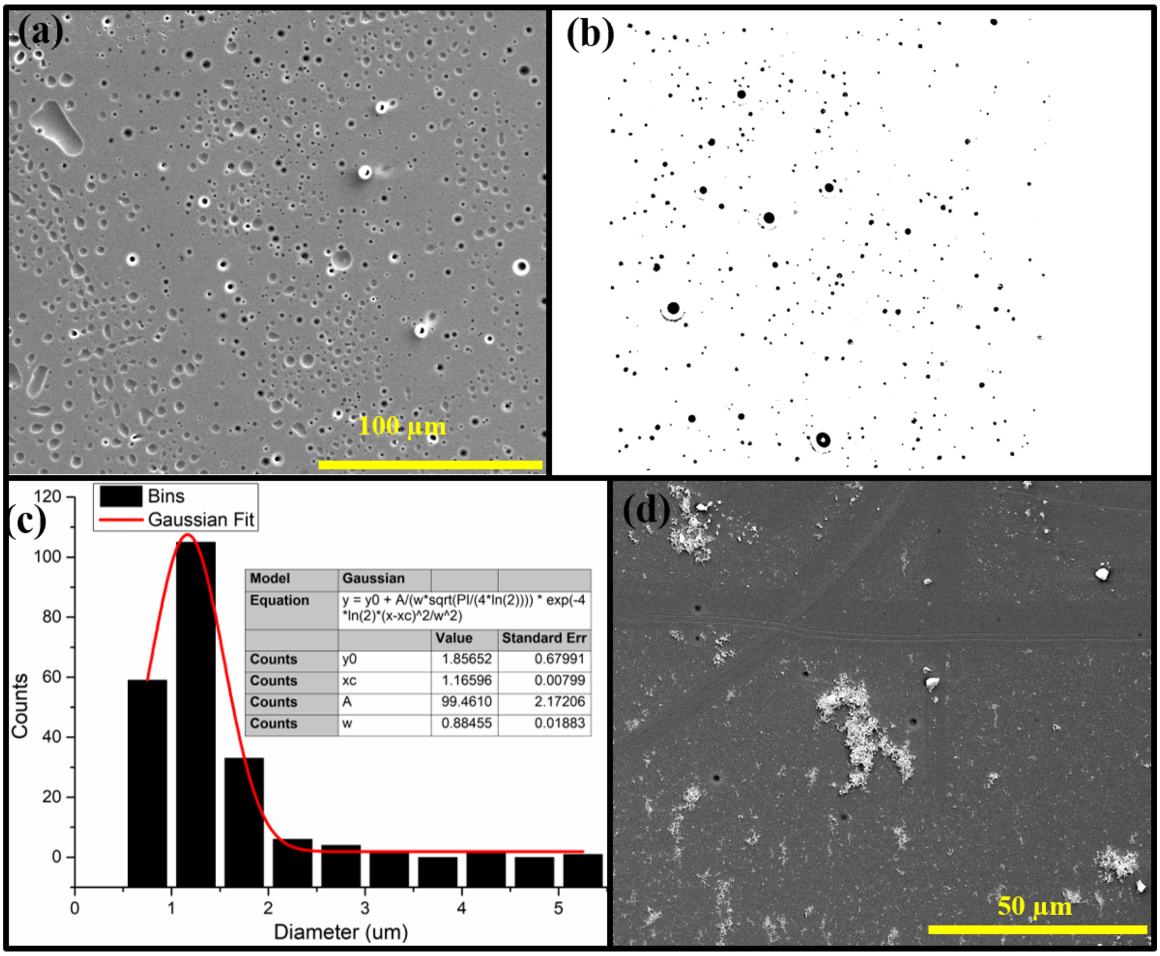

Figure S25: (a) SEM image of molecular drilled holes in the polymer at the polymer – tube interface of the thin-film (Side A) processed in biphasic system of toluene and water (1:1), which was processed at 5500 rpm, θ = 45°, room temperature, and 30 min processing in hemispherical base tube, then washed with hexane, and dried under nitrogen gas, (b) ImageJ analysis of the sample identifying the drilled holes, (c) Gaussian fitted curve to the distribution of drilled holes observed in the sample and (d) SEM image of polymer – liquid interface of the thin-layer (Side B).

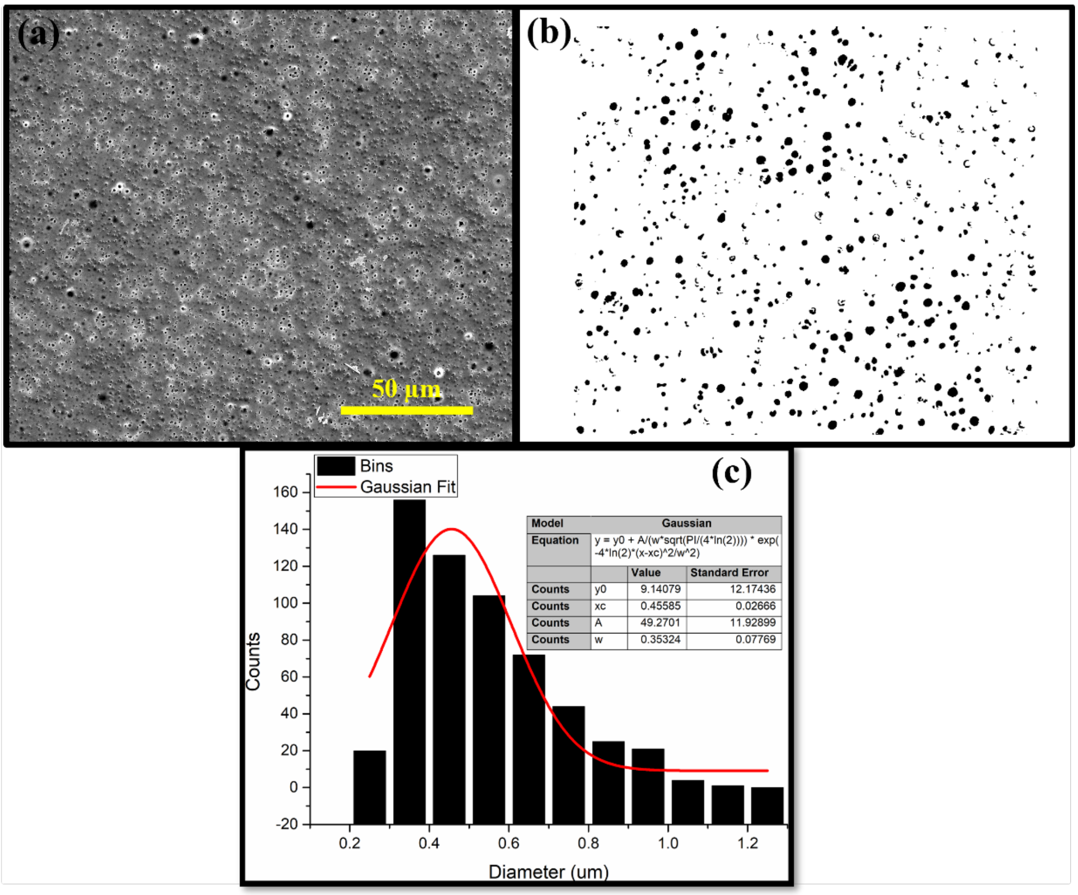

Figure S26: (a) SEM image of molecular drilled holes in the polymer at the polymer – tube interface of the thin-film (Side A) processed in biphasic system of toluene and water (1:1), which was processed at 6.5k rpm, θ = 45°, room temperature, and 30 min processing in hemispherical base tube, then washed with hexane, and dried under nitrogen gas, (b) ImageJ analysis of the sample identifying the drilled holes, (c) Gaussian fitted curve to the distribution of drilled holes observed in the sample.

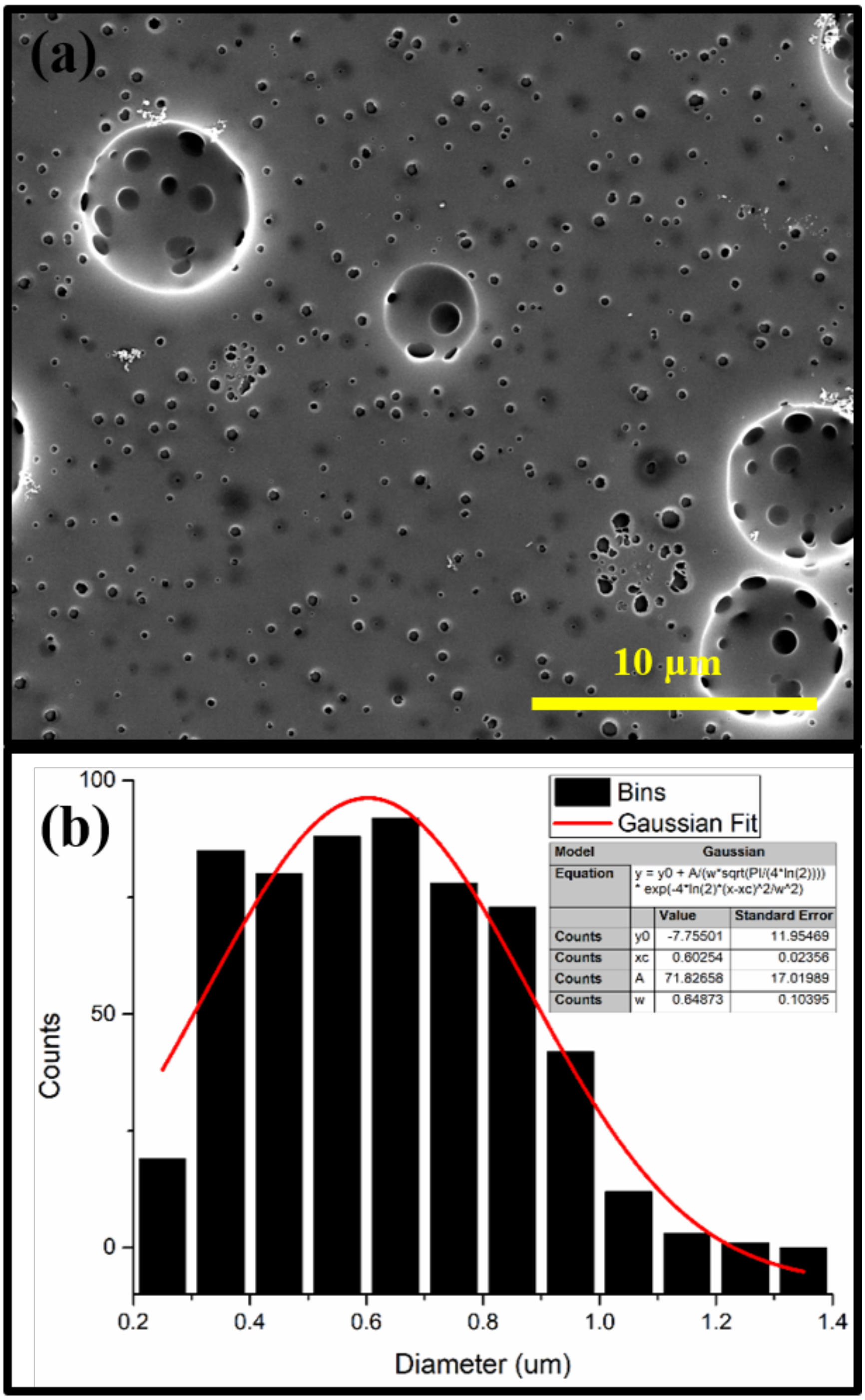

Figure S27: (a) SEM image of molecular drilled holes in the polymer at the polymer – tube interface of the thin-layer (Side A) processed in biphasic system of toluene and water (1:1), at 7000 rpm, θ = 45°, room temperature, and 30 min processing in hemispherical base tube, then washed with hexane, and dried under nitrogen gas, and (b) Gaussian fitted curve to the distribution of drilled holes observed in the polymer at the polymer – tube interface of the thin-layer.

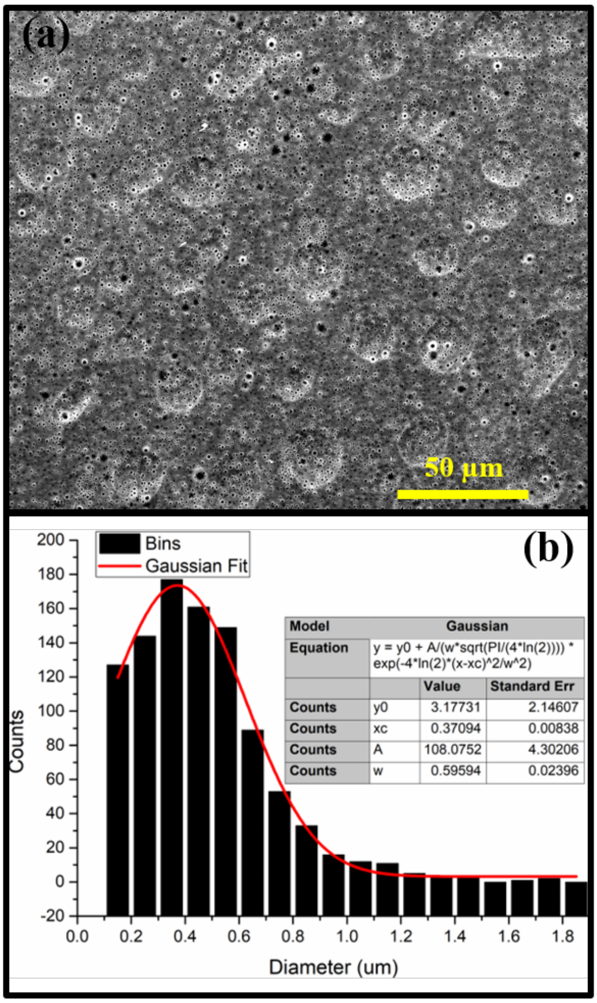

Figure S28: (a) SEM image of molecular drilled holes in the polymer at the polymer – tube interface of the thin-layer (Side A) processed in biphasic system of toluene and water (1:1), at 7500 rpm, θ = 45°, room temperature, and 30 min processing in hemispherical base tube, then washed with hexane, and dried under nitrogen gas, and (b) Gaussian fitted curve to the distribution of drilled holes observed in the polymer at the polymer – tube interface of the thin-film.

Molecular drilling in "Flat" base tube in biphasic system:

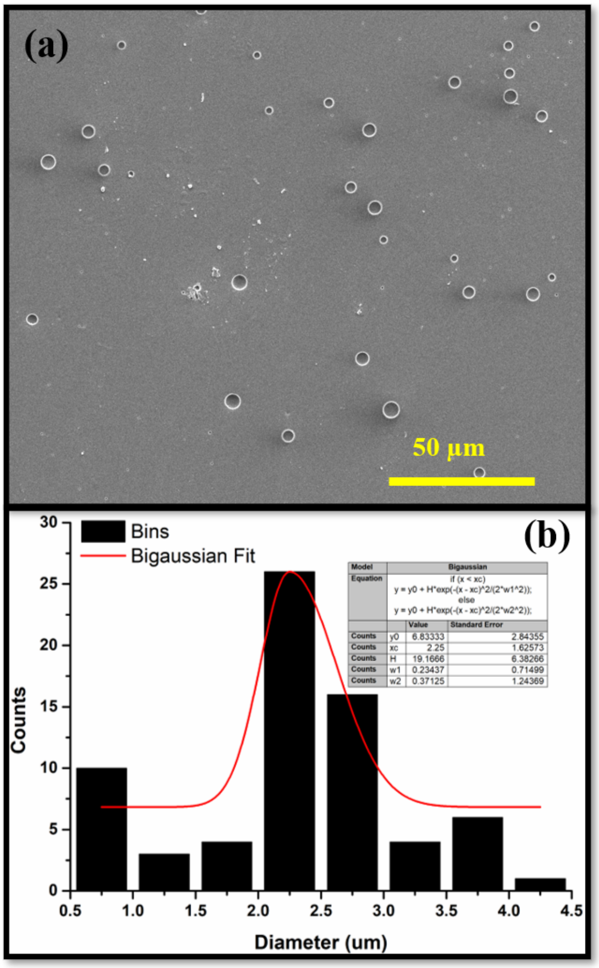

Figure S29: SEM image of molecular drilled holes in the polymer at the on polymer – tube interface of the thin-layer (Side A) processed in biphasic system of toluene and water (1:1), at 3500 rpm, θ = 45°, room temperature, and 30 min processing in a flat base tube, then washed with hexane, and dried under nitrogen gas, and (b) Gaussian fitted curve to the distribution of drilled holes observed in the polymer at the polymer – tube interface.

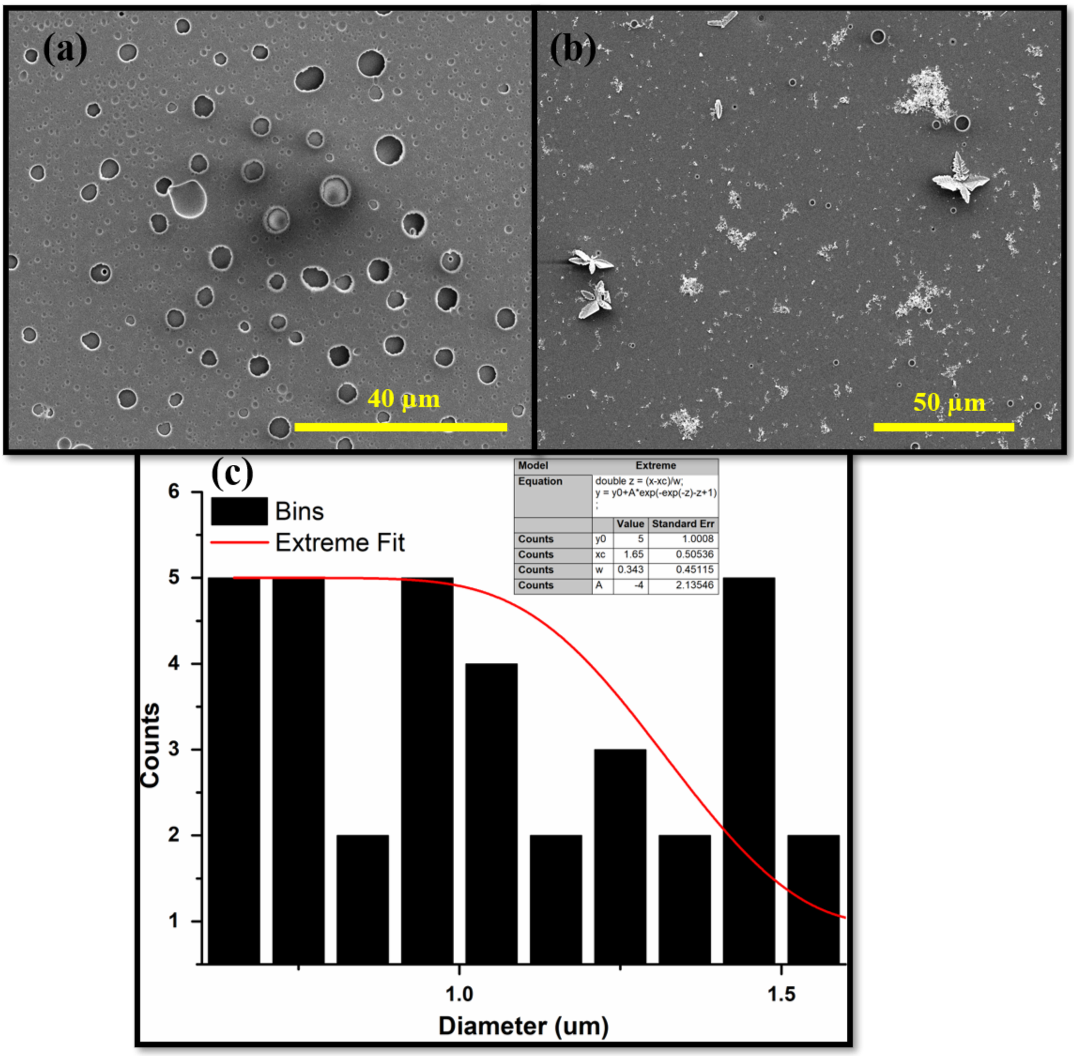

Figure S30: (a) SEM image of molecular drilled holes in the polymer at the polymer – tube interface of the thin-layer (Side A), (b) SEM image of polymer – liquid interface of the thin-film (Side B) processed in biphasic system of toluene and water (1:1), at 4500 rpm, θ = 45°, room temperature, and 30 min processing in a flat base tube, then washed with hexane, and dried under nitrogen gas, (c) Gaussian fitted curve to the distribution of drilled holes observed in the polymer at the polymer – tube interface (Side A).

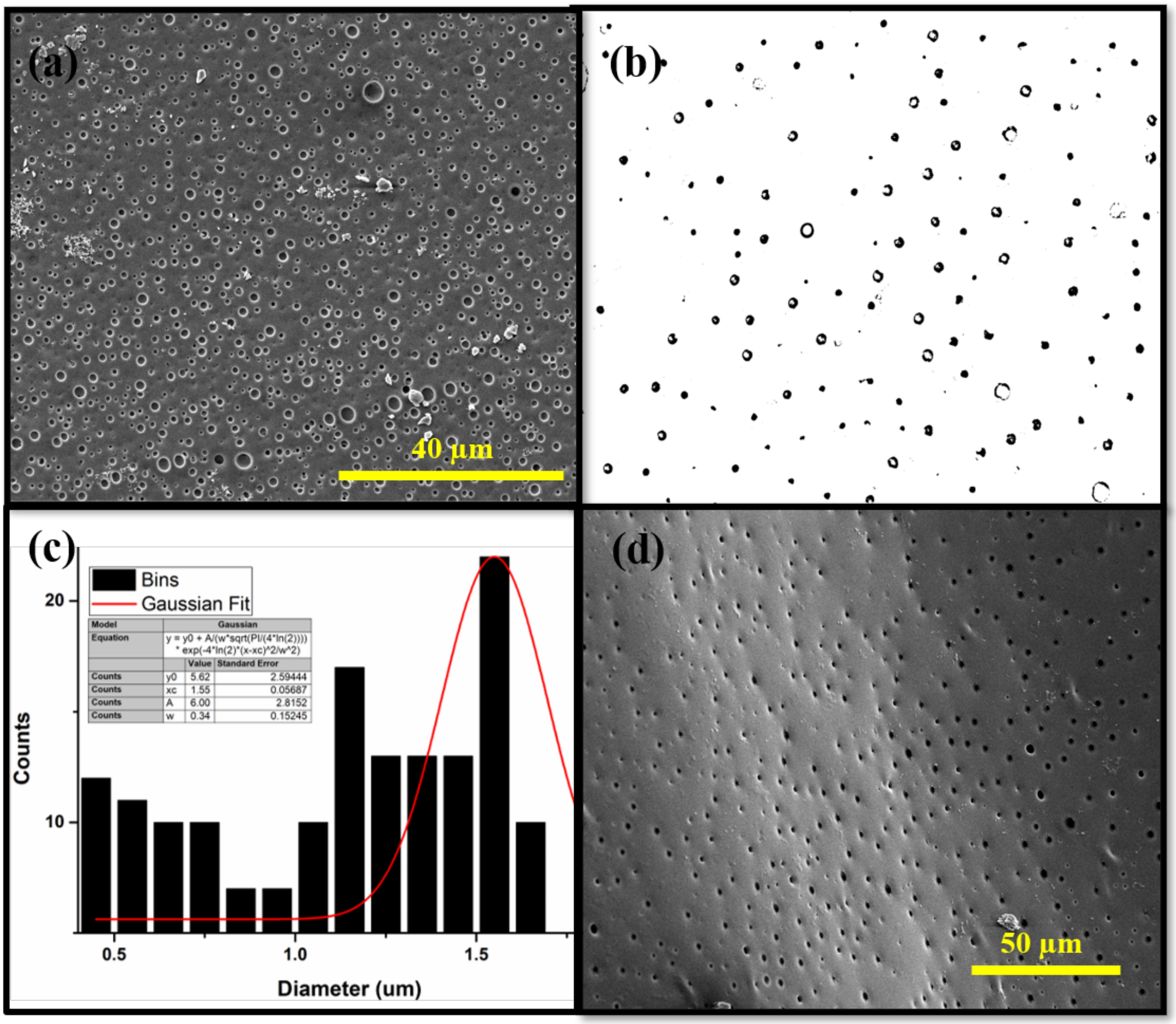

Figure S31: (a) SEM image of molecular drilled holes in the polymer at the polymer – tube interface of the thin-layer (Side A) processed in biphasic system of toluene and water (1:1) at 5500 rpm, θ = 45°, room temperature, and 30 min processing in a flat base tube, then washed with hexane, and dried under nitrogen gas, (b) ImageJ analysis of the sample identifying the drilled holes, (c) Gaussian fitted curve of the sample and (d) SEM image of polymer at the polymer – liquid interface of the thin-layer (Side B).

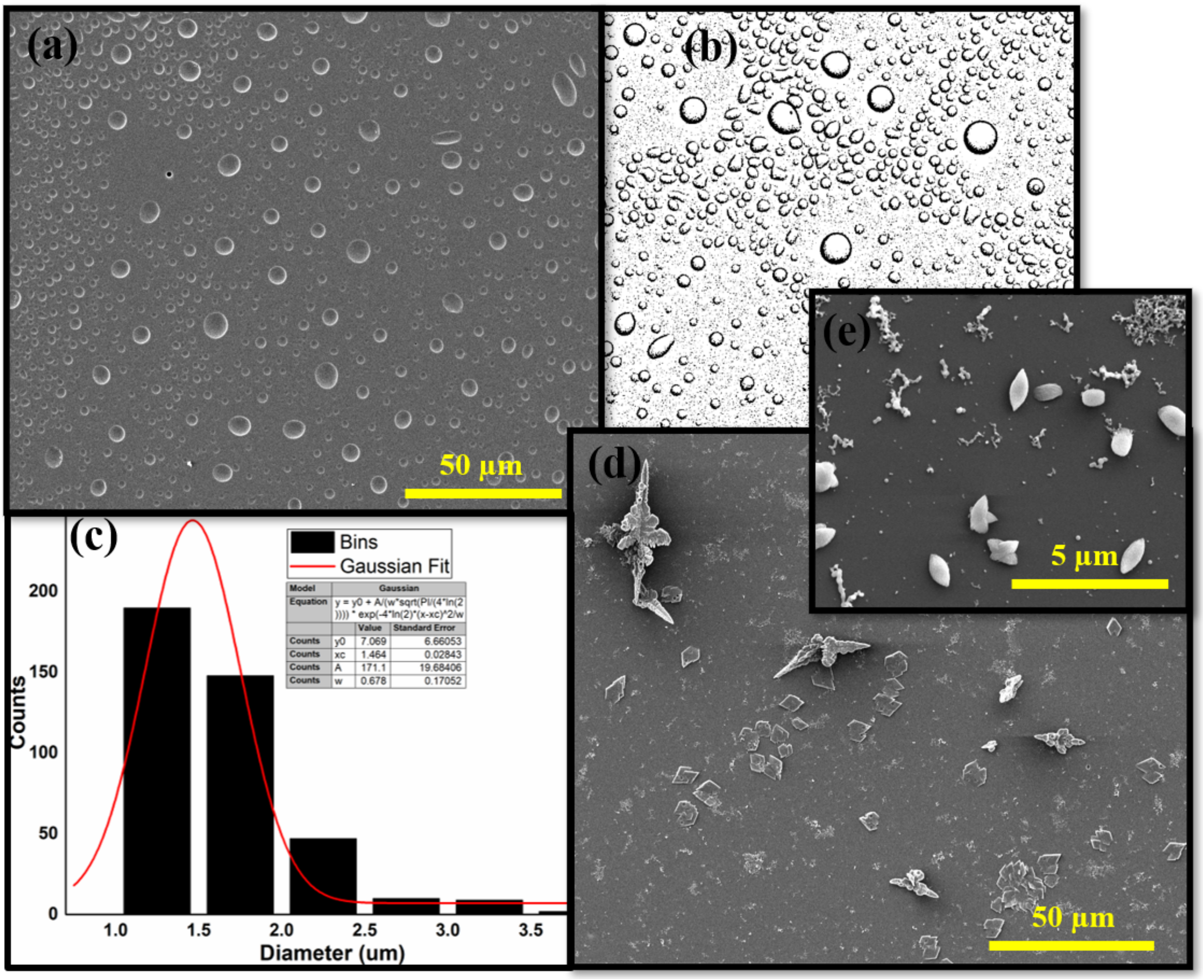

Figure S32: (a) SEM image of molecular drilled holes in the polymer at the polymer – tube interface of the thin-layer (Side A) processed in biphasic system of toluene and water (1:1) at 6500 rpm, θ = 45°, room temperature, and 30 min processing in a flat base tube, then washed with hexane, and dried under nitrogen gas, (b) ImageJ analysis of the sample identifying the drilled holes, (c) Gaussian fitted curve to the distribution of drilled holes observed in the polymer, (d) and (e) SEM images of polymer –liquid interface of the thin-layer (Side B).

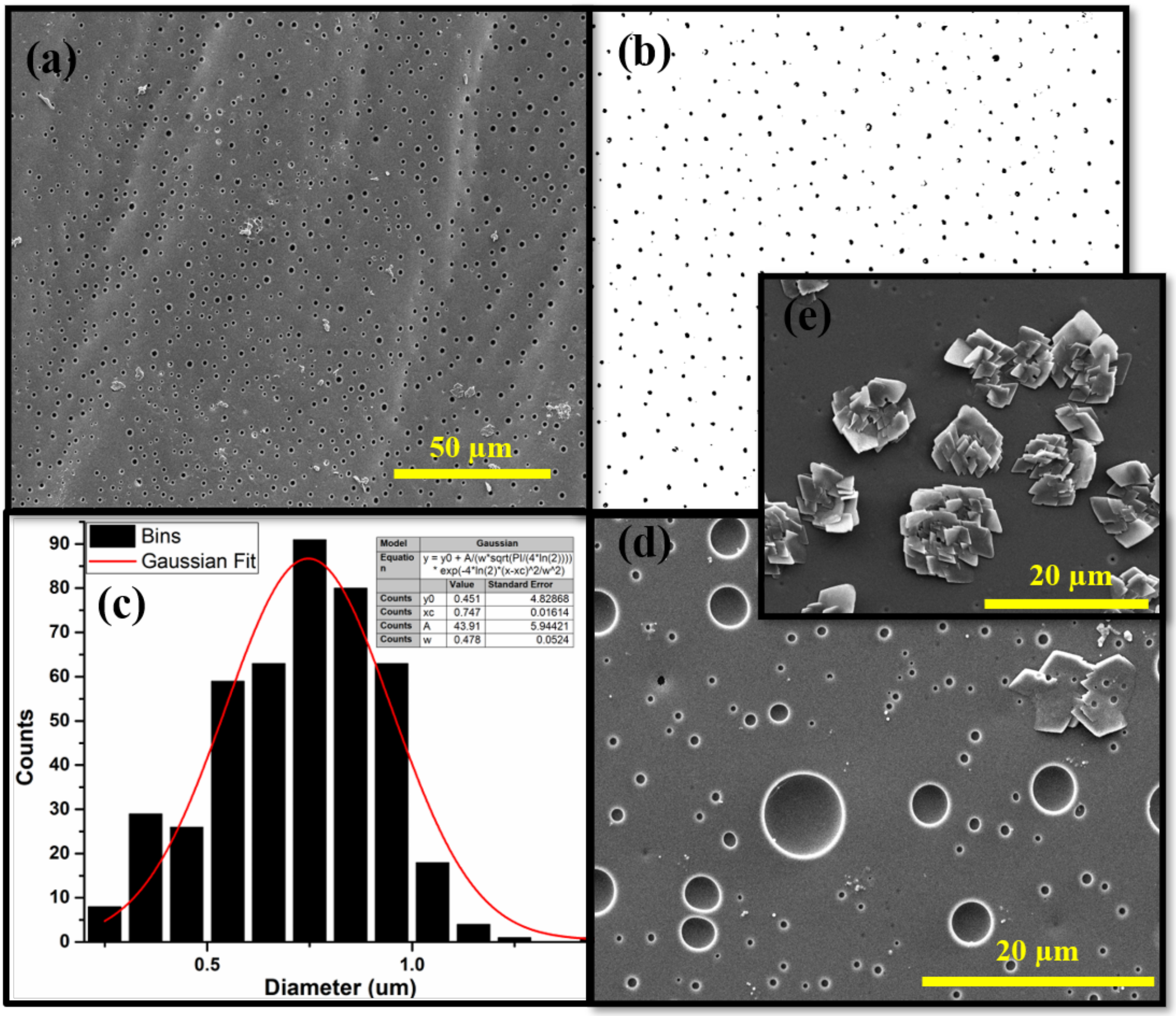

Figure S33: (a) SEM image of molecular drilled holes in the polymer at the polymer – tube interface of the thin-layer (Side A) processed in biphasic system of toluene and water (1:1) at 7000 rpm, θ = 45°, room temperature, and 30 min processing in a flat base tube, then washed with hexane, and dried under nitrogen gas, (b) ImageJ analysis of the sample identifying the drilled holes, (c) Gaussian fitted curve to the distribution of drilled holes observed in the polymer, (d) and (e) SEM images of polymer – liquid interface of the thin-layer (Side B).

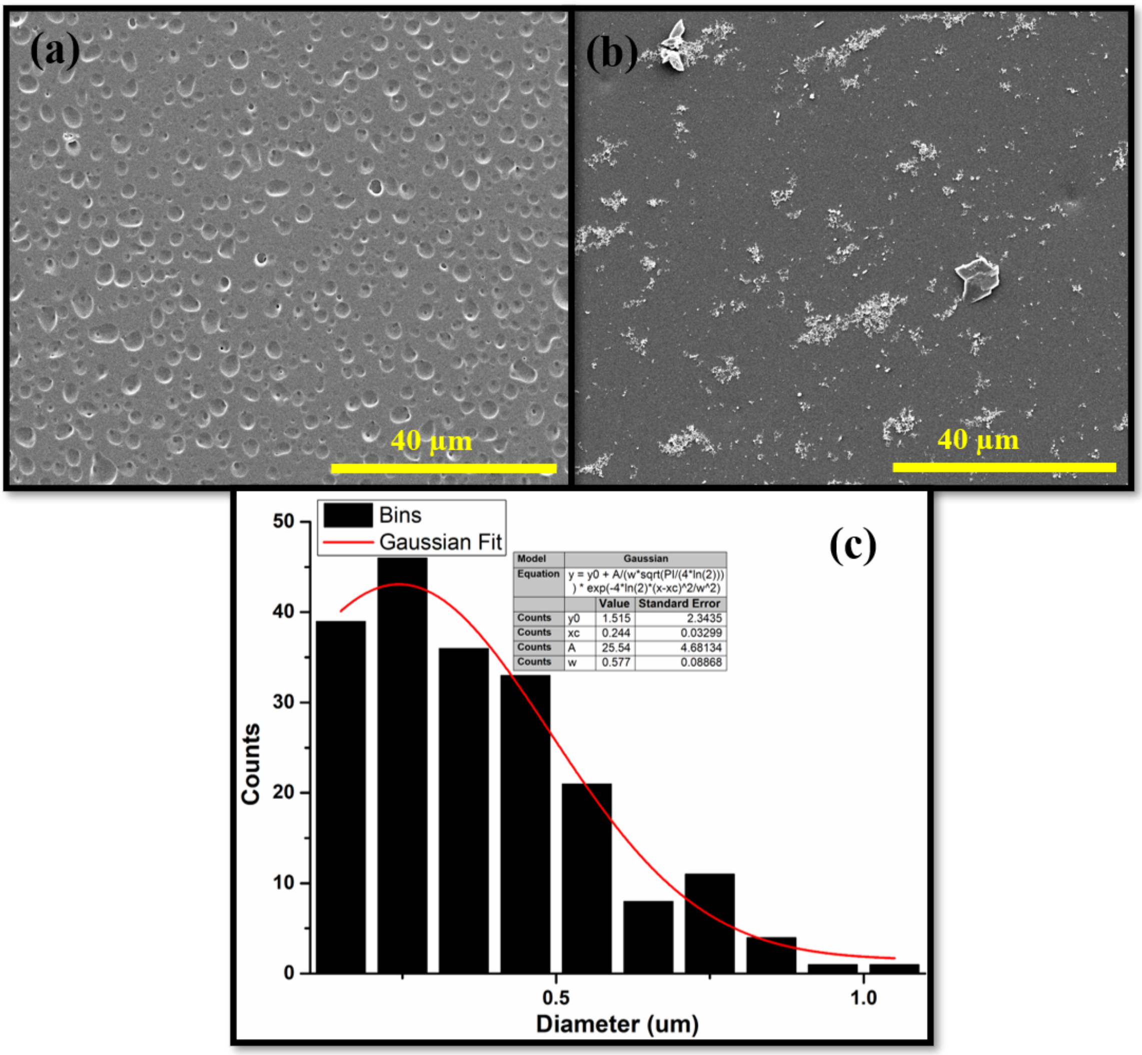

Figure S34: (a) SEM image of molecular drilled holes in the polymer at the polymer – tube interface of the thin-layer (Side A), (b) SEM image of polymer – liquid interface of the thin-film (Side B) processed in biphasic system of toluene and water (1:1), at 7500 rpm, θ = 45°, room temperature, and 30 min processing in a flat base tube, then washed with hexane, and dried under nitrogen gas, (c) Gaussian fitted curve to the distribution of drilled holes observed in the polymer at the polymer – tube interface (Side A).

S5. Liquid-liquid interfacial high mass transport

Dynamic Light Scattering (DLS)

A Malvern Zetasizer was used to determine particle size of an aqueous and organic phase (1:1) at rotational speeds, ω, 3000 rpm, 5250 rpm and 7750k rpm, tilt inclination, θ, +45° for 15 minutes processing time, in a hemispherical and flat base VFD tubes. A benchtop vortex mixer was used as a comparison to the VFD (15 minute processing time). The data was recorded in triplicates, with refractive index, η_D, water (1.3330), toluene (1.4969) and chlorobenzene (1.5248). The data was recorded immediately after processing and 24 hours after processing.

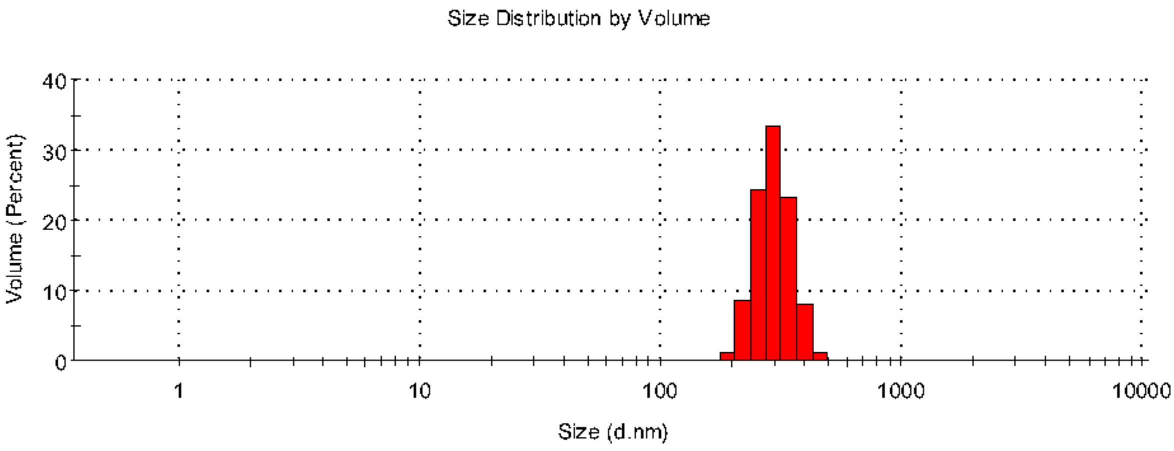

Figure S35: Histogram of the number percentage of particle size (d.nm) of a milliQ water reference.

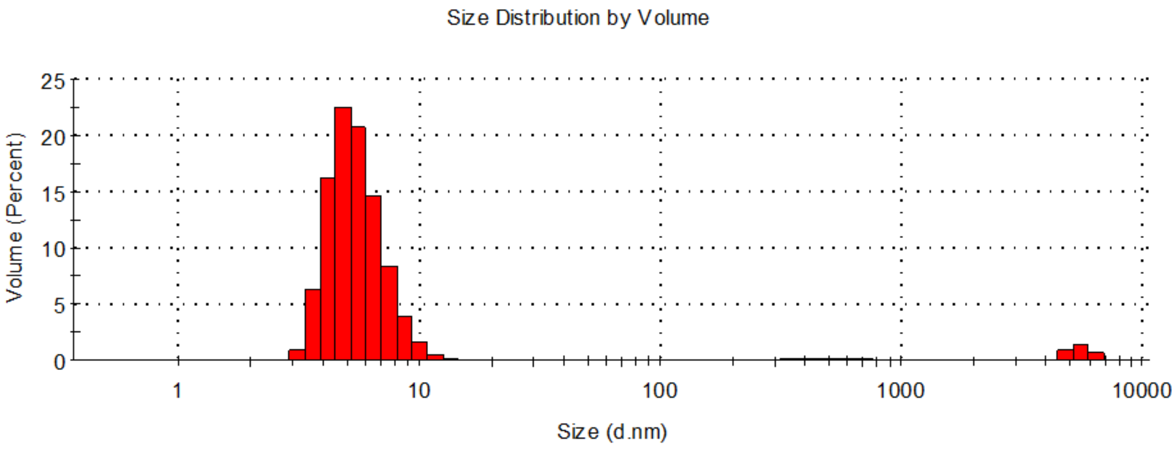

Figure S36: Histogram of the number percentage of particle size (d.nm) of a toluene (>99%) reference.

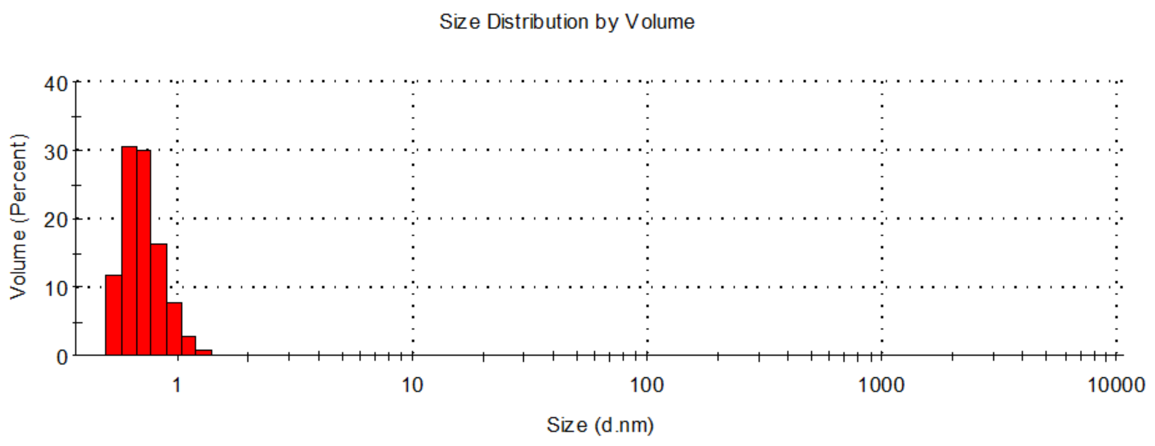

Figure S37: Histogram of the number percentage of particle size (d.nm) of a chlorobenzene (>99%) reference.

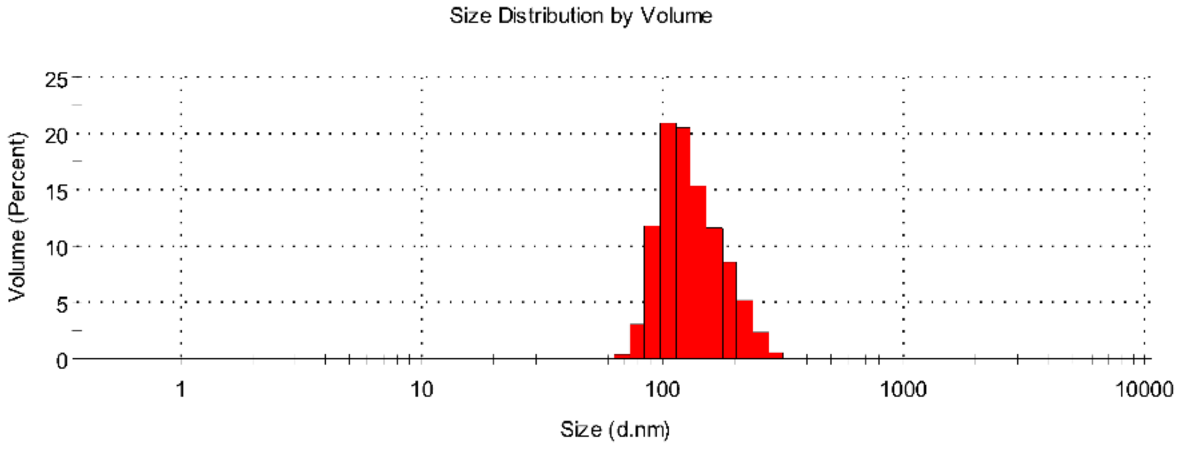

Figure 38: Histogram of the number percentage of particle size (d.nm) of water after vortex mixing with a 1:1 mixture with toluene immediately after processing.

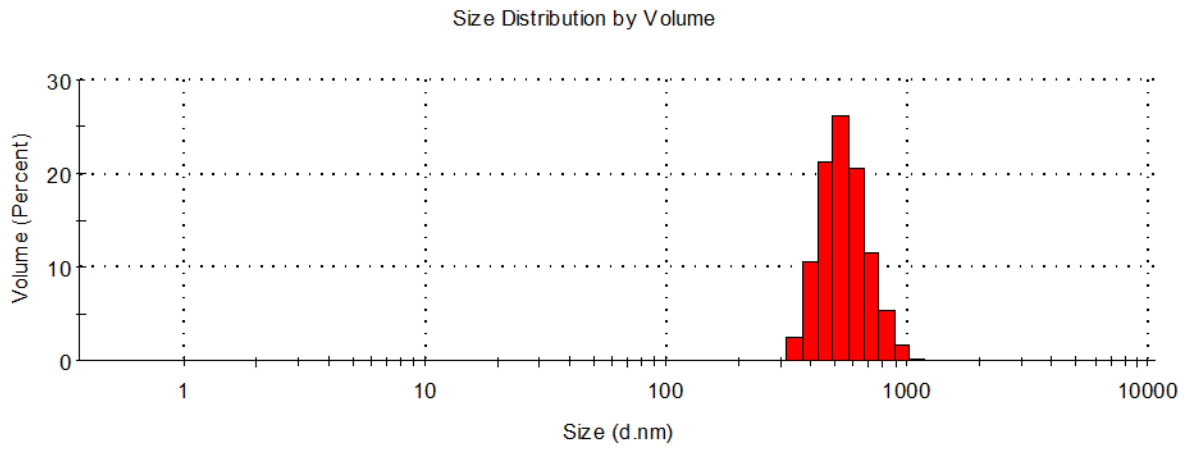

Figure S39: Histogram of the number percentage of particle size (d.nm) of water after vortex mixing with a 1:1 mixture with toluene 24 hours after processing.

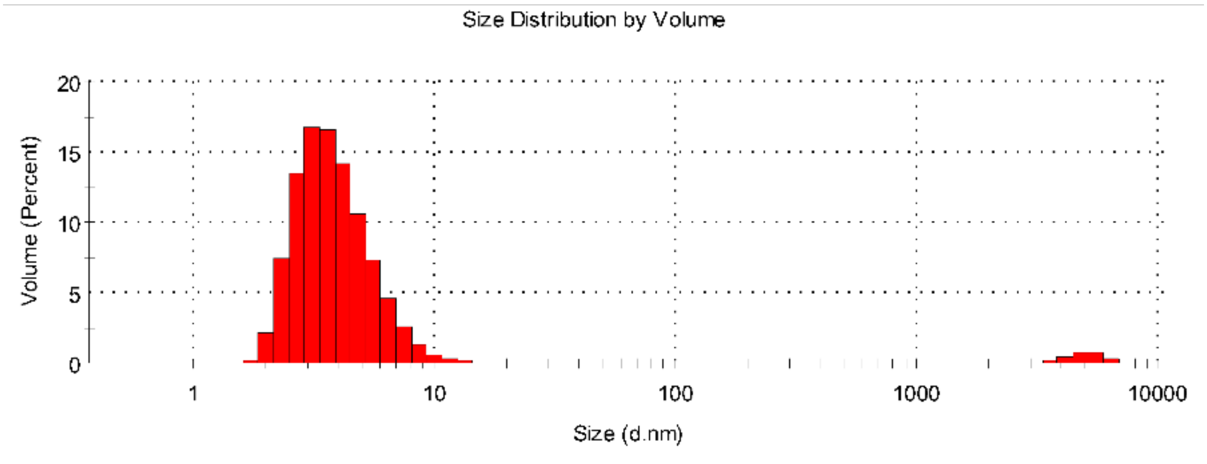

Figure S40: Histogram of the number percentage of particle size (d.nm) of toluene after vortex mixing with a 1:1 mixture with water, immediately after processing.

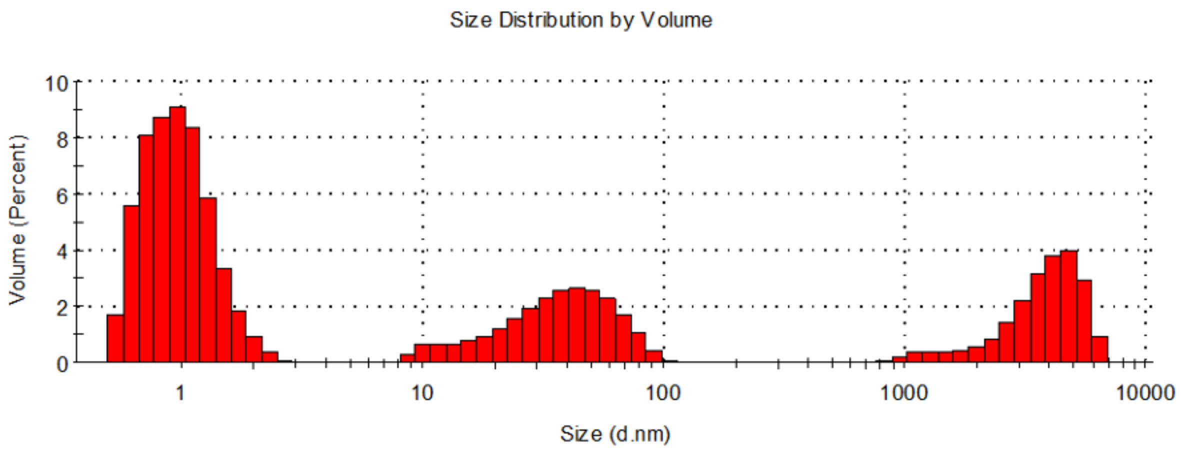

Figure S41: Histogram of the number percentage of particle size (d.nm) of toluene after vortex mixing with a 1:1 mixture with water, 24 hours after processing.

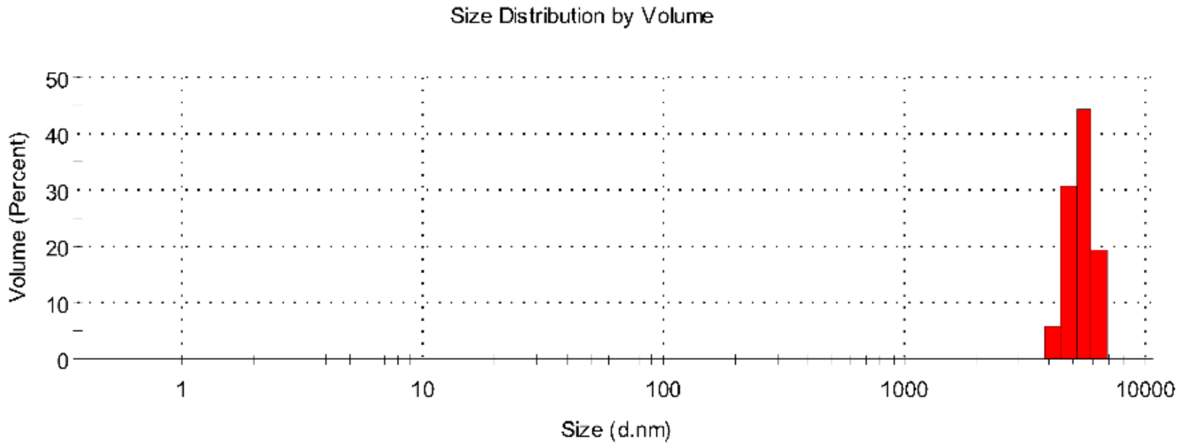

Figure S42: Histogram of the number percentage of particle size (d.nm) of water after vortex mixing with a 1:1 mixture with chlorobenzene immediately after processing.

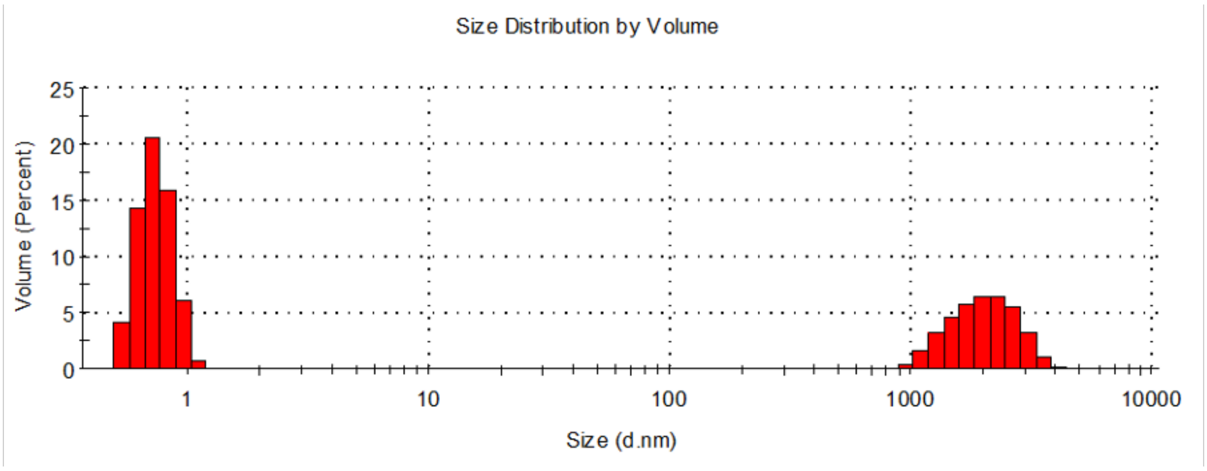

Figure S43: Histogram of the number percentage of particle size (d.nm) of water after vortex mixing with a 1:1 mixture with chlorobenzene 24 hours after processing.

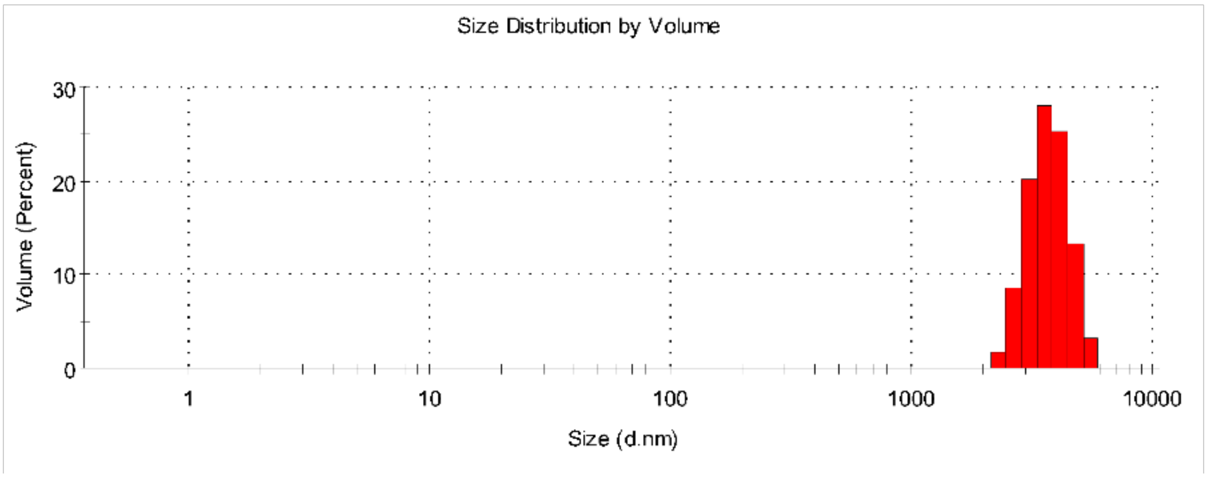

Figure S44: Histogram of the number percentage of particle size (d.nm) of chlorobenzene after vortex mixing with a 1:1 mixture with water, immediately after processing.

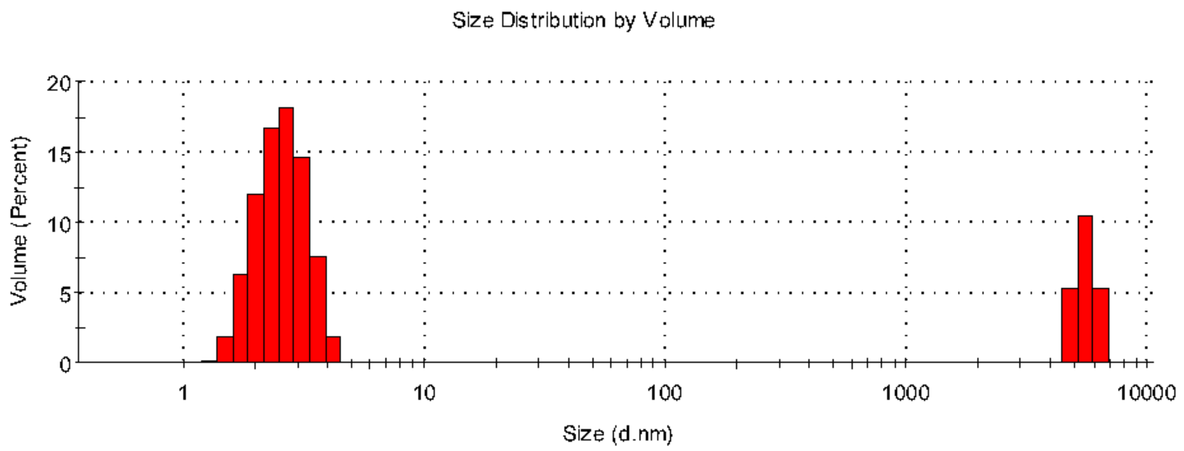

Figure S45: Histogram of the number percentage of particle size (d.nm) of chlorobenzene after vortex mixing with a 1:1 mixture with water, 24 hours after processing.

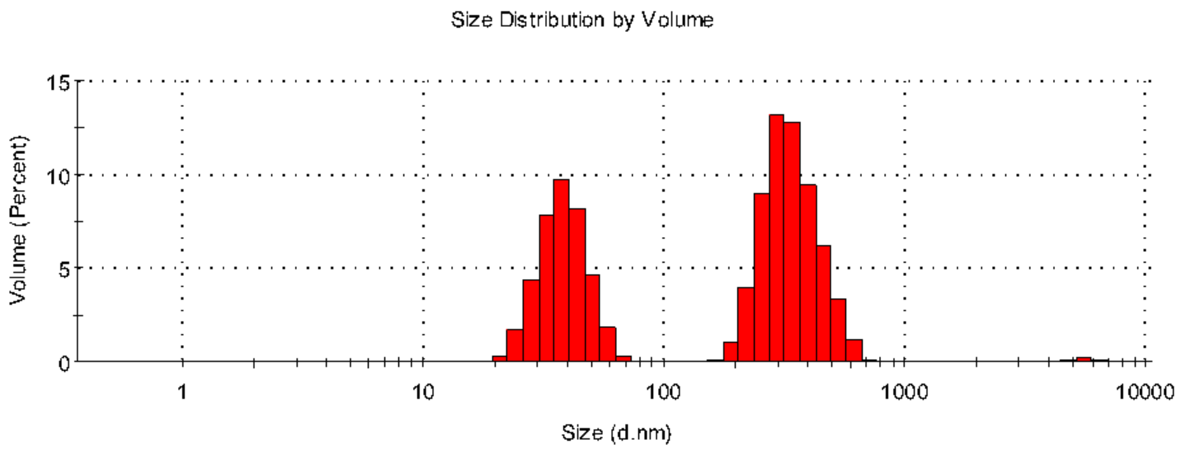

Figure S46: Histogram of the number percentage of particle size (d.nm) of water after processing in the VFD, hemispherical base VFD tube (20 mm OD, 17.5 mm ID, 18.5 cm in length), ω, 3000 rpm, tilt inclination, θ, +45° for 15 minutes processing time with a 1:1 mixture with chlorobenzene, immediately after processing.

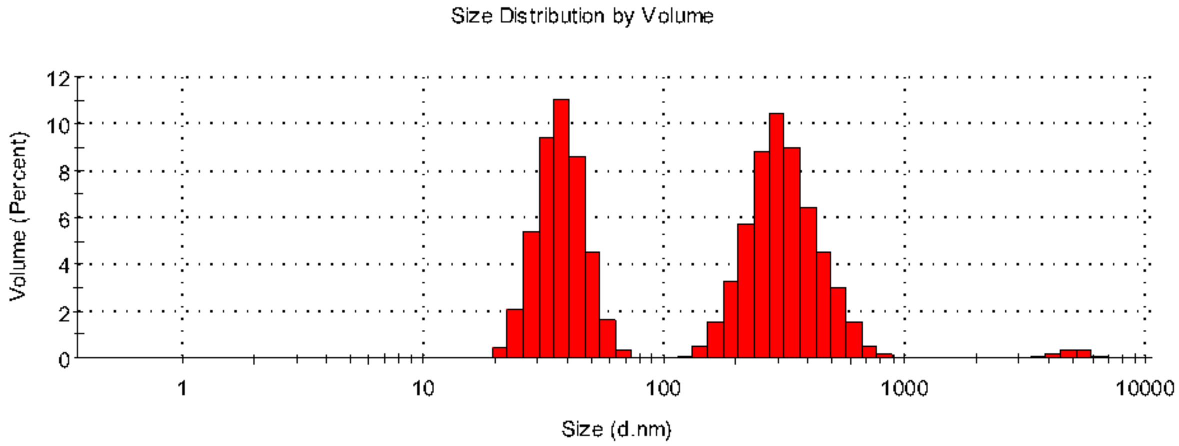

Figure S47: Histogram of the number percentage of particle size (d.nm) of water after processing in the VFD, hemispherical base VFD tube (20 mm OD, 17.5 mm ID, 18.5 cm in length), ω, 3000 rpm, tilt inclination, θ, +45° for 15 minutes processing time with a 1:1 mixture with chlorobenzene, 24 hours after processing.

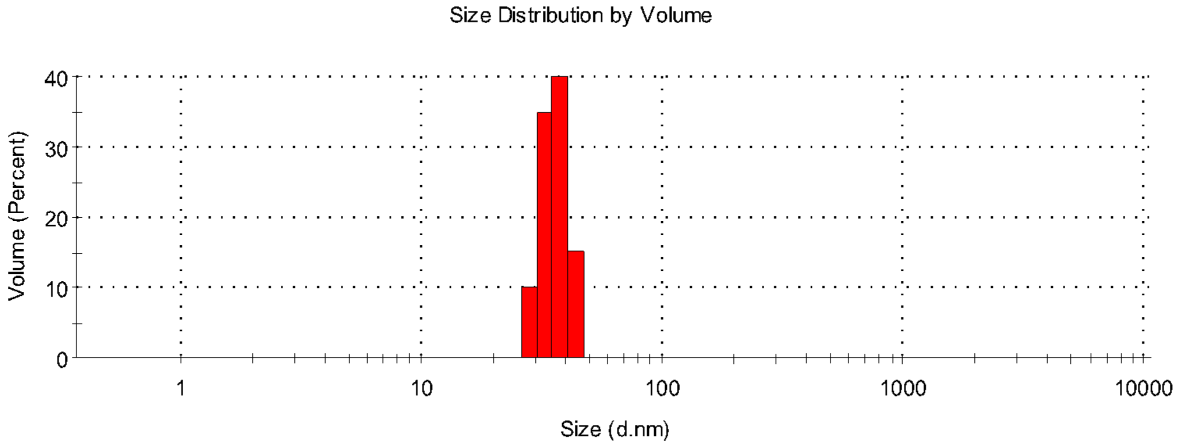

Figure S4.14: Histogram of the number percentage of particle size (d.nm) of chlorobenzene after processing in the VFD, hemispherical base VFD tube (20 mm OD, 17.5 mm ID, 18.5 cm in length), ω, 3000 rpm, tilt inclination, θ, +45° for 15 minutes processing time with a 1:1 mixture with water, immediately after processing.

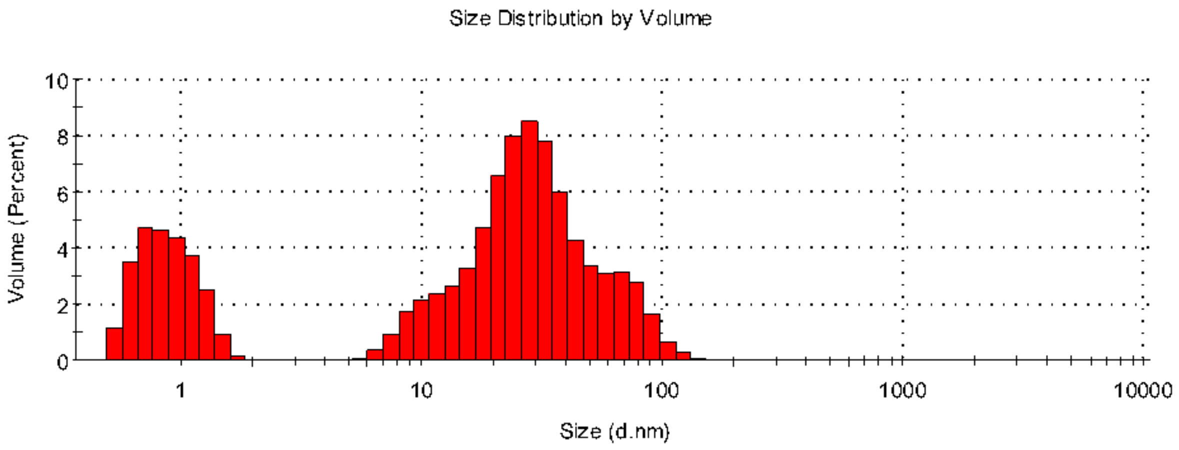

Figure S48: Histogram of the number percentage of particle size (d.nm) of chlorobenzene after processing in the VFD, hemispherical base VFD tube (20 mm OD, 17.5 mm ID, 18.5 cm in length), ω, 3000 rpm, tilt inclination, θ, +45° for 15 minutes processing time with a 1:1 mixture with water, 24 hours after processing.

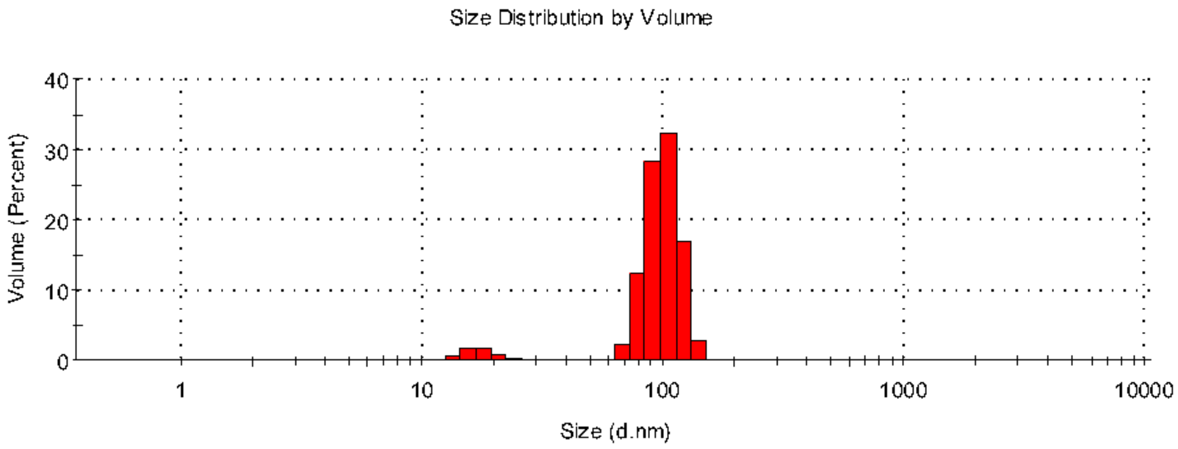

Figure S49: Histogram of the number percentage of particle size (d.nm) of water after processing in the VFD, hemispherical base VFD tube (20 mm OD, 17.5 mm ID, 18.5 cm in length), ω, 5250 rpm, tilt inclination, θ, +45° for 15 minutes processing time with a 1:1 mixture with chlorobenzene, immediately after processing.

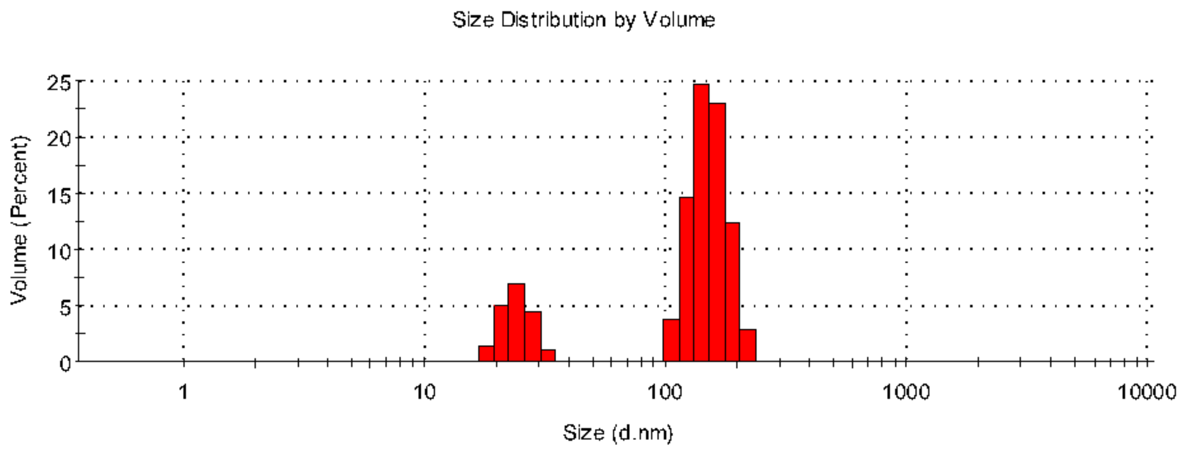

Figure S50: Histogram of the number percentage of particle size (d.nm) of water after processing in the VFD, hemispherical base VFD tube (20 mm OD, 17.5 mm ID, 18.5 cm in length), ω, 5250 rpm, tilt inclination, θ, +45° for 15 minutes processing time with a 1:1 mixture with chlorobenzene, 24 hours after processing.

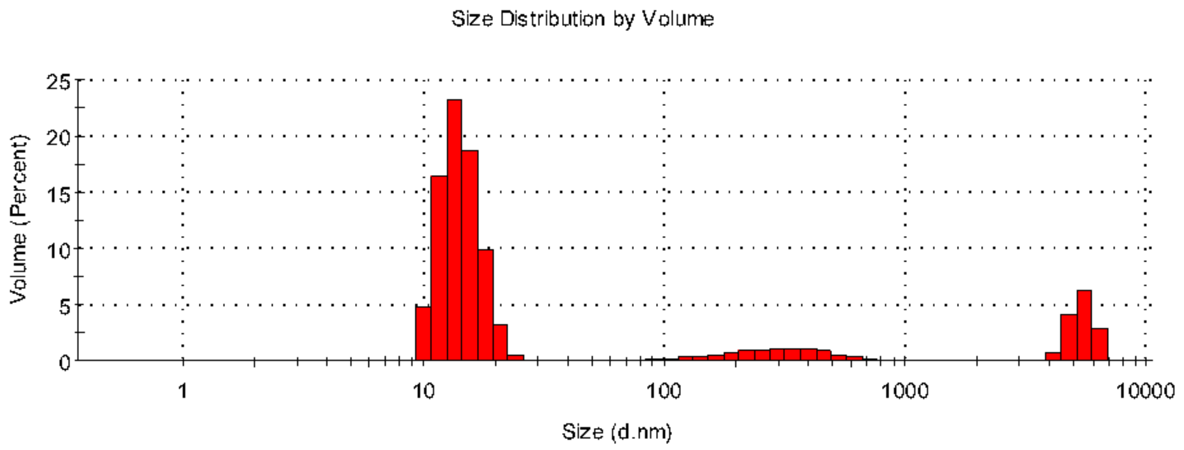

Figure S4.18: Histogram of the number percentage of particle size (d.nm) of chlorobenzene after processing in the VFD, hemispherical base VFD tube (20 mm OD, 17.5 mm ID, 18.5 cm in length), ω, 5250 rpm, tilt inclination, θ, +45° for 15 minutes processing time with a 1:1 mixture with water, immediately after processing.

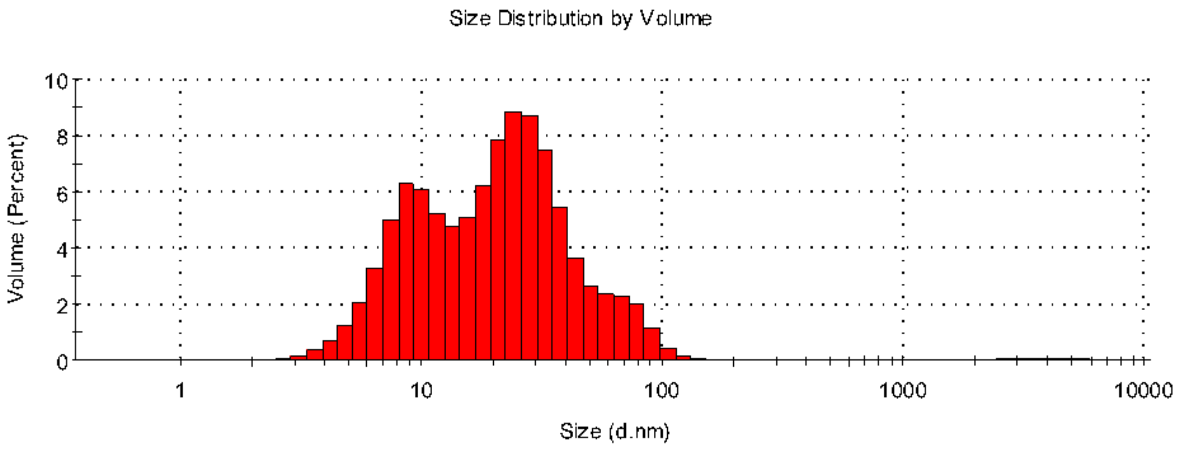

Figure S51: Histogram of the number percentage of particle size (d.nm) of chlorobenzene after processing in the VFD, hemispherical base VFD tube (20 mm OD, 17.5 mm ID, 18.5 cm in length), ω, 5250 rpm, tilt inclination, θ, +45° for 15 minutes processing time with a 1:1 mixture with water, 24 hours after processing.

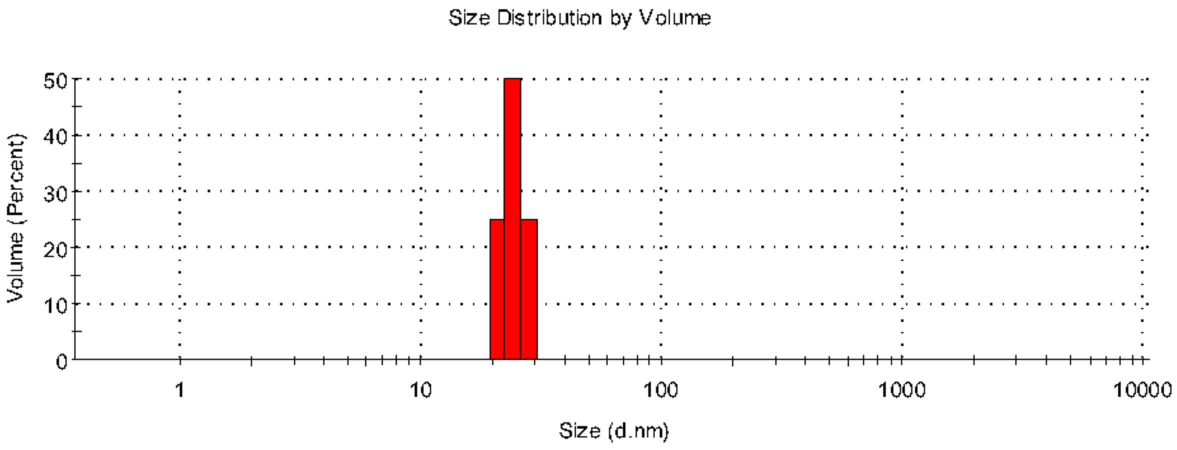

Figure S52: Histogram of the number percentage of particle size (d.nm) of water after processing in the VFD, hemispherical base VFD tube (20 mm OD, 17.5 mm ID, 18.5 cm in length), ω, 7500 rpm, tilt inclination, θ, +45° for 15 minutes processing time with a 1:1 mixture with chlorobenzene, immediately after processing.

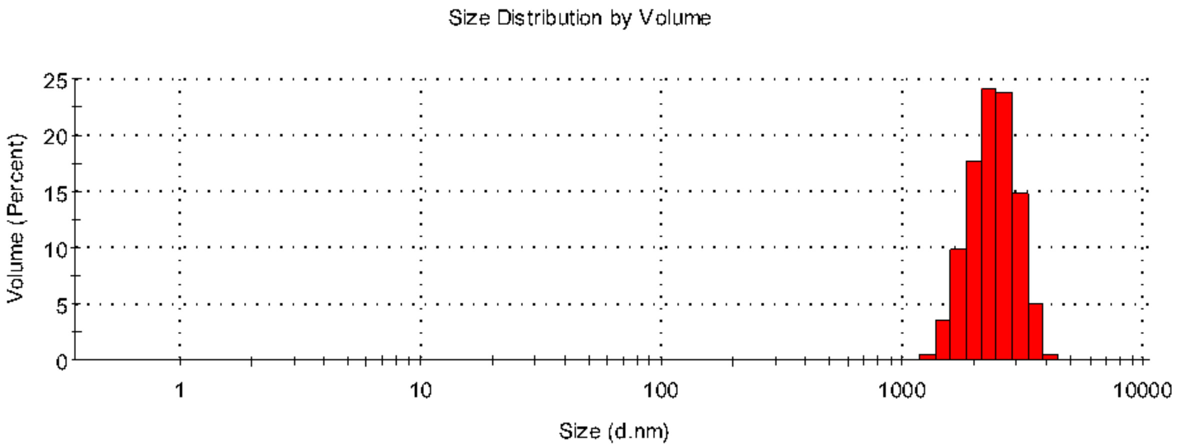

Figure S53: Histogram of the number percentage of particle size (d.nm) of water after processing in the VFD, hemispherical base VFD tube (20 mm OD, 17.5 mm ID, 18.5 cm in length), ω, 7500 rpm, tilt inclination, θ, +45° for 15 minutes processing time with a 1:1 mixture with chlorobenzene, 24 hours after processing.

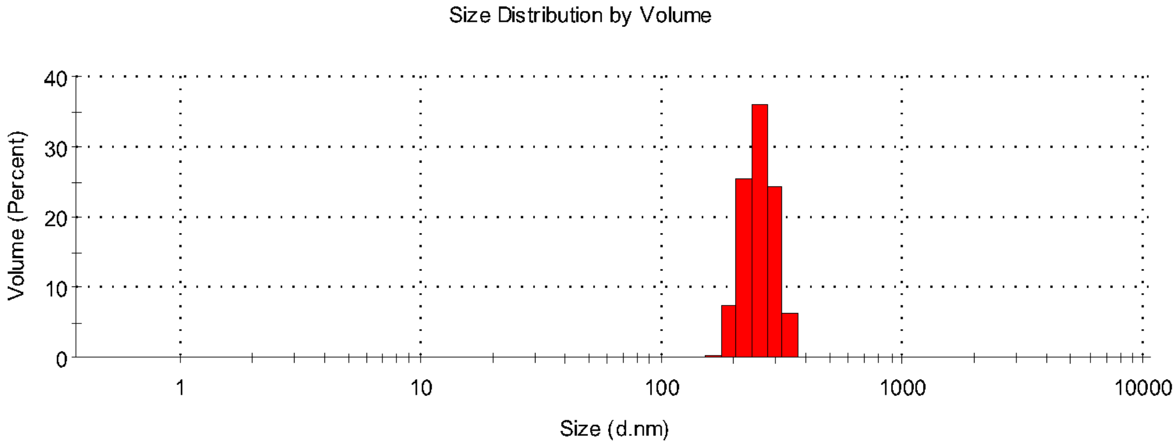

Figure S54: Histogram of the number percentage of particle size (d.nm) of chlorobenzene after processing in the VFD, hemispherical base VFD tube (20 mm OD, 17.5 mm ID, 18.5 cm in length), ω, 7500 rpm, tilt inclination, θ, +45° for 15 minutes processing time with a 1:1 mixture with water, immediately after processing.

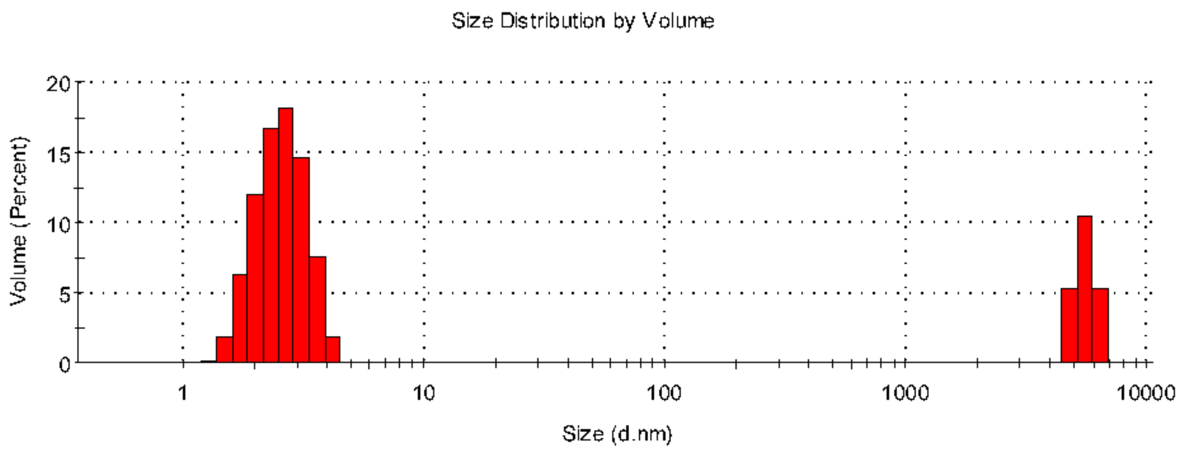

Figure S55: Histogram of the number percentage of particle size (d.nm) of chlorobenzene after processing in the VFD, hemispherical base VFD tube (20 mm OD, 17.5 mm ID, 18.5 cm in length), ω, 7500 rpm, tilt inclination, θ, +45° for 15 minutes processing time with a 1:1 mixture with water, 24 hours after processing.

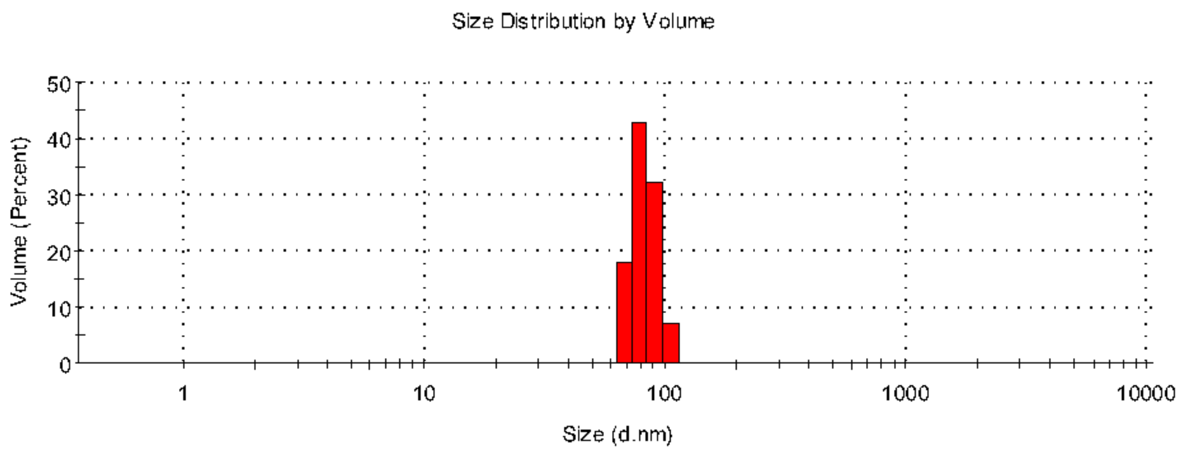

Figure S56: Histogram of the number percentage of particle size (d.nm) of water after processing in the VFD, flat base VFD tube (20 mm OD, 17.5 mm ID, 18.5 cm in length), ω, 3000 rpm, tilt inclination, θ, +45° for 15 minutes processing time with a 1:1 mixture with toluene, immediately after processing.

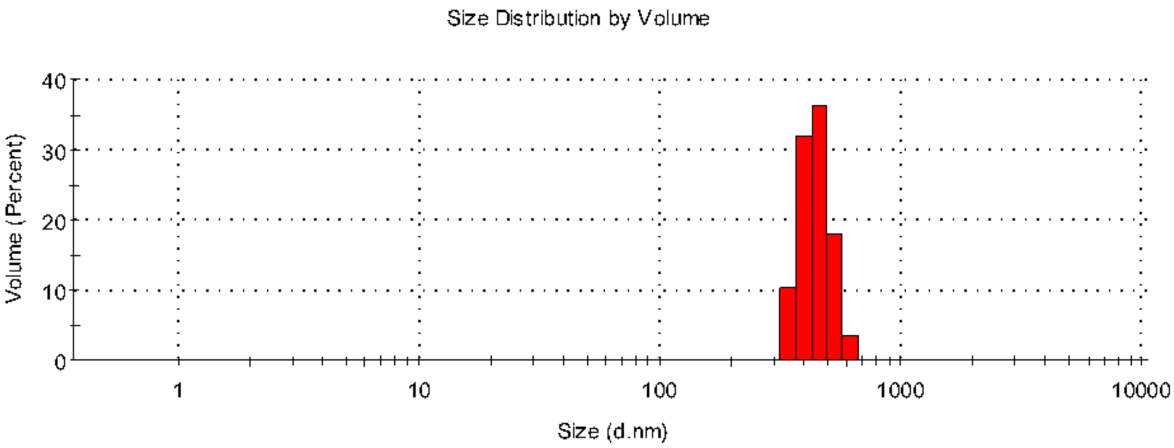

Figure S58: Histogram of the number percentage of particle size (d.nm) of water after processing in the VFD, flat base VFD tube (20 mm OD, 17.5 mm ID, 18.5 cm in length), ω, 3000 rpm, tilt inclination, θ, +45° for 15 minutes processing time with a 1:1 mixture with toluene, 24 hours after processing.

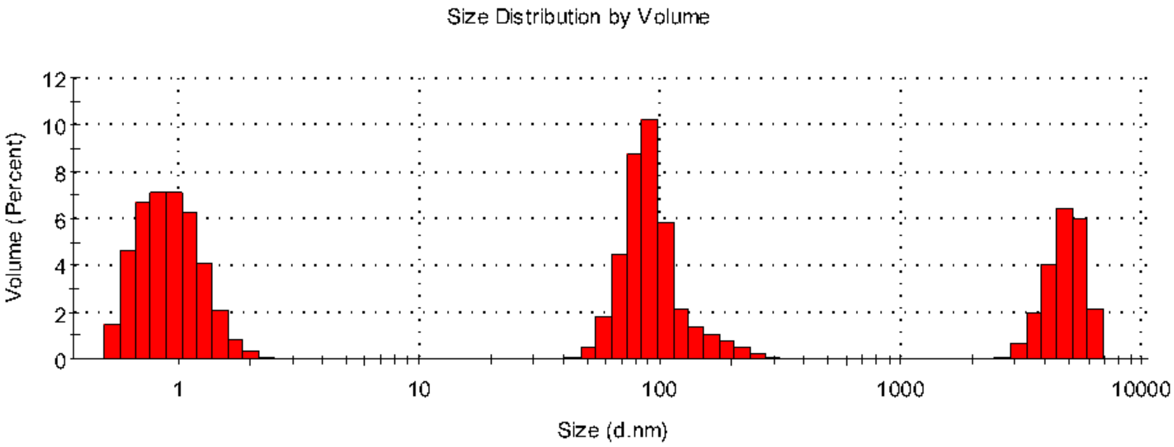

Figure S59: Histogram of the number percentage of particle size (d.nm) of toluene after processing in the VFD, flat base VFD tube (20 mm OD, 17.5 mm ID, 18.5 cm in length), ω, 3000 rpm, tilt inclination, θ, +45° for 15 minutes processing time with a 1:1 mixture with water, immediately after processing.

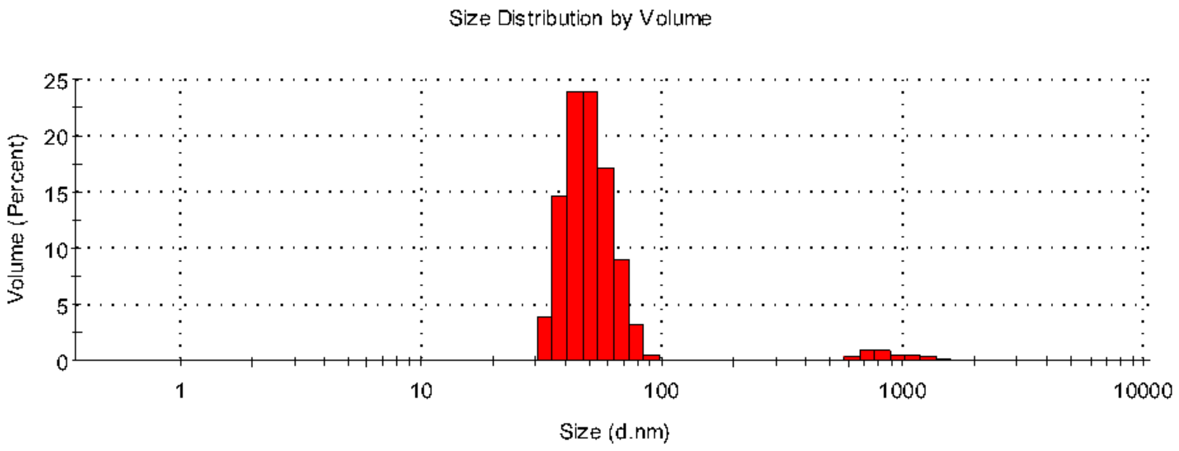

Figure S60: Histogram of the number percentage of particle size (d.nm) of toluene after processing in the VFD, flat base VFD tube (20 mm OD, 17.5 mm ID, 18.5 cm in length), ω, 3000 rpm, tilt inclination, θ, +45° for 15 minutes processing time with a 1:1 mixture with water, 24 hours after processing.

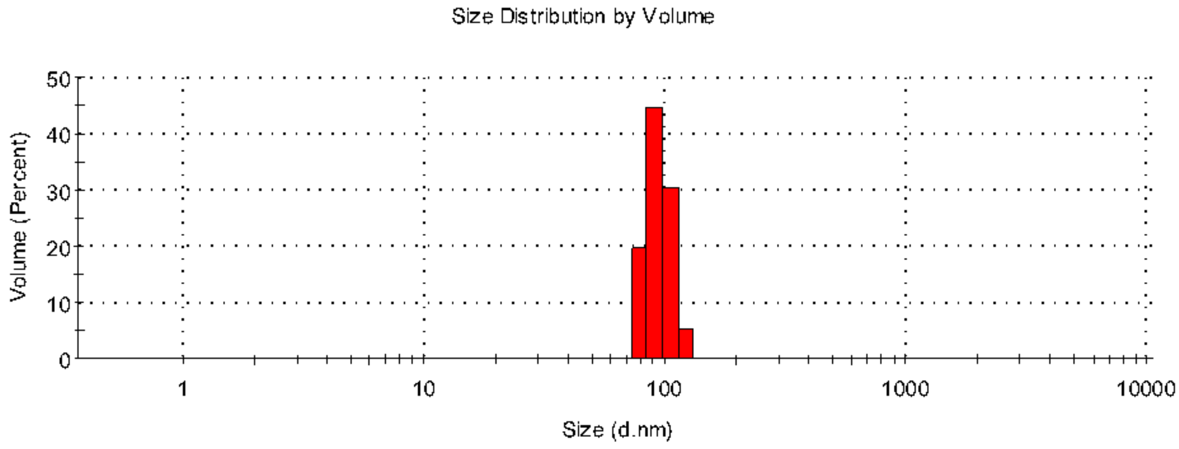

Figure S61: Histogram of the number percentage of particle size (d.nm) of water after processing in the VFD, flat base VFD tube (20 mm OD, 17.5 mm ID, 18.5 cm in length), ω, 5250 rpm, tilt inclination, θ, +45° for 15 minutes processing time with a 1:1 mixture with toluene, immediately after processing.

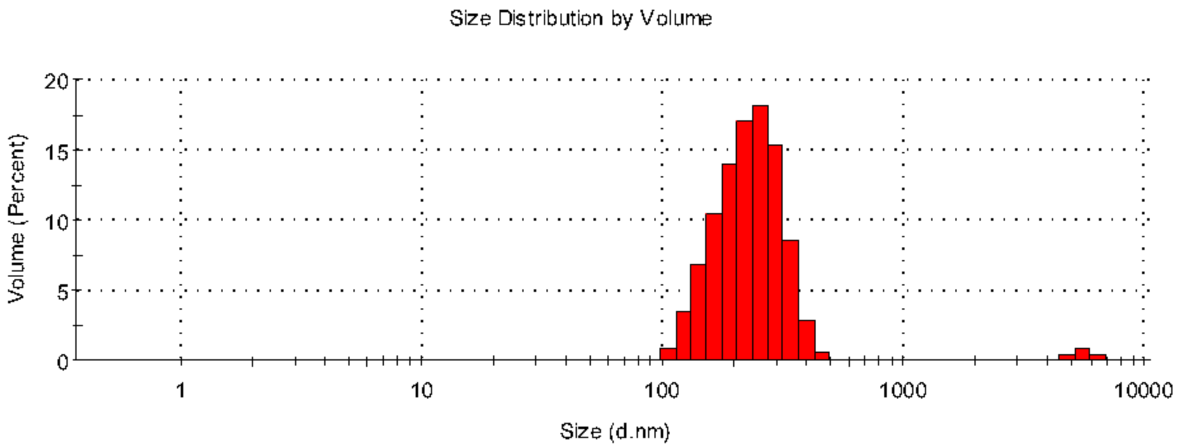

Figure S62: Histogram of the number percentage of particle size (d.nm) of water after processing in the VFD, flat base VFD tube (20 mm OD, 17.5 mm ID, 18.5 cm in length), ω, 5250 rpm, tilt inclination, θ, +45° for 15 minutes processing time with a 1:1 mixture with toluene, 24 hours after processing.

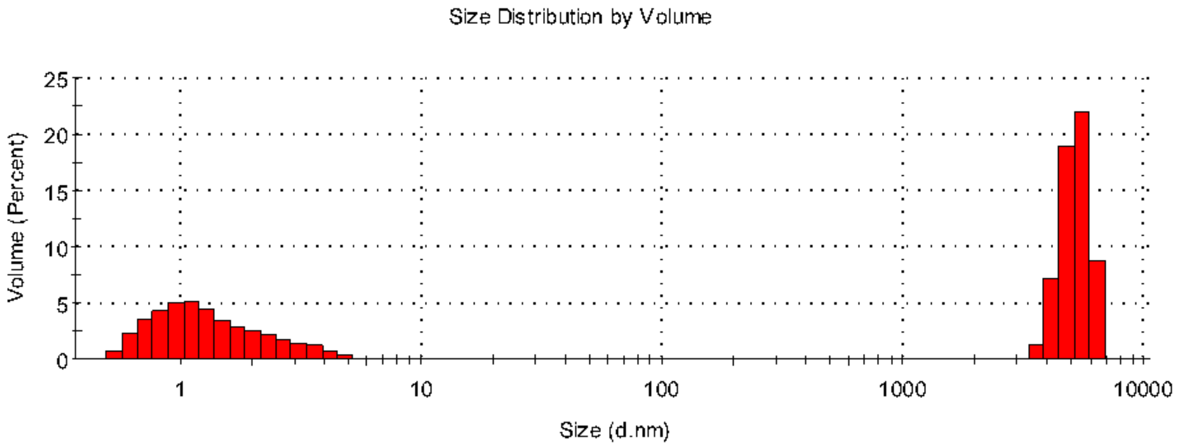

Figure S63: Histogram of the number percentage of particle size (d.nm) of toluene after processing in the VFD, flat base, ω, 5250 rpm, tilt inclination, θ, +45° for 15 minutes processing time with a 1:1 mixture with water, immediately after processing.

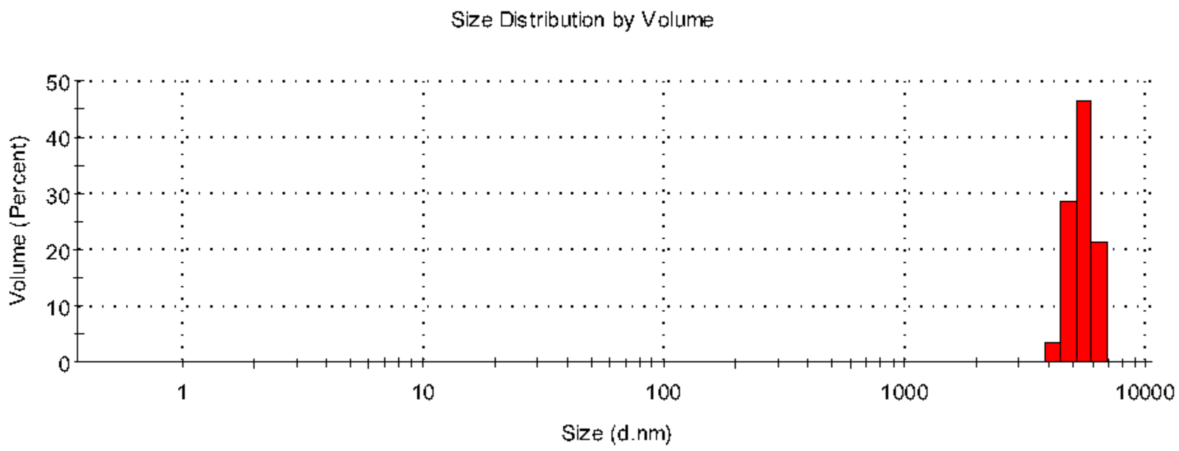

Figure S64: Histogram of the number percentage of particle size (d.nm) of toluene after processing in the VFD, flat base VFD tube (20 mm OD, 17.5 mm ID, 18.5 cm in length), ω, 5250 rpm, tilt inclination, θ, +45° for 15 minutes processing time with a 1:1 mixture with water, 24 hours after processing.

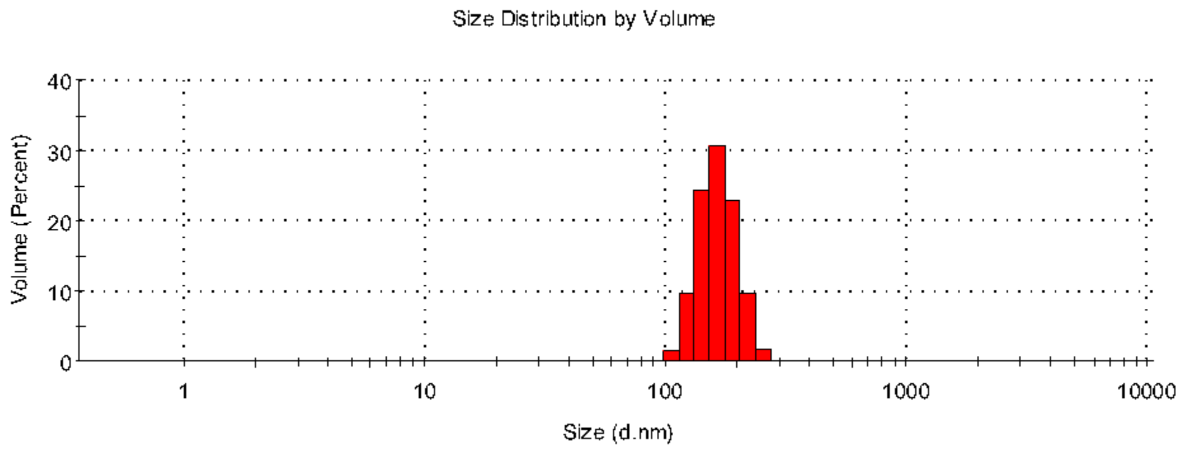

Figure S65: Histogram of the number percentage of particle size (d.nm) of water after processing in the VFD, flat base VFD tube (20 mm OD, 17.5 mm ID, 18.5 cm in length), ω, 7500 rpm, tilt inclination, θ, +45° for 15 minutes processing time with a 1:1 mixture with toluene, immediately after processing.

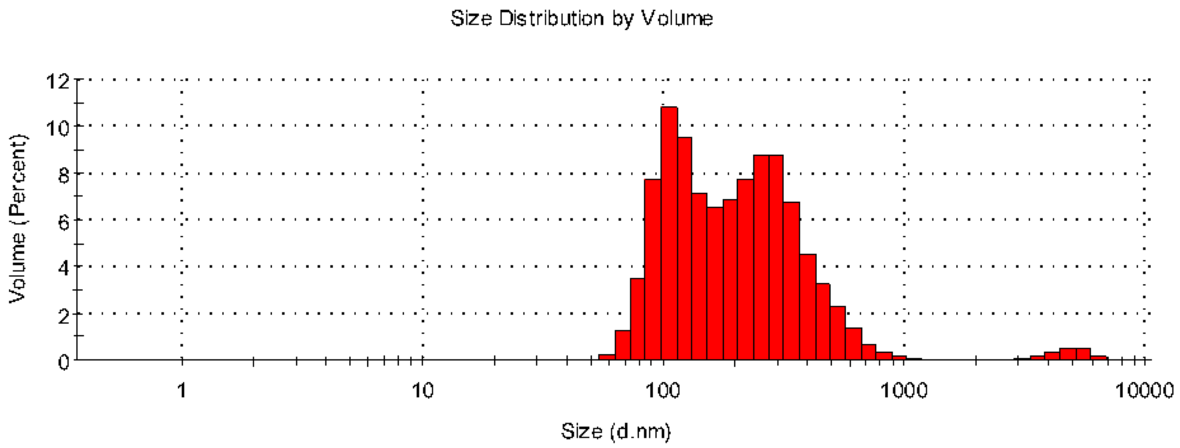

Figure S66: Histogram of the number percentage of particle size (d.nm) of water after processing in the VFD, flat base VFD tube (20 mm OD, 17.5 mm ID, 18.5 cm in length), ω, 7500 rpm, tilt inclination, θ, +45° for 15 minutes processing time with a 1:1 mixture with toluene, 24 hours after processing.

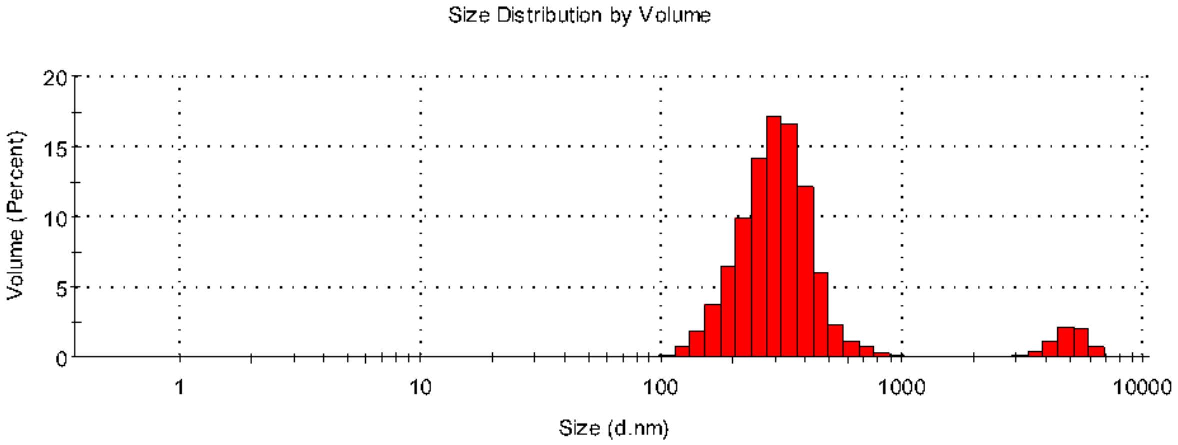

Figure S67: Histogram of the number percentage of particle size (d.nm) of toluene after processing in the VFD, flat base VFD tube (20 mm OD, 17.5 mm ID, 18.5 cm in length), ω, 7500 rpm, tilt inclination, θ, +45° for 15 minutes processing time with a 1:1 mixture with water, immediately after processing.

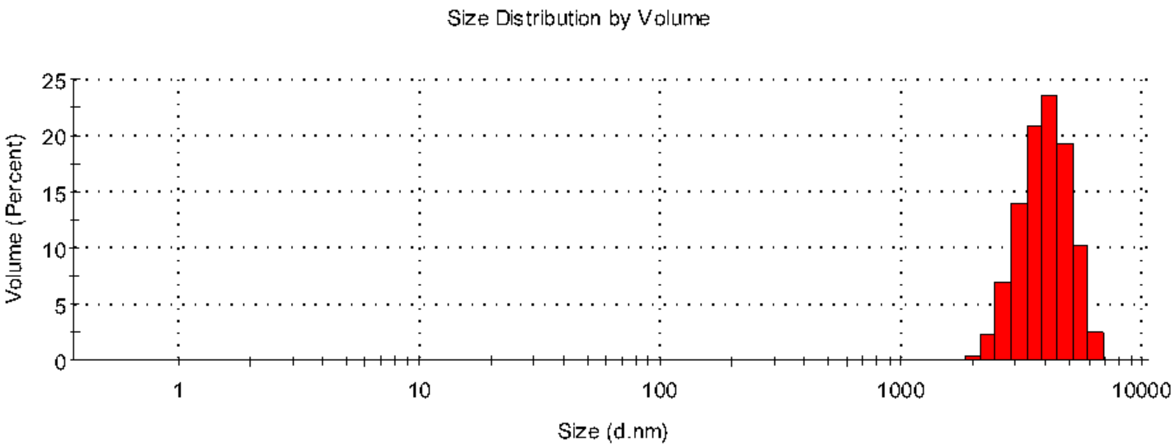

Figure S68: Histogram of the number percentage of particle size (d.nm) of toluene after processing in the VFD, flat base VFD tube (20 mm OD, 17.5 mm ID, 18.5 cm in length), ω, 7500 rpm, tilt inclination, θ, +45° for 15 minutes processing time with a 1:1 mixture with water, 24 hours after processing.

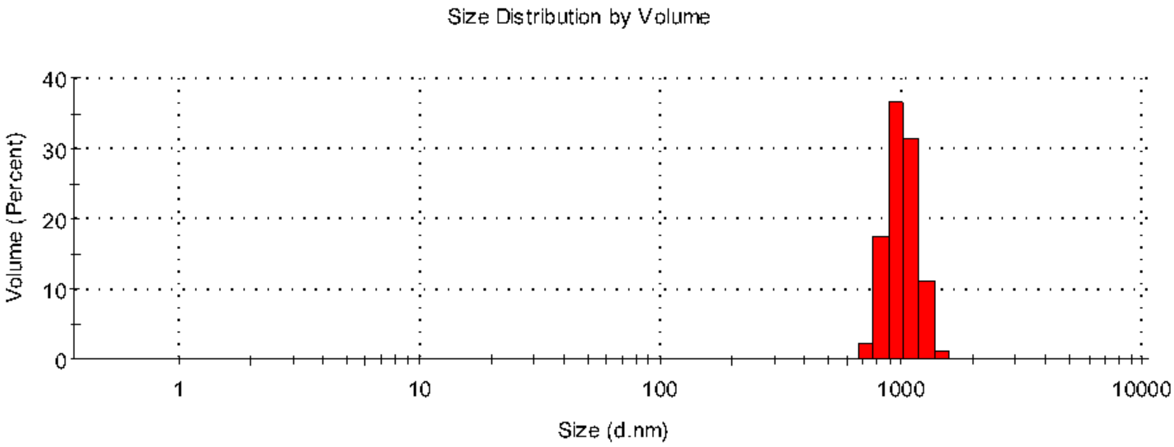

Figure S69: Histogram of the number percentage of particle size (d.nm) of water after processing in the VFD, flat base VFD tube (20 mm OD, 17.5 mm ID, 18.5 cm in length), ω, 3000 rpm, tilt inclination, θ, +45° for 15 minutes processing time with a 1:1 mixture with chlorobenzene immediately after processing.

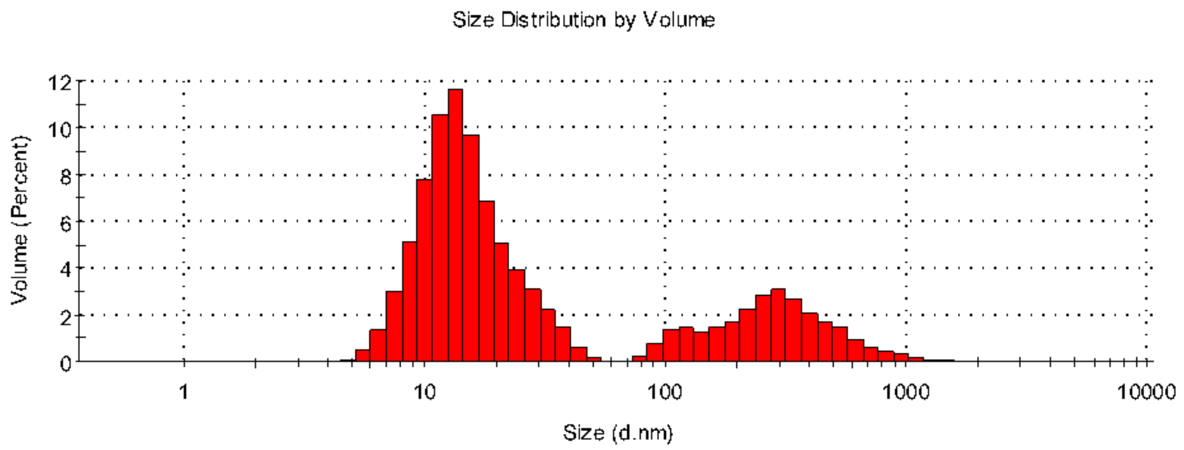

Figure S70: Histogram of the number percentage of particle size (d.nm) of water after processing in the VFD, flat base VFD tube (20 mm OD, 17.5 mm ID, 18.5 cm in length), ω, 3000 rpm, tilt inclination, θ, +45° for 15 minutes processing time with a 1:1 mixture with chlorobenzene, 24 hours after processing.

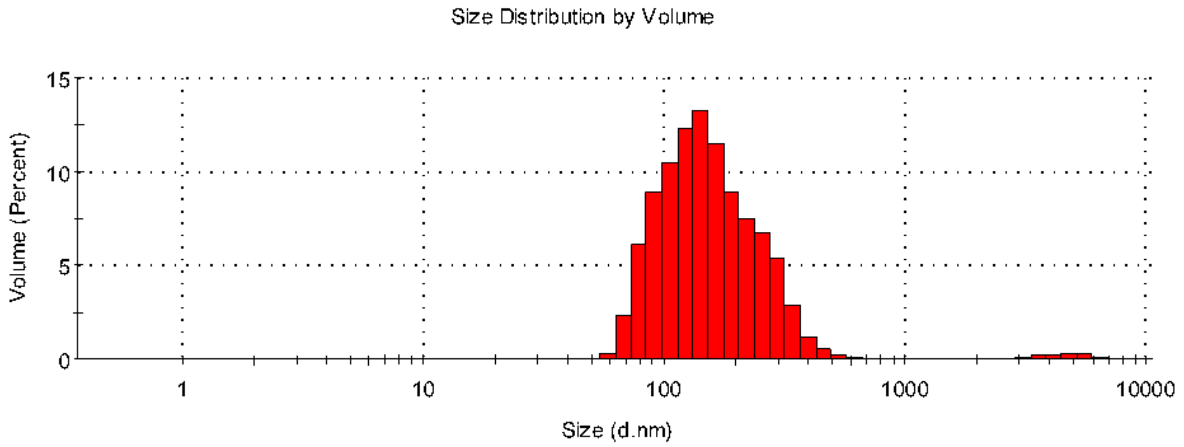

Figure S71: Histogram of the number percentage of particle size (d.nm) of chlorobenzene after processing in the VFD, flat base VFD tube (20 mm OD, 17.5 mm ID, 18.5 cm in length), ω, 3000 rpm, tilt inclination, θ, +45° for 15 minutes processing time with a 1:1 mixture with water, immediately after processing.

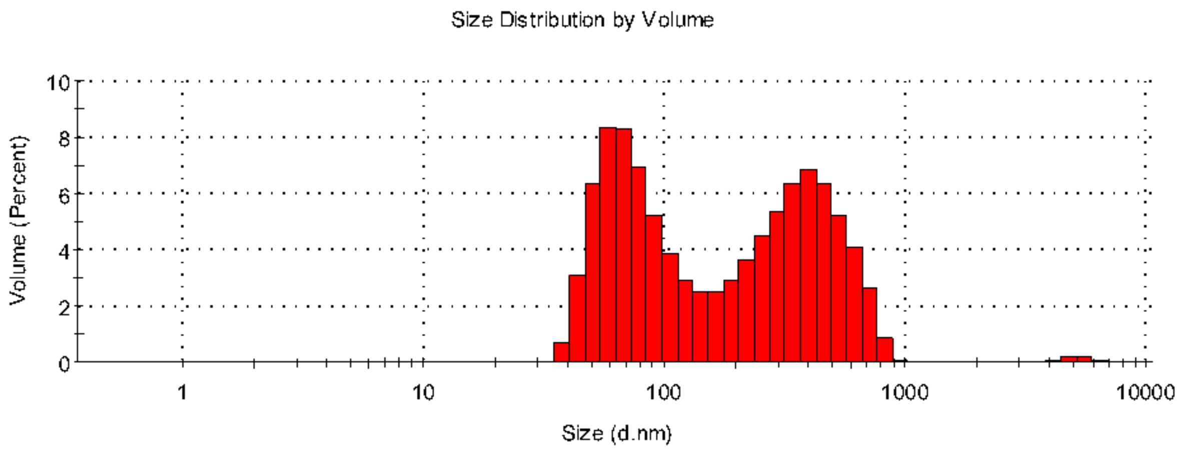

Figure S72: Histogram of the number percentage of particle size (d.nm) of chlorobenzene after processing in the VFD, flat base VFD tube (20 mm OD, 17.5 mm ID, 18.5 cm in length), ω, 3000 rpm, tilt inclination, θ, +45° for 15 minutes processing time with a 1:1 mixture with water, 24 hours after processing.

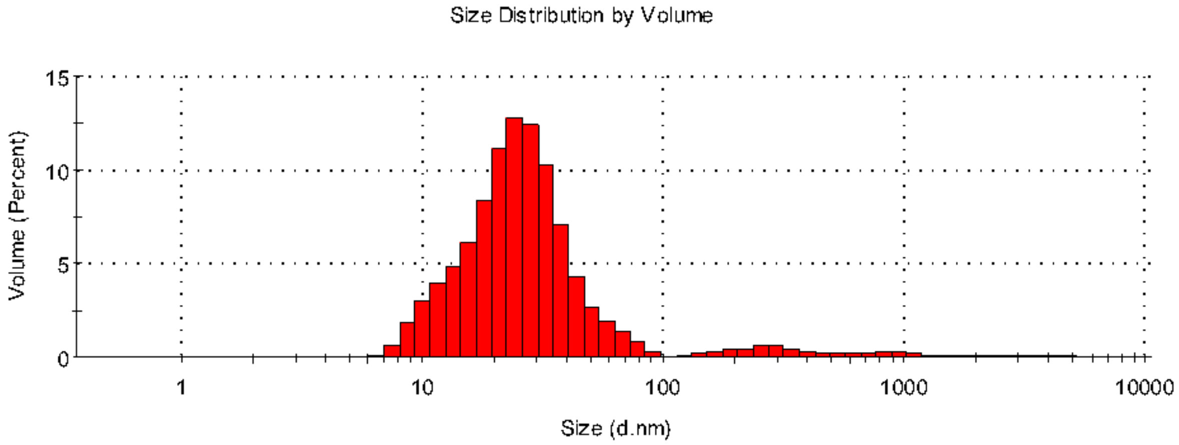

Figure S73: Histogram of the number percentage of particle size (d.nm) of water after processing in the VFD, flat base VFD tube (20 mm OD, 17.5 mm ID, 18.5 cm in length), ω, 5250 rpm, tilt inclination, θ, +45° for 15 minutes processing time with a 1:1 mixture with chlorobenzene, immediately after processing.

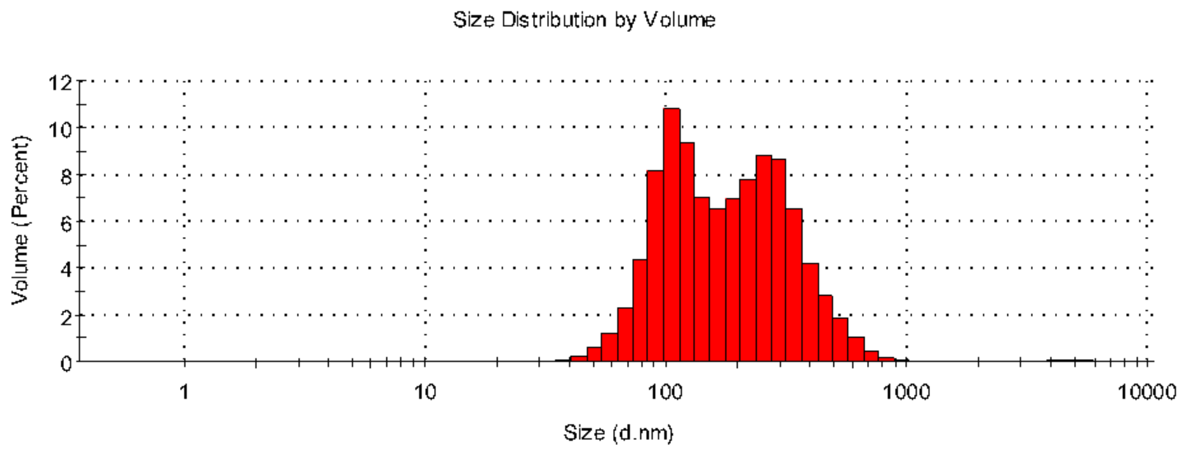

Figure S74: Histogram of the number percentage of particle size (d.nm) of water after processing in the VFD, flat base VFD tube (20 mm OD, 17.5 mm ID, 18.5 cm in length), ω, 5250 rpm, tilt inclination, θ, +45° for 15 minutes processing time with a 1:1 mixture with chlorobenzene, 24 hours after processing.

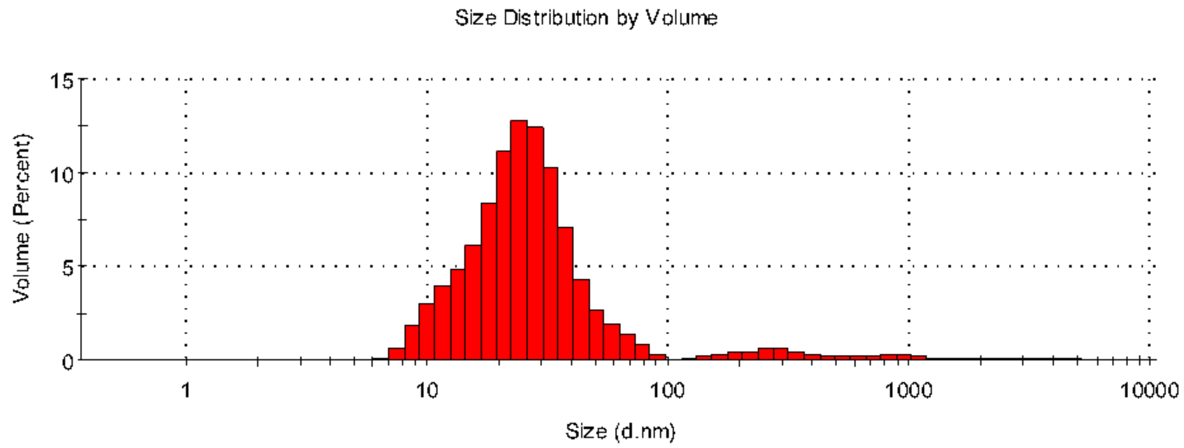

Figure S75: Histogram of the number percentage of particle size (d.nm) of chlorobenzene after processing in the VFD, flat base VFD tube (20 mm OD, 17.5 mm ID, 18.5 cm in length), **ω,** 5250 rpm, tilt inclination, θ, +45° for 15 minutes processing time with a 1:1 mixture with water, immediately after processing.

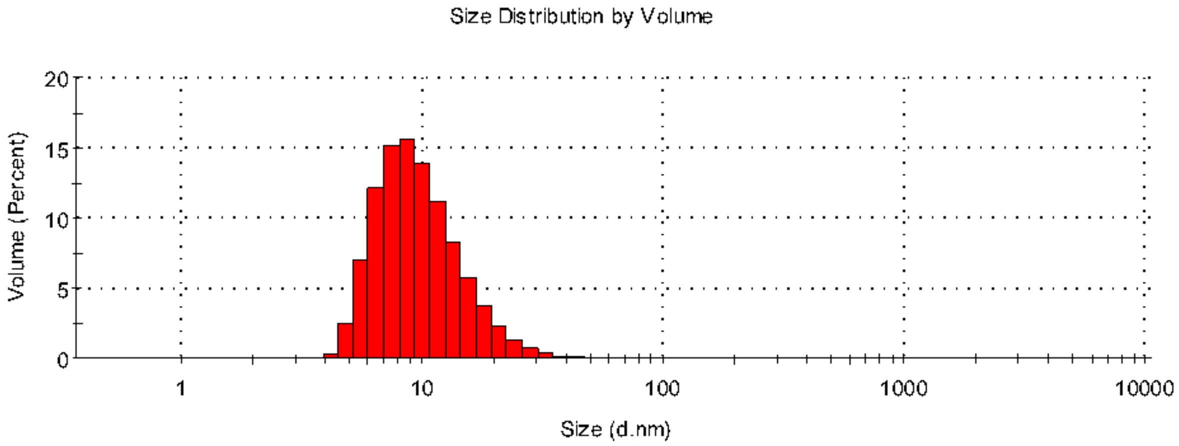

Figure S76: Histogram of the number percentage of particle size (d.nm) of chlorobenzene after processing in the VFD, flat base VFD tube (20 mm OD, 17.5 mm ID, 18.5 cm in length), ω, 5250 rpm, tilt inclination, θ, +45° for 15 minutes processing time with a 1:1 mixture with water, 24 hours after processing.

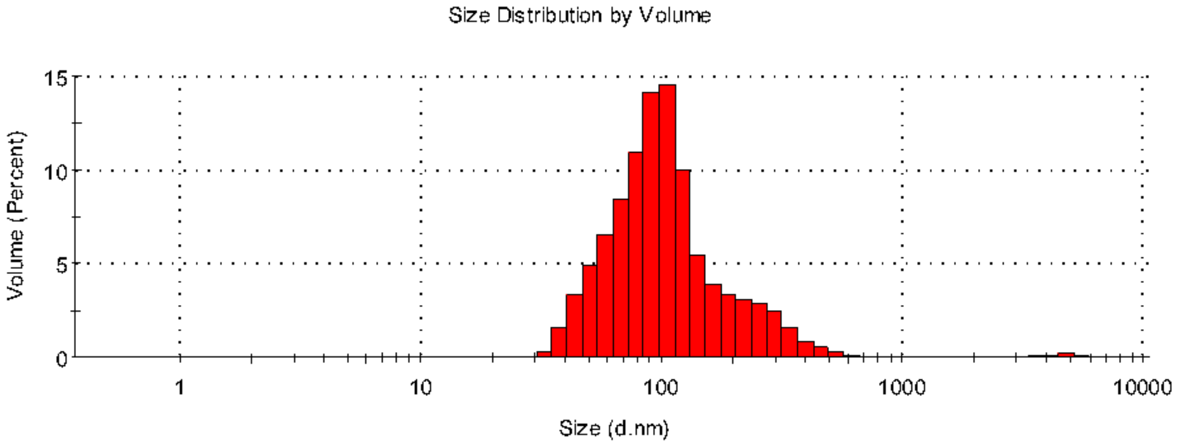

Figure S77: Histogram of the number percentage of particle size (d.nm) of water after processing in the VFD, flat base VFD tube (20 mm OD, 17.5 mm ID, 18.5 cm in length), ω, 7500 rpm, tilt inclination, θ, +45° for 15 minutes processing time with a 1:1 mixture with t chlorobenzene, immediately after processing.

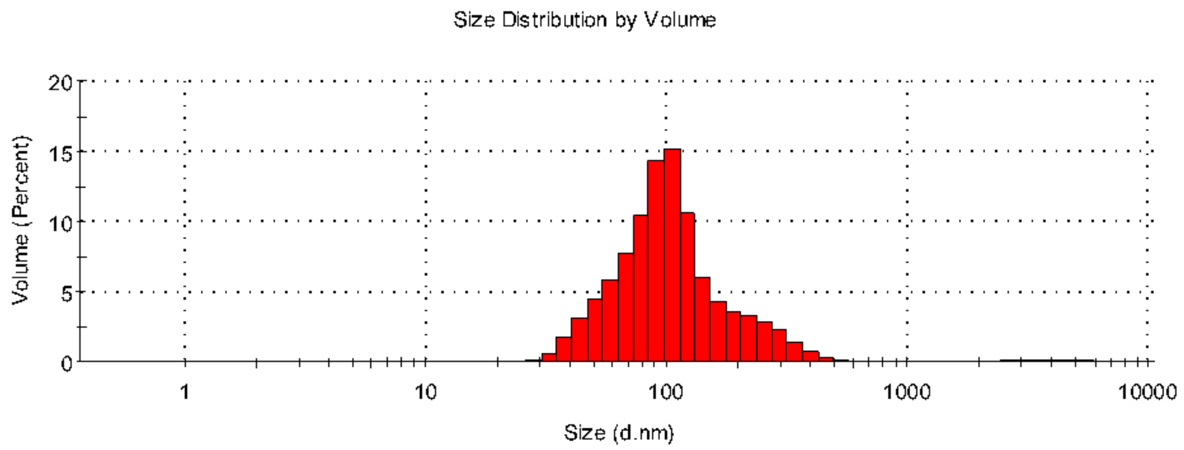

Figure S78: Histogram of the number percentage of particle size (d.nm) of water after processing in the VFD, flat base VFD tube (20 mm OD, 17.5 mm ID, 18.5 cm in length), ω, 7500 rpm, tilt inclination, θ, +45° for 15 minutes processing time with a 1:1 mixture with chlorobenzene, 24 hours after processing.

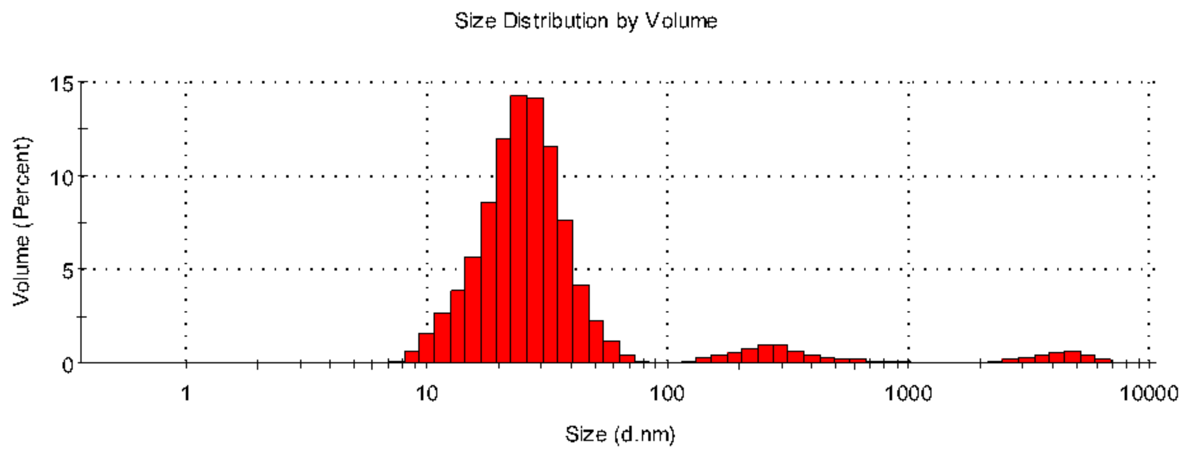

Figure S79: Histogram of the number percentage of particle size (d.nm) of chlorobenzene after processing in the VFD, flat base VFD tube (20 mm OD, 17.5 mm ID, 18.5 cm in length), ω, 7500 rpm, tilt inclination, θ, +45° for 15 minutes processing time with a 1:1 mixture with water, immediately after processing.

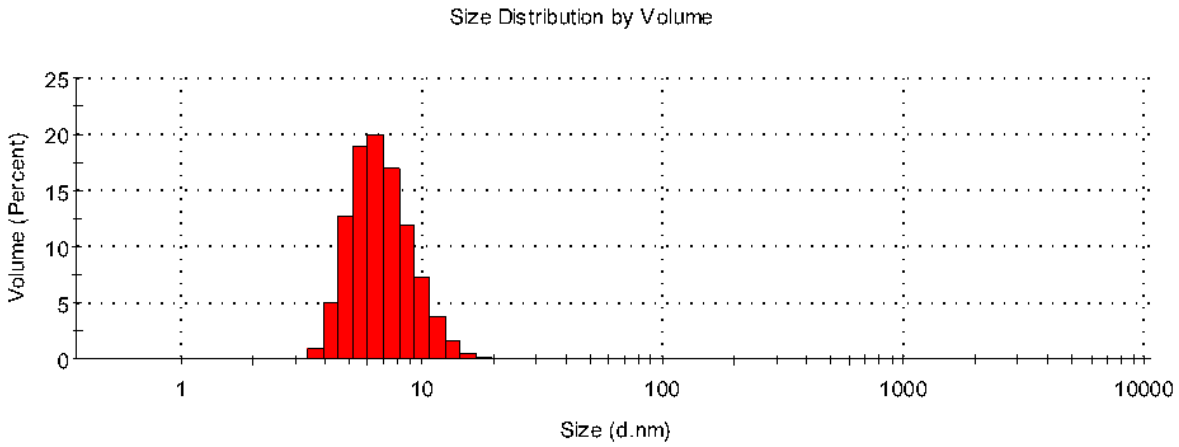

Figure S80: Histogram of the number percentage of particle size (d.nm) of chlorobenzene after processing in the VFD, flat base VFD tube (20 mm OD, 17.5 mm ID, 18.5 cm in length), ω, 7500 rpm, tilt inclination, θ, +45° for 15 minutes processing time with a 1:1 mixture with water, 24 hours after processing.

S6. Interfacial self-assembly of nanoparticles

1. General materials and methods

Oleic acid coated magnetite nanoparticles were prepared by chemical co-precipitation. Briefly, 2.7 g $FeCl_3 \cdot 6H_2O$ and 1 g $FeCl_2 \cdot 4H_2O$ were dissolved in 100 mL MilliQ water under a flow of N_2. The solution was stirred and heated to 80°C. A total of 43 mL NaOH (1 M) was added with a final pH of 12.1. 6.3 mL of oleic acid was then added, and the mixture was maintained at 80°C for another 60 min. The mixture was cooled to room temperature naturally and the black product was collected using a magnet and thoroughly washed with ethanol and deionised water to remove excess non-reactants and freeze-dried. The biphasic experiments were conducted in either VFD with hemisperical based tube (20 mm OD, 17.5 mm ID, length 19.3 cm), flat based tube (20 mm OD, 17 mm ID length 19 cm) or long hemispherical based tube (20 mm OD, 17.1 mm ID, length 39 cm). The control epxeirment without using VFD was carried out in a benchtop vortex mixer. Optimal condition for interaface $Cu_3(PO_4)_2$@Magnetite film generation is by adding 5 mL toluene (with oleic acid coated magnetite nanoparticles at 0.2 mg/mL) to 5 mL PBS (10 mM) followed by brief bath sonication, quick addition of 536 μL $CuSO_4$ and immediate VFD processing at 7500 rpm for 30 min or benchtop vortex mixing for 30 min.

Post-VFD processing, toluene and PBS were spontaneously demixed where a thin film named $Cu_3(PO_4)_2$@Magnetite film (Cu@MF) was observed in the interface and $Cu_3(PO_4)_2$@Magnetite nanoflower (Cu@MNF) in the PBS phase (Figure 4). In comparison, flat-based VFD tube or benchtop vortex was unable to generate Cu@MF from the interface (Figure S81b,d). The morphology of the Cu@MF could be varied by changing the volume of starting material and the length of the VFD tubes. When increasing the volume mixture from 3 to 10 mL, the Cu@MF became less dense and ended up being spider-web-like (Figure S81a,c).

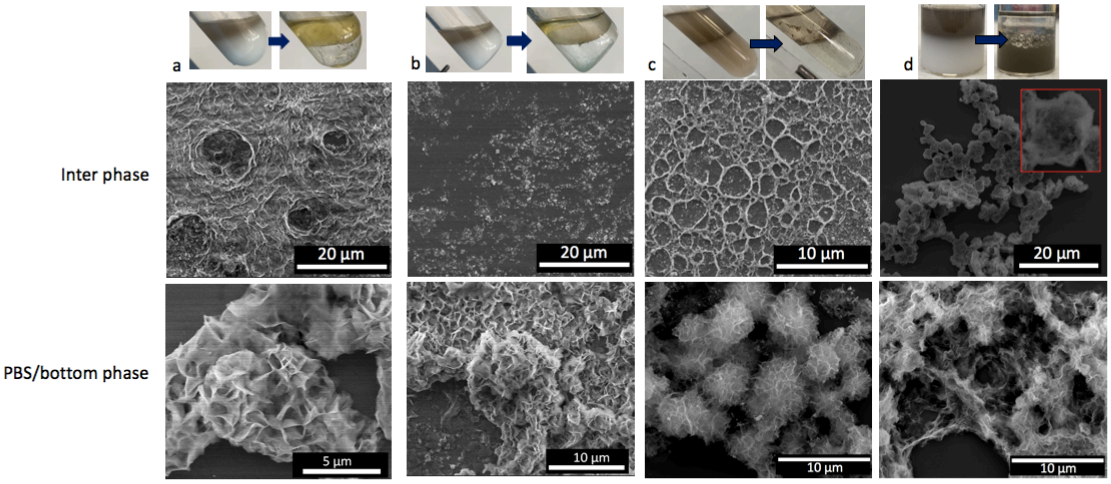

Figure S81: Fabrication of $Cu_3(PO_4)_2$@Magnetite thin film (Cu@MF) and $Cu_3(PO_4)_2$@Magnetite nanoflower (Cu@MNF) in a biphasic system of toluene and phosphate buffer saline (PBS) using different VFD tubes (a) hemispherical-based VFD tube (20 mm o.d., 17.5mm i.d., length 19.3 cm) with 3 mL biphasic mixture and 160.8 µL $CuSO_4$ processed at 7500 rpm for 30 min (b) flat-based VFD tube with 3 mL (20 mm o.d., 17 mm i.d., length 19 cm) biphasic mixture and 160.8 µL $CuSO_4$ processed at 7500 rpm for 30 min (c) long hemispherical-based VFD tube (20 mm o.d. 17.1 mm i.d., length 39 cm) with 10 mL biphasic mixture and 536 µL $CuSO_4$ processed at 7500 rpm for 30 min (d) benchtop vortexing with 10 mL biphasic mixture and 536 µL $CuSO_4$ for 30 min. The biphasic system was briefly sonicated followed by quick addition of $CuSO_4$ and immediate processing as described. SEM showed a typical structure obtained from the interface or the PBS/bottom phase. Photos indicated pre and post processing of the biphasic system using each method.

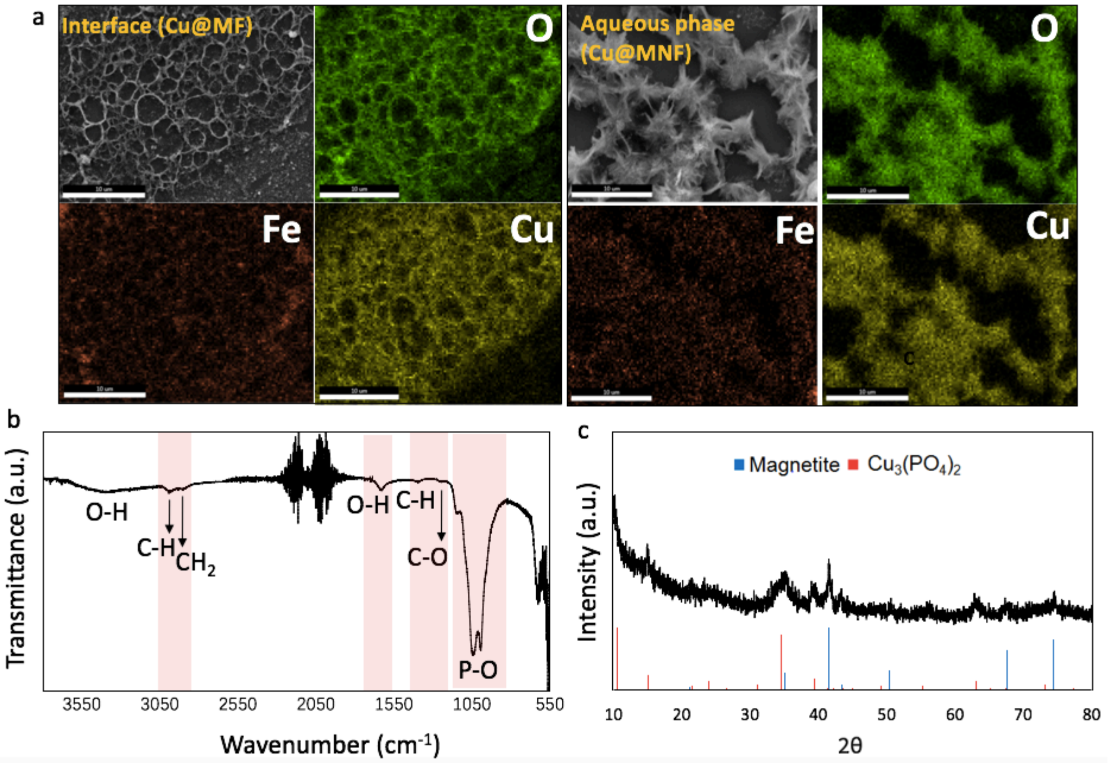

Figure S82: SEM/EDX of the Cu@MF and Cu@MNF by using long hemispherical-based VFD tube with elemental mapping images for O, Fe and Cu, respectively. (b) FT-IR spectra of the Cu@MNF. (c) XRD of Cu@MNF.

S6. Biphasic synthesis

General

Thin layer chromatography (TLC) was conducted on Merck Kieselgel 60 F254 plates and products were visualised under shortwave UV light (254 nm). ^{1}H- and ^{13}C nuclear magnetic resonance spectra were obtained using a Bruker AV-500 spectrometer (500 MHz for ^{1}H and 125 MHz for ^{13}C). Each sample was dissolved in d-chloroform and each spectrum was calibrated using the residual solvent peak (δ 7.26 for ^{1}H and δ 77.16 for ^{13}C).

Optimisation of reaction conditions for the coupling of 2-acetamido-3,4,6-tri-*O*-acetyl-α-D-glucopyranosyl chloride with 4-nitrophenol

A 20 mm quartz tube (20 mm OD, 17.5 mm ID quartz tube, 18.5 cm long) was charged with 4-nitrophenol (15 mg, 0.11 mmol) and sodium hydroxide solution (0.22M, 0.5 mL) and to this solution was added a solution of 2-acetamido-3,4,6-tri-*O*-acetyl-α-D-glucopyranosyl chloride[2] (40 mg, 0.11 mmol) in CH_2Cl_2 (0.5 mL). The tube was capped tightly then rotated in the VFD at 2000, 3500, 5000, 7000, or 9000 rpm with a tilt angle of 30, 45 or 60° for 60 minutes. The mixture was then diluted with EtOAc (20 mL) and the resulting organic phase was successively washed with water, brine, dried ($MgSO_4$), filtered, and concentrated to yield a solid. Flash chromatography (EtOAc:hexanes, 7:3) of the residue gave the title compound. The ^{1}H and ^{13}C NMR spectra of the obtained compound was consistent with the literature[3].

^{1}H NMR of 4-nitrophenyl 2-acetamido-3,4,6-tri-O-acetyl-2-deoxy-β-D-glucopyranoside

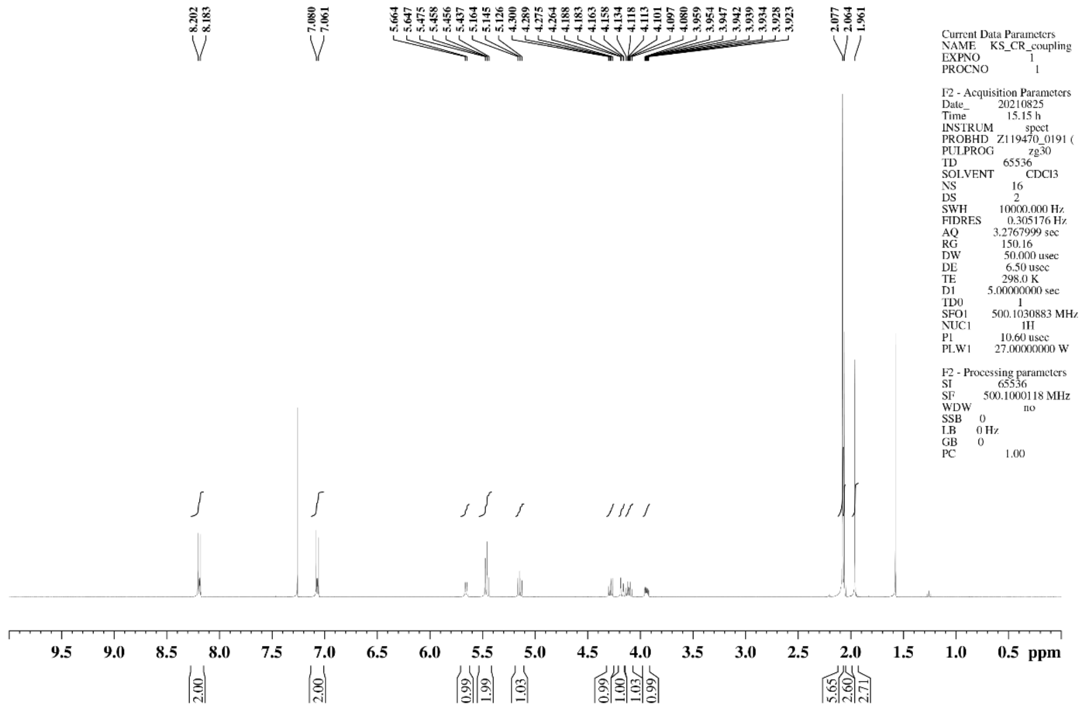

^{13}C NMR of 4-nitrophenyl 2-acetamido-3,4,6-tri-*O*-acetyl-2-deoxy-β-D-glucopyranoside

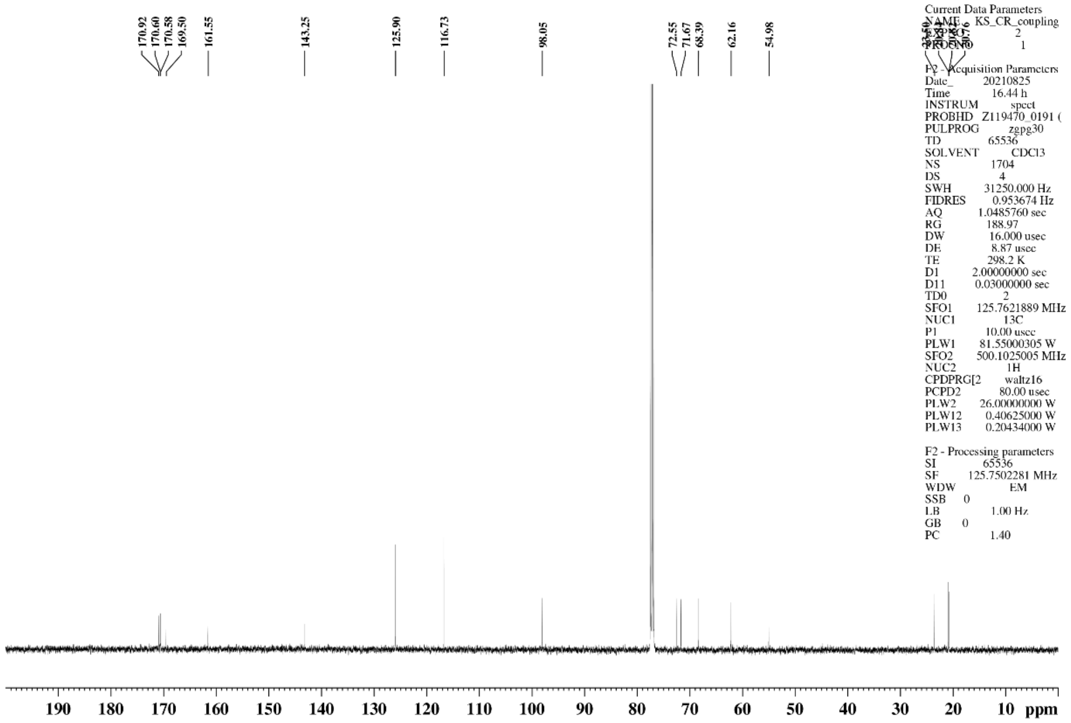

S7. References

[1] T. M. D. et al. Sub-micron moulding angled vortex fluid flow, *Nanoscale Advances* 2021, **3**, 3064-3075.

[2] R. Roy and F. D. Tropper, Carbohydrate protein interactions. syntheses of agglutination inhibitors of wheat germ Agglutinin by phase transfer catalysis. *Can. J Chem.* 1991, **69**, 817-821.

[3] T. A. Chupakhina et al., Aromatic Crown Ethers as Phase Transfer Catalysts in the Synthesis of *N*-Acetylglucosamine β-Aryl Glycosides**,** *Russian Journal of Bioorganic Chemistry*, 2004, **30**, 301–303.
